# Discovery of new symmetrical and asymmetrical nitrile-containing 1,4-dihydropyridine derivatives as dual kinases and P-glycoprotein inhibitors: synthesis, *in vitro* assays, and *in silico* studies

**DOI:** 10.1080/14756366.2022.2120478

**Published:** 2022-09-12

**Authors:** Mohamed H. Saad, Tarek F. El-Moselhy, Nabaweya S, El-Din, Ahmed B. M. Mehany, Amany Belal, Mohammed A. S. Abourehab, Haytham O. Tawfik, Mervat H. El-Hamamsy

**Affiliations:** a The Egyptian Ministry of Health, Tanta, Egypt; b Department of Pharmaceutical Chemistry, Faculty of Pharmacy, Tanta University, Tanta, Egypt; c Zoology Department, Faculty of Science, Al-Azhar University, Cairo, Egypt; d Medicinal Chemistry Department, Faculty of Pharmacy, Beni-Suef University, Beni-Suef, Egypt; e Department of Pharmaceutical Chemistry, College of Pharmacy, Taif University, Taif, Saudi Arabia; f Department of Pharmaceutics, Faculty of Pharmacy, Umm Al-Qura University, Makkah, Saudi Arabia; g Department of Pharmaceutics and Industrial Pharmacy, College of Pharmacy, Minia University, Minia, Egypt

**Keywords:** 1,4-DHPs, anticancer, multidrug resistance (MDR), P-glycoprotein, receptor tyrosine kinases (RTKs), apoptosis, antimicrobial

## Abstract

Two new series of symmetric (**1a-h)** and asymmetric (**2a-l)** 1,4-DHP derivatives were designed, synthesised, and evaluated as anticancer agents. *In vitro* anticancer screening of target compounds *via* National cancer institute “NCI” revealed that analogues **1g**, **2e**, and **2l** demonstrated antiproliferative action with mean growth inhibition percentage “GI%” = 41, 28, and 64, respectively. The reversal doxorubicin (DOX) effects of compounds **1g**, **2e**, and **2l** were examined and illustrated better cytotoxic activity with IC_50 _=1.12, 3.64, and 3.57 µM, respectively. The most active anticancer analogues, **1g**, **2e**, and **2l**, were inspected for their putative mechanism of action by estimating their epidermal growth factor receptor (EGFR), human epidermal growth factor receptor 2 (HER-2), and Bruton’s tyrosine kinase (BTK) inhibitory activities. Furthermore, the antimicrobial activity of target compounds was assessed against six different pathogens, followed by determining the minimum inhibitory concentration “MIC” values for the most active analogues. Molecular docking study was achieved to understand mode of interactions between selected inhibitors and different biological targets.

## 1. Introduction

Cancer is still the second leading cause of death worldwide, despite advances in its treatment and prevention. However, the effectiveness of cancer treatment in the twenty-first century is still a worry, and new and safer anticancer drugs with a broader range of cytotoxicity to tumour cells need to be researched.^1^^,^^2^ Many types of cancers arise as a result of the ability of cells to proliferate indefinitely and their inherent resistance to apoptosis. Tumour cells have the potential to stimulate their self-proliferation *via* biological pathways, including meiotic cell division, which contributes to carcinogenesis.^3^^,^^4^ Relying on new evidence, kinases proteins such as receptor tyrosine kinases (RTKs) and cyclin-dependent kinases (CDKs) are the most extensively studied targets in diverse carcinogenic signalling pathways because they have been involved in highly conserved physiological processes such as cell proliferation, mitosis, and cell division.^5^^,^^6^ Aberrant or excessive production of these proteins disrupts the normal regulation of the cell cycle, which is seen in a variety of cancers. Accordingly, kinases inhibitors interfere directly with biological processes, preventing excessive cell proliferation and tumour progression.^7^^,^^8^ It was found that 1,4-dihydropyridines (1,4-DHPs) could be promising RTK and CDK inhibitors.^9–11^

**Figure 1.**
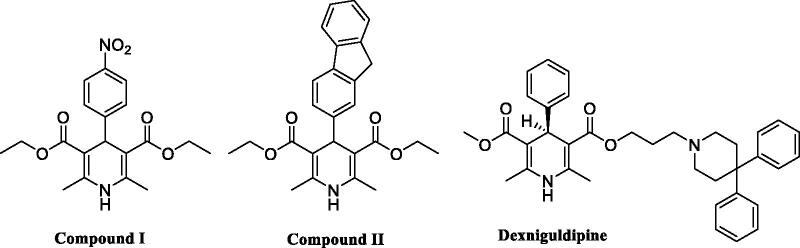
Structure of some reported biologically active 1,4-DHP derivatives.

There are many 1,4-DHPs with variable structures that showed anticancer activity in many cancer cell lines, where they could disrupt the survival of tumour cells by different mechanisms.^12^ Compound **I** having 1,4-DHP ring, displayed apoptotic effect in human melanoma; A375 cell line, and human liver cancer; HepG2 cell line through its capability of binding to the allosteric site of SIRT1, resulting in its activation^13^^,^^14^ as illustrated in Figure 1. Whereas activation of NAD^+^-dependent deacetylase SIRT1 inhibits tumorigenesis by promoting apoptosis by suppressing the transcription factor NF-κB. Compound **II** demonstrated good RTK inhibitory activity, exhibiting IC_50_=68.80, 71.79, and 84.51 nM against Bruton’s tyrosine kinase (BTK), epidermal growth factor receptor (EGFR), and human epidermal growth factor receptor 2 (HER-2), respectively, compared to erlotinib, IC_50_ = 59.41, 49.13, and 74.73 nM respectively,^15^ as shown in Figure 1.

Unfortunately, multidrug resistance (MDR) in cancer is a pleiotropic complex phenomenon by which some drug-resistant tumour cells gain the ability to overcome the effect of unrelated chemotherapeutic agents regardless of their chemical structure and mechanism of action. So, MDR is considered the major obstacle in cancer treatment nowadays. Many mechanisms are involved in cancer MDR;^16^^,^^17^ the most important one is ATP binding cassette (ABC) protein transporters, specially permeation protein (P-glycoprotein [P-gp]).^18^^,^^19^

1,4-DHPs such as dexniguldipine (Figure 1) was one of the potent P-gp inhibitors lacking calcium antagonistic activity.^20^^,^^21^ Dexniguldipine is the (−)-enantiomer of the asymmetric 1,4-DHP, niguldipine, that is 40 times less potent than (+)-niguldipine in its interaction with L-type calcium channels. Hence, structural modifications on the 1,4-DHP scaffold were performed to generate 1,4-DHP derivatives having better P-gp inhibitory activity and less calcium antagonistic activity. A series of 1,4-DHP niguldipine analogues were synthesised, and their effects on daunomycin cytotoxicity against HCT-116/ADR (a resistant variant of the human breast cancer HCT-116 cell line over expresses P-gp) were investigated.^22^ It was reported that 1,4-DHPs compounds lacking or having low calcium channel antagonistic activity retained MDR reversal property, which was dependent on the nature of the substituent at 3- and 5- positions of the DHP ring. Ester groups at 3- and 5- positions of the DHP ring were essential for calcium channel antagonistic activity, thus, replacement of these groups could reduce cardiovascular side effects and improve the MDR reversal property.^23^

Moreover, 1,4-DHP is one of the important chemical nucleuses which are incorporated in the synthesis of new antimicrobial agents. Many 1,4-DHP compounds showed different patterns of antimicrobial activity against different types of bacteria^24^ and fungi.^25^^,^^26^ Some compounds disclosed activity against gram-positive bacteria and have no effect on gram-negative ones.^27^ Symmetric 1,4-DHP analogues were more effective against gram-positive bacteria than gram-negative ones.^28^ Patients with cancer may have a higher risk of infection because of changes in the immune system that control their body’s defence systems. Cancer and cancer treatments can affect the immune system and other body systems in different ways.^29–31^ Therefore, it is desirable that drugs used in the treatment of cancer have an effective impact on the prevention or treatment of secondary microbial infection.^32–34^ Consequently, we have designed and synthesised two series of 1,4-DHP derivatives as potential anticancer agents with the privilege of having antimicrobial activity.

## 2. Rationale design

**Figure 2.**
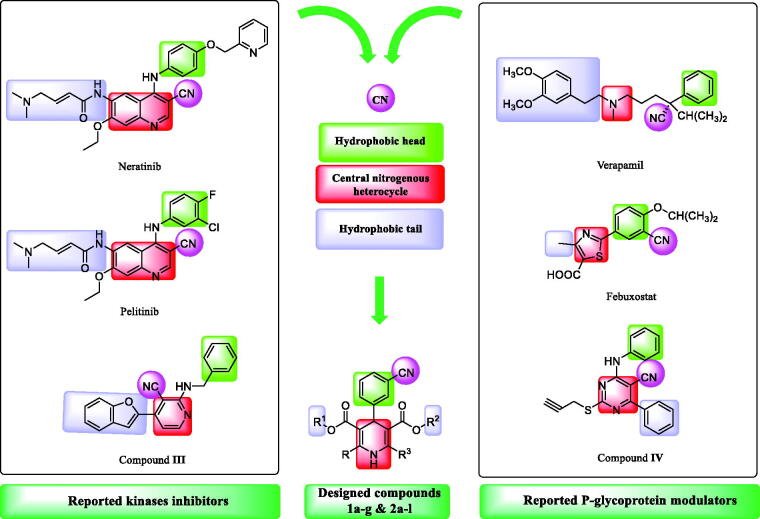
The rationale for the molecular design of the target compounds as kinases and P-gp dual inhibitors.

Recently, the nitrile group has become increasingly relevant in medicinal chemistry.^35^ Since 2010, the US Food and Drug Administration (US FDA) has authorised at least one nitrile-containing drug per year, with five drugs approved in 2020^36^. Chronic myeloid leukaemia, breast cancer, fungal infection, and other clinical disorders are all targeted by these marketed drugs having a nitrile moiety. Nitrile-containing drugs may have greater pharmacokinetic and pharmacological effects than other pharmaceuticals with similar therapeutic effects due to the unique physicochemical features of the nitrile group.^35–37^ The second-generation EGFR kinase inhibitors, including Neratinib and Pelitinib, and compound **III**, are nitrile-containing tyrosine kinase inhibitors,^38^ as illustrated in Figure 2. The majority of these inhibitors have electrophilic Michael-acceptor moieties that may interact covalently with cysteine amino acid at the lip of the EGFR’s ATP binding cleft, inactivating the protein.^39^^,^^40^ Additionally, the following are some pharmacophoric features shared by all EGFR-inhibitors: (i) The presence of a hetero aromatic system in the adenine binding pocket. (ii) Inserting the hydrophobic terminal head in the hydrophobic area I. (iii) The amino group which has the potential to establish significant hydrogen bonds with amino acid residues in the linker region. (iv) A hydrophobic tail that is found in hydrophobic region II.^41^^,^^42^ Concerning Verapamil, Febuxostat, and compound **IV,**^43^^,^^44^ the nitrile moiety partakes a notable effect as a P-gp modulator.^45^^,^^46^ Additionally, 5-oxo-hexahydroquinoline bearing the pyridyl alkyl carboxylate moieties at position 3 are better inhibitors of P-gp than the compounds having carboxamide substituents that give good P-gp modulators. Alkyl and hetero aromatic insertions at the C4 position would reduce activity, whereas lipophilic aromatic insertions of CN, NO_2_, and halogens groups would increase MDR reversal activity because of their electron-withdrawing substitutions.^47^ The integration of the hydrophobic tail, central nitrogenous heterocycle, and hydrophobic head alongside the nitrile group were the key to build up our target compounds with dual activity *via* matching the pharmacophoric properties of kinases inhibitors and p-gp modulators, as verified in Figure 2. Relying on the aforementioned outcomes, we have designed 1,4-dihydropyridine derivatives comprising the nitrile group (**1a-h** and **2a-l**) as dual inhibitors of kinases and p-gp (Figure 2).

It was also found that the nitrile group connected to the dihydropyridine has an effective role as an antimicrobial.^48–52^ Several novel series of nitrile-containing antimicrobial compounds with weakly basic amines are reported, which have reduced the potential for hERG (human ether-a-go-go gene) channel inhibition.^53^

## 3. Results and discussion

### 3.1. Chemistry

**Scheme 1.**
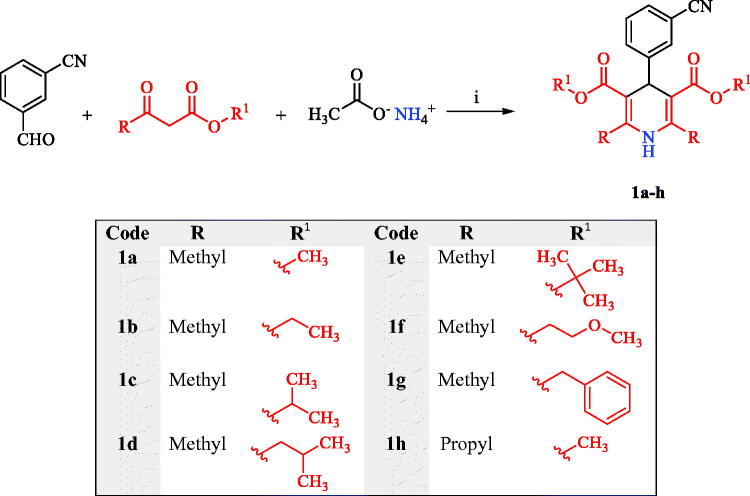
Synthesis of the first series of symmetric achiral 1,4-DHP compounds, **1a-h**. Reagents and conditions: (i) MeOH, heat under reflux, 24 h.

Two novel series of 1,4-DHP derivatives were designed and synthesised. The first series comprised eight symmetric achiral 1,4-DHPs, while the second series enclosed twelve asymmetric chiral 1,4-DHPs. The first series, eight symmetric achiral 1,4-DHPs **1a-h**, was synthesised by the classical Hantsch reaction for direct synthesis of symmetric 1,4-DHP^54^ *via* the classical Hantsch reaction between 3-cyanobenzaldehyde, acetoacetic esters, and ammonium acetate as illustrated in Scheme 1.

**Scheme 2.**
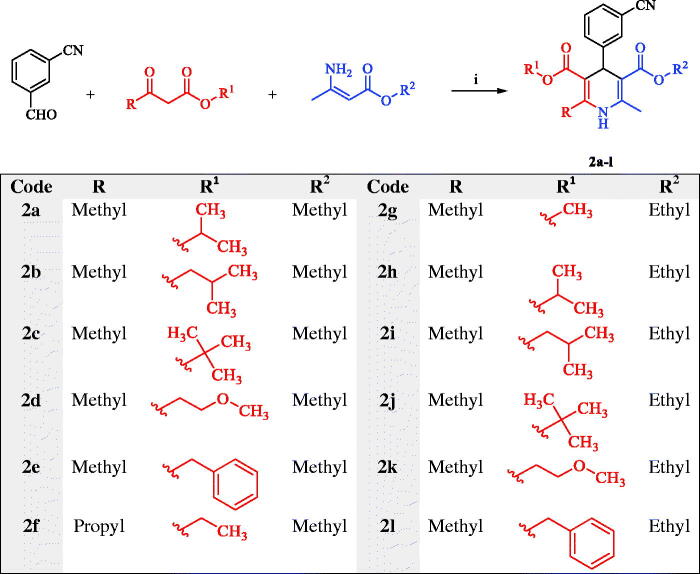
Synthesis of the second series, asymmetric chiral 1,4-DHP analogues **2a-l**. Reagents and conditions: (i) MeOH, heat under reflux, 24 h.

The second series, twelve asymmetric chiral 1,4-DHPs **2a-l**, was synthesised *via* Iwanami reported method^55^^,^^56^ through cyclocondensation of 3-cyanobenzaldehyde, acetoacetic esters, and alkyl 3-aminocrotonates as revealed in Scheme 2. Compounds **2a-l** were prepared as racemic mixtures.

Chemical structures of target compounds were confirmed by elemental analysis, ^1^H, ^13^C NMR, and mass spectroscopy. ^1^H NMR spectra showed characteristic peaks for each proton in the synthesised compounds, as reported in the experimental section. ^1^H NMR spectra of target compounds were characterised by two singlet signals of C_4_-H and NH protons in the 1,4-DHP ring at δ 4.88–5.09and δ 5.59–6.10 ppm, respectively. Meanwhile, the disappearance of singlet corresponding to -CHO proton of 3-cyanobenzaldehyde, as well as the singlet signal of active methylene protons in β-ketoesters, were established. ^13^C NMR spectroscopy was performed on selected compounds, **1f**, **1g**, **1h**, **2b**, **2j**, and **2l,** which exposed the characteristic peaks for each carbon as given in the experimental section.

### 3.2. Pharmacological evaluation of target compounds

#### 3.2.1. Anticancer activity

##### 3.2.1.1. *In vitro* anticancer screening at NCI-USA

All target compounds were selected and submitted to the National Cancer Institute (NCI; www.dtp.nci.nih.gov), Bethesda, Maryland, USA, relying on the diversity of structures and computer modelling techniques for the assessment of their anticancer activity. The 20 compounds were screened at a single dose of 10 µM concentration against sixty cell lines of nine different types of human tumours, including leukaemia, non-small cell lung cancer, colon cancer, central nervous system cancer, melanoma, ovarian cancer, renal cancer, prostate cancer, and breast cancer, according to NCI developmental therapeutics programme.^57^^,^^58^

###### 3.2.1.1.1. *In vitro* single dose (10µM) anticancer screening on NCI 60 cancer cell lines

Target compounds were subjected to *in vitro* NCI anticancer assay at 10 µM concentration. According to 48h drug exposure protocol, the tested compound was exposed to the cultures. Endpoint determinations were performed using a sulforhodamine B (SRB) assay to assess cell growth and viability. The results of all tested compounds were reported as a mean graph of the percentage growth (G%) of the tested cells compared to the unprocessed control cells and relative to the time zero number of cells^10^ (see supplementary material) and displayed as percentage growth inhibition (GI%) caused by the tested compounds as shown in Table 1. GI% value was calculated by subtracting the corresponding G% value from 100.

**Table 1.**
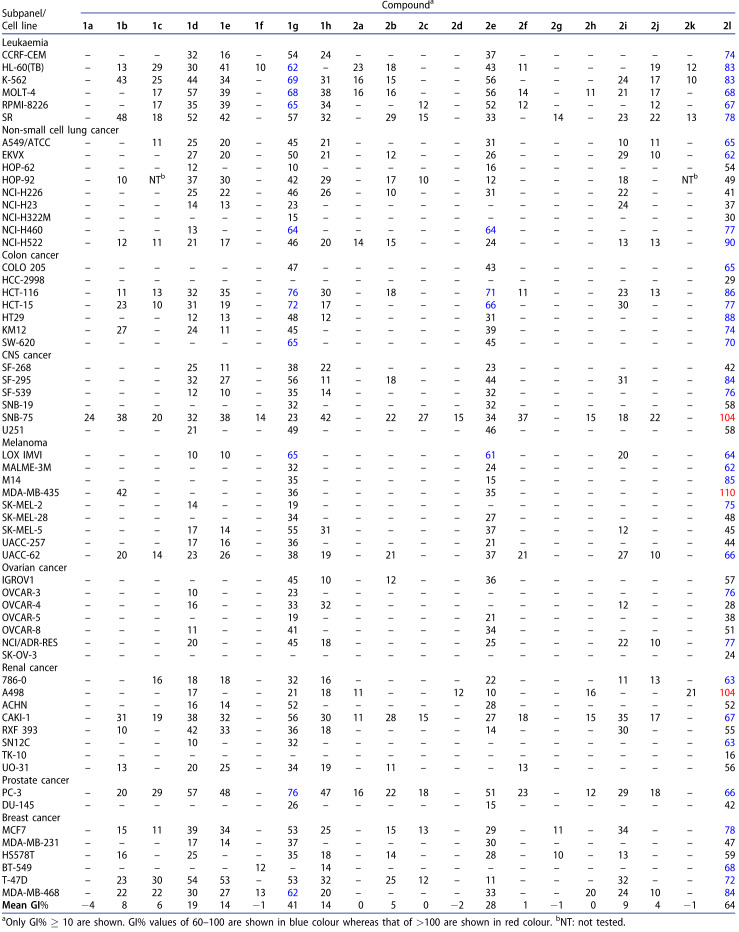
*In vitro* cytotoxic activity, GI%, for compounds **1a-h** and **2a-l** towards 60 subpanel cancer cell lines at 10 µM concentration.

Inspecting the data illustrated in Table 1 revealed that the tested compounds exposed different profiles of antiproliferative activity. Compound **2l,** mean GI%=64, exhibited strong anticancer activity, while **1g** and **2e** showed moderate activity, mean GI%=41 and 28, respectively. Other compounds showed weak to very weak activity, exhibiting a mean GI% value below 20. Compound **2l** showed the most significant antiproliferative activity relative to other synthesised compounds. Analogue **2l** retained broad-spectrum antitumor activity against all subpanels of all tested 60 cancer cell lines. Compounds **1g** and **2e** also possessed broad-spectrum antitumor activity, but against only 56 and 49 subpanels, respectively. Compound **2l** showed excellent growth inhibitory activity against leukaemia (HL-60(TB) and K-562), non-small cell lung cancer (NCI-H522), colon cancer (HCT-116 and HT29), CNS cancer (SF-295), melanoma (M14), and breast cancer (MDA-MB-468) with GI% of 83, 83, 90, 86, 88, 84, 85, and 84, respectively. In terms of lethality, **2l** was the only compound displaying the lethal effect against only 3 subpanels: CNS cancer (SNB-75), melanoma (MDA-MB-435), and renal cancer (A498) with GI%=104, 110, and 104, respectively.

####### 3.2.1.1.1.1. Structure–activity relationship (SAR) relying on the results of anticancer screening on NCI 60 cancer cell lines

The second series of asymmetric chiral analogues **2a-l**, mean GI% ranged from −2 to 64, exposed to better anticancer activities than symmetric achiral compounds **1a-h**, mean GI% ranged from −4 to 41, in the first series.

1. Concerning the first series of symmetric 1,4-DHPs **1a-h**, the existence of benzyl esters at both C3 and C5 of 1,4-DHP ring in **1g**, mean GI% = 41, enriched anticancer activity and presented the strongest analogue **1g**, compared to alkyl esters analogues which reported GI% = −4 - 19. Increasing the size and lipophilicity of the alkyl chain of ester groups at C3- and C5-positions improved antitumor activity in the order: **1d**, GI% = 19 > **1e**, GI% = 14 > **1b**, GI% = 8 > **1c**, GI%=6 which hold the alky groups: isobutyl, *tert*-butyl, ethyl, and isopropyl, respectively. The presence of dimethyl esters and dimethoxyethyl esters abolished the anticancer activity of analogues, **1a**, GI% = −4 and **1f**, GI% = −1, respectively.

Regarding alkyl groups at C2 and C6 of the DHP ring, elongation of the alkyl chain enhanced antitumor activity, where **1h**, GI% = 14, with two propyl groups at C2 and C6 was more active than its analogue **1b**, GI% = 8, having two methyl groups instead of propyl.

**Figure 3.**
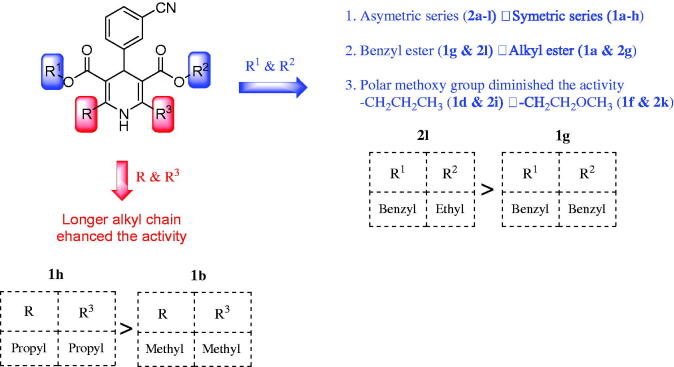
Summary of SAR for anticancer activity of target compounds besides structures of the most active analogues.

2. In the second series of asymmetric and chiral analogues, **2a-l**, the type of esters at C3 and/or C5 of the DHP ring significantly controlled the anticancer activity. The presence of benzyl ester at C3 and ethyl ester at C5 furnished the most active analogue **2l**, mean GI% = 64. Introduction of benzyl ester at C3 strongly enhanced the activity of **2e**, mean GI% = 28, as well compared to compounds bearing aliphatic esters, which revealed GI% = −2 − 9. Reducing the size and lipophilicity of alkyl esters at C3 or C5 diminished the anticancer activity in the order: **2i**, GI% = 9 > **2b**, GI% = 5 > **2j**, GI% = 4 > **2f**, GI% = 1 which have alkyl groups: isobutyl, isobutyl, *tert*-butyl, and ethyl, respectively. The methoxyethyl ester abolished the activity of **2k**, GI% = −1. Compounds **2g-l**, GI% = −1 − 64, bearing an ethyl ester group were more active than analogues **2a-f**, GI% = −2 – 28, having a methyl ester group at C3 of DHP ring. SAR of target compounds and the most active analogues are illustrated in Figure 3.

##### 3.2.1.2. *In vitro* cytotoxicity of target compounds, 1g, 2e, and 2l in human HCT-116 and HCT-116/ADR cells and their potentiation of DOX cytotoxicity in drug-resistant HCT-116/ADR cells

**Figure 4.**
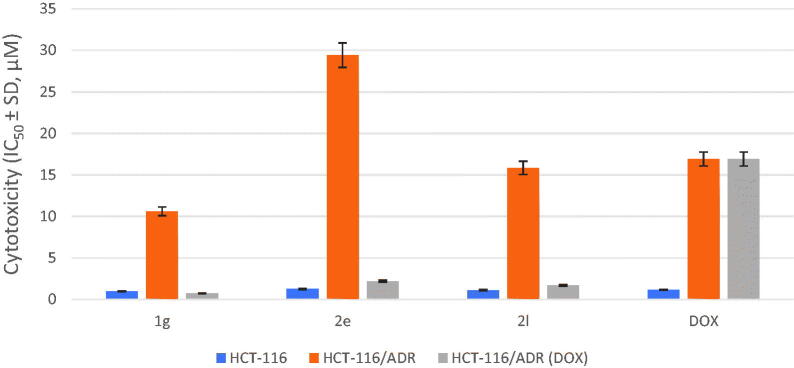
Cytotoxicity (µM) of compounds **1 g**, **2e**, and **2 l** on human HCT-116, HCT-116/ADR (without DOX), and HCT-116/ADR (with DOX) cells.

The most observable difference between human breast cancer cells, HCT-116 and drug resistance HCT-116/ADR strains, is the overexpression of P-gp in resistant strains only.^59^^,^^60^ Potential inhibitors of P-gp should not be recognised or effluxed by P-gp because they are not P-gp substrates. We have selected the most active anticancer target compounds, **1g**, **2e**, and **2l,** to investigate their reversal doxorubicin (DOX) effect *via* evaluating the corresponding IC_50_ of DOX and calculating reversal-folds (RF) by dividing the IC_50_ (DOX) without P-gp modulator by IC_50_ (DOX) with P-gp modulators, **1g**, **2e**, and **2l**. Compound **1g,** with two benzyl esters at C3 and C5, displayed more potential and reversal activity (IC_50_ (DOX)=0.72 µM, RF = 23.48) than analogues with one benzyl ester group, **2e** with IC_50_ (DOX)=2.20 µM, RF = 7.68, and **2l** with IC_50_ (DOX) = 1.68 µM, RF = 10.05. Accordingly, tested target compounds inhibited P-gp and significantly augmented the cytotoxic effect of DOX against the drug resistance HCT-116/ADR cancer cells, as informed in Table 2 and Figure 4.

**Table 2.** Cytotoxicity, IC_50_^a^, of compounds **1 g**, **2e**, and **2 l** against human HCT-116 and HCT-116/ADR cells and their potentiation of DOX cytotoxicity in drug-resistant HCT-116/ADR cells.

Compound	HCT-116	HCT-116/ADR	HCT-116/ADR (DOX)	RF^b^
**1g**	0.99 ± 0.58	10.61 ± 0.10	0.72 ± 0.001	23.48
**2e**	1.27 ± 0.42	29.41 ± 0.45	2.20 ± 0.015	7.68
**2l**	1.11 ± 0.45	15.83 ± 0.13	1.68 ± 0.010	10.05
DOX^c^	1.15 ± 0.43	16.91 ± 0.14	16.91 ± 0.14	1

^a^Data are the mean of three-independent trials of triplicate experiments. ^b^Reversal fold activity. ^c^Doxorubicin without modulator.

##### 3.2.1.3. Mechanistic insight of 1g, 2e, and 2l induced cytotoxicity

**Figure 5.**
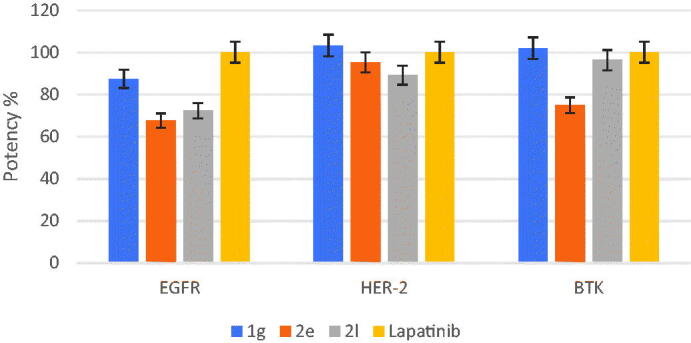
% Potency of compounds **1 g**, **2e**, and **2 l** against EGFR, HER-2, and BTK.

The potent antitumor agents in this study were subjected to further studies to investigate their potential mechanism of action.^15^^,^^61^ Epidermal growth factor receptor (EGFR) kinase, HER-2 kinase, and Bruton’s tyrosine kinase (BTK) inhibitory activity of the most potent antitumor compounds **1g**, **2e**, and **2l** were studied, and results were demonstrated as IC_50_ (nM) (Table 3) and % potency of EGFR, HER-2, and BTK (Figure 5), and they were compared to lapatinib as a reference drug. Compound **1g** established the highest EGFR, HER-2, and BTK inhibitory activity with IC_50 _= 62.19 ± 0.76, 70.55 ± 0.63, and 60.27 ± 0.42 nM, respectively, which was higher or close to that of lapatinib with IC_50_ = 54.38 ± 0.7, 72.81 ± 0.68, and 61.47 ± 0.58 nM, respectively. In addition, compounds **2e** and **2l** displayed promising EGFR inhibitory activity with IC_50_ = 80.35 ± 1.26 (for **2e**) and 75.22 ± 0.95 (for **2l**), HER-2 inhibitory activity with IC_50_ = 76.54 ± 0.87 (for **2e**) and 81.72 ± 0.92 (for **2l**), and BTK inhibitory activity with IC_50_ = 82.06 ± 1.40 (for **2e**) and 63.80 ± 0.55 (for **2l**).

**Table 3.** Cytotoxicity (IC_50_ ± SD_,_ nM) and Potency (%)* of **1 g**, **2e**, and **2 l** on EGFR, HER-2, and BTK compared with lapatinib.

Compound	EGFR		HER-2		BTK	
	IC_50_ ± SD	Potency	IC_50_ ± SD	Potency	IC_50_ ± SD	Potency
**1g**	62.19 ± 0.76	87.44	70.55 ± 0.63	103.20	60.27 ± 0.42	101.99
**2e**	80.35 ± 1.26	67.68	76.54 ± 0.87	95.13	82.06 ± 1.40	74.91
**2l**	75.22 ± 0.95	72.29	81.72 ± 0.92	89.10	63.80 ± 0.55	96.35
Lapatinib	54.38 ± 0.7	100.0	72.81 ± 0.68	100.0	61.47 ± 0.58	100.0

*Potency (%) was calculated as ((IC_50_ lapatinib/IC_50_ Compound) *100).

##### 3.2.1.4. Annexin V–FITC apoptosis assay

Apoptosis induction is the most important mechanism by chemotherapeutics killing tumour cells.^62^^,^^63^ Phosphatidylserine (PS) is translocated from the inside to the outside of the plasma membrane during apoptosis, causing cellular alterations. Annexin-V binds to PS and can be employed as a sensitive probe on the plasma membrane’s outer side.^64^^,^^65^ We used the annexin V–fluorescein isothiocyanate (FITC)/propidium iodide (AV/PI) dual-staining test with the BD FACS Calibur to discriminate apoptosis from necrosis in colon cancer HCT-116 cells (the most affected cancer cell lines when treated with analogues **1g**, **2e,** and **2l** at the NCI) death mediated by the most active compounds **1g**, **2e**, and **2l** (BD Biosciences, San Jose, CA).

**Figure 6.**
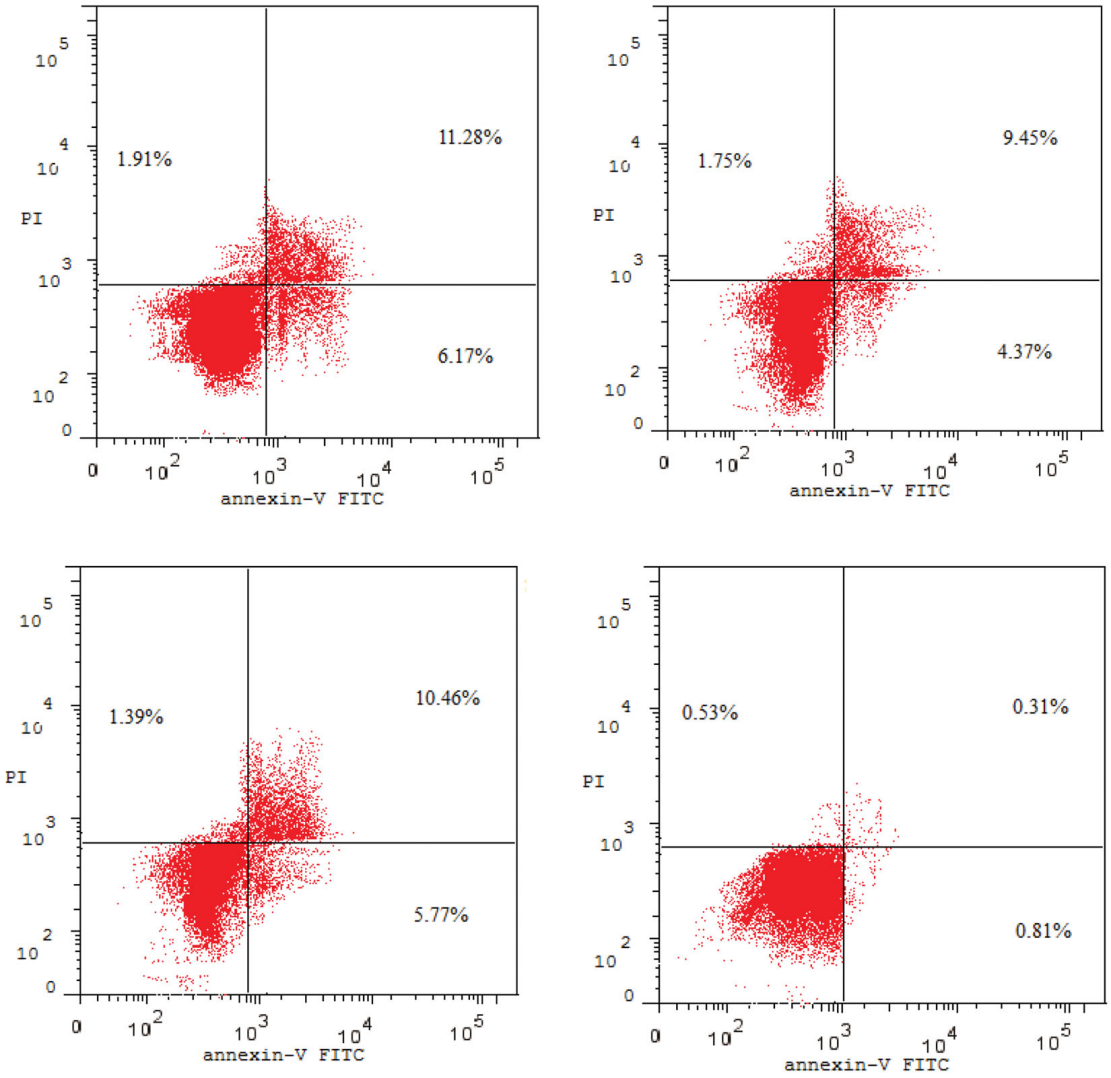
Apoptosis assay: Impact of compound **1 g** (upper left), compound **2e** (upper right), compound **2 l** (lower left), and DMSO (lower right) on the % of annexin V-FITC-positive staining in HCT-116 cells.

**Figure 7.**
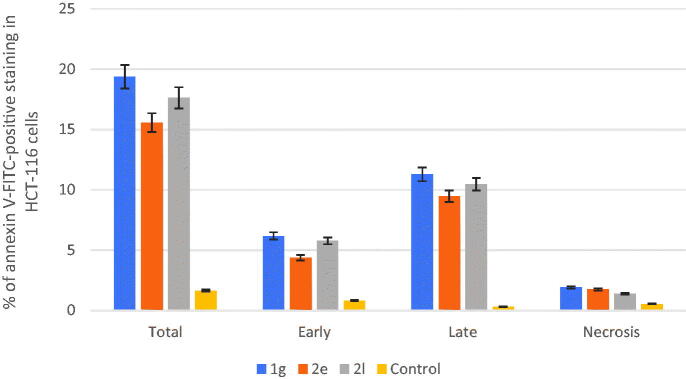
Effect of compounds **1 g**, **2e**, and **2 l** and DMSO on the percentage of HCT-116 cells stained positive for annexin V-FITC in apoptosis assay.

HCT-116 cells were stained with AV/PI for 24h at a mixed molar concentration of 10 µM with each of compounds **1g**, **2e**, and **2l**. The results of treating HCT-116 cells with each of compounds **1g**, **2e**, and **2l** for 24h were shown in Figures 6 and 7.

The early apoptosis ratio (Figure 6, lower-right quadrant of cytogram) increased from 0.81% in the control sample (dimethyl sulphoxide [DMSO]) to the range 4.37–6.17%, whereas the late apoptosis ratio (Figure 6, upper-right quadrant of cytogram) increased sharply from 0.31% to 9.45–11.28%. These findings point to an apoptotic mechanism rather than a necrotic pathway as the cause of compounds **1g**, **2e**, and **2l**-induced programmed cell death.

##### 3.2.1.5. *In vitro* cell cycle analysis

**Figure 8.**
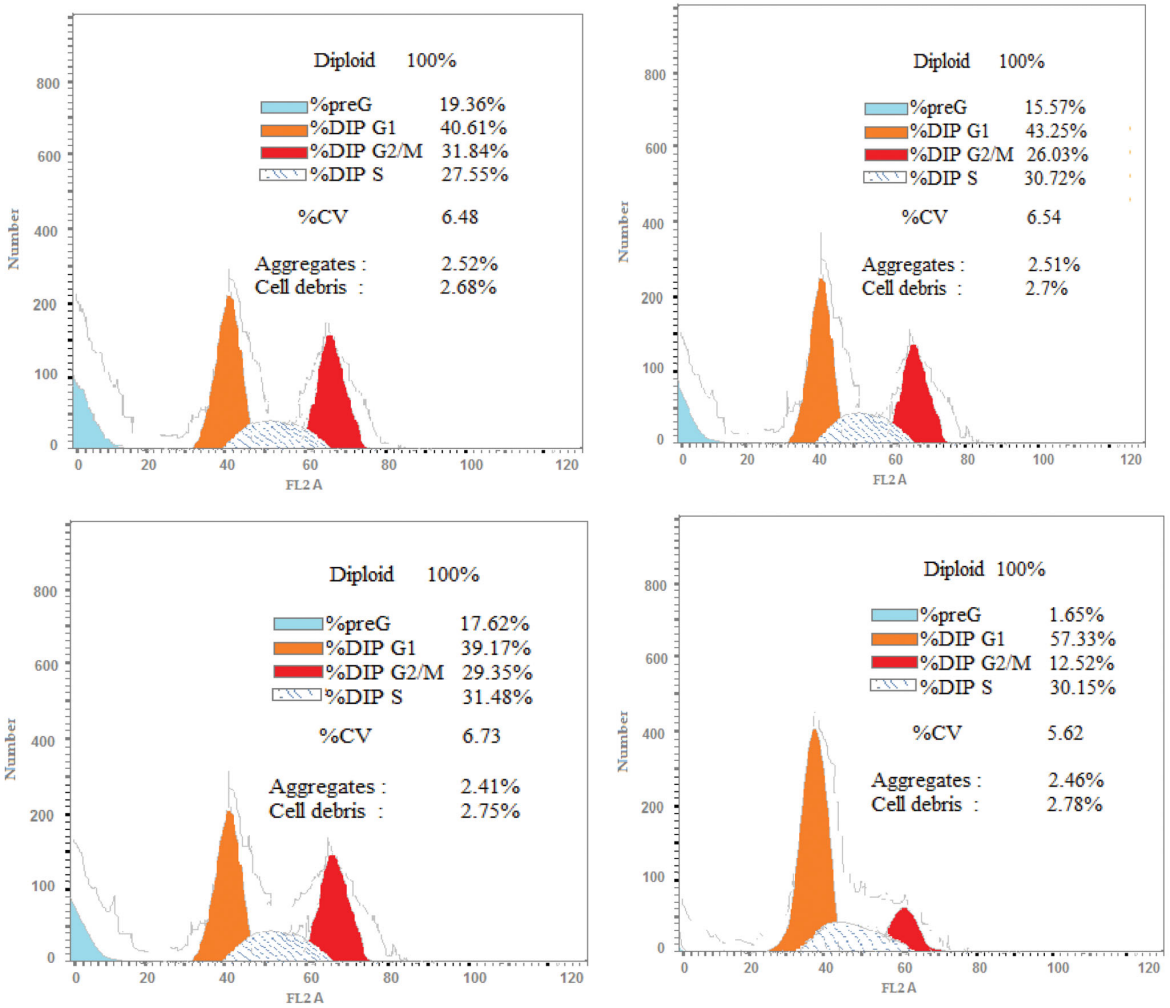
Cell cycle analysis of HCT-116 cells treated with compound **1 g** (upper left panel), compound **2e** (upper right panel), compound **2 l** (lower left panel), and DMSO (lower right panel).

**Figure 9.**
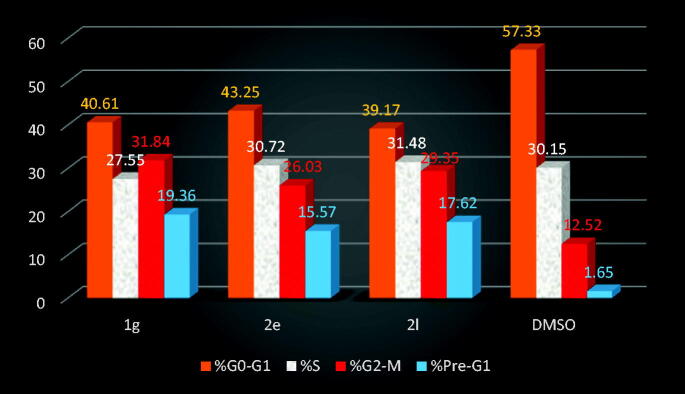
Outcome of compounds **1 g**, **2e**, and **2 l** and DMSO on the percentage of HCT-116 cells cell cycle analysis.

Targeting the cancer cell cycle has been developed as a encouraging approach for cancer therapy.^66^ DNA flow cytometry analysis was used to analyse the influence of compounds **1g**, **2e**, and **2l** on activation of the cell cycle in HCT-116 cells to determine the role of these compounds in cancer cell growth suppression and, as a result, induction of apoptosis in different phases. HCT-116 cells were treated for 24h with compounds **1g**, **2e**, and **2l** in comparison to DMSO, stained with PI, flow cytometrically evaluated, and the results were shown in Figures 8 and 9. With a concurrent reduction in the G0/G1 phase, 39.17–43.25% for compounds **1g**, **2e**, and **2l** compared to the control (57.33%), a significant rise in the proportion of apoptotic cells was found at the pre-G1 phase (15.57–19.36% on exposure to **1g**, **2e**, and **2l**) compared to control (1.65%). Furthermore, as demonstrated in Figures 8 and 9, a significant increase in cells in the G2/M phase was observed, with 26.03–31.84% for **1g**, **2e**, and **2l** and 12.52% for control, showing marked cell arrest in the G2/M phase.

#### 3.2.2. Antimicrobial activity

##### 3.2.2.1. *In vitro* antibacterial and antifungal evaluation

Target compounds were assessed *in vitro* for their antimicrobial activity *via* the agar well diffusion method^51^^,^^67^ against six pathogens; two gram-positive bacteria (*Staphylococcus aureus* and *Bacillus subtilis*), two gram-negative bacteria (*Escherichia coli* and *P. Pseudomonas aeruginosa*), and two fungi (*Candida albicans* and *Aspergillus flavus*). The results of antimicrobial activity expressed as the average diameter of inhibition zone (DIZ) and the calculated % activity index (AI) for target compounds are reported in Table 4. 1,4-DHP derivatives, **1a-h** and **2a-l**, showed better inhibition activity towards Gram-positive than against Gram-negative bacteria. The second series of asymmetric 1,4-DHPs, **2a-l**, revealed DIZs = 6–22 and 7–21 mm against G positive and G negative bacteria, respectively, which were better than the symmetric series of 1,4-DHPs, **1a-h** which reported DIZs = DIZs= 6–19 and 7–17 mm against G positive and G negative bacteria, respectively, while ciprofloxacin, DIZs = 23–26 mm, was used as a reference drug. Moreover, analogues **2a-l**, DIZs = 7–24 mm, displayed stronger antifungal activity than **1a-h,** DIZs = 8–19 mm compared to clotrimazole, DIZs = 25–27 mm.

**Table 4.** *In vitro* antimicrobial activity of compounds **1a-h** and **2a-l** against six different pathogenic strains of bacteria and fungi using agar well diffusion method.

Compound	Diameter of inhibition zone^a^ in mm (% activity index^b^)					
	Bacteria				Fungi	
	G (+) bacteria		G (–) bacteria			
	*S. aureus*	*B. subtilis*	*E. coli*	*P. aeruginosa*	*C. albicans*	*A. flavus*
**1a**	13 (54.2)	9 (39.1)	8 (30.8)	12 (52.2)	13 (48.1)	15 (60)
**1b**	14 (58.3)	9 (39.1)	7 (26.9)	10 (43.5)	15 (55.5)	16 (64)
**1c**	11 (45.8)	6 (26.1)	–	8 (34.8)	10 (37)	13 (52)
**1d**	–	–	–	–	–	–
**1e**	–	–	–	–	–	–
**1f**	**19 (79.2)**	**16 (69.6)**	**13 (50)**	**17 (73.9)**	**18 (66.7)**	**19 (76)**
**1g**	15 (62.5)	11 (47.8)	10 (38.5)	16 (69.6)	10 (37)	14 (56)
**1h**	7 (29.2)	–	–	–	–	8 (32)
**2a**	**20 (83.3)**	**17 (73.9)**	**14 (53.8)**	**19 (82.6)**	**21 (77.8)**	**22 (88)**
**2b**	9 (37.5)	–	9 (34.6)	13 (56.5)	7 (25.9)	11 (44)
**2c**	–	–	–	–	–	7 (28)
**2d**	15 (62.5)	10 (43.5)	10 (38.5)	14 (60.9)	12 (44.4)	15 (60)
**2e**	8 (33.3)	–	–	7 (30.4)	–	10 (40)
**2f**	6 (25)	–	–	–	–	–
**2g**	**22 (91.7)**	**19 (82.6)**	**19 (73.1)**	**21 (91.3)**	**23 (85.2)**	**24 (96)**
**2h**	11 (45.8)	8 (34.8)	–	10 (43.5)	8 (29.6)	12 (48)
**2i**	–	–	–	–	–	–
**2j**	**18 (75)**	**15 (65.2)**	**18 (69.2)**	**20 (86.9)**	**19 (70.4)**	**20 (80)**
**2k**	17 (70.8)	13 (56.5)	15 (57.7)	20 (86.9)	16 (59.2)	18 (72)
**2l**	–	–	–	–	–	–
Ciprofloxacin	24 (100)	23 (100)	26 (100)	23 (100)	NT	NT
Clotrimazole	NT	NT	NT	NT	27 (100)	25 (100)

^a^Values are means of three replicates. ^b^Values below 25 are of limited value and not shown as they refer either to inactive or non-diffusing compounds.

NT: not tested.

Target compounds demonstrated a higher inhibition effect against *S. aureus* than *B. subtilis* mimic the reference drug, ciprofloxacin. On the other hand, the compounds’ activity was higher against *P. aeruginosa* than that against *E. coli* in contrast to ciprofloxacin which was more active against *E. coli*. The antifungal activity of all active compounds was higher against *A. flavus* than *C. albicans* in contrast to the reference drug, clotrimazole which was more active against *C. albicans*. Regarding the activity against Gram-positive bacteria, compounds **2a** and **2g** showed the highest inhibition effect, AI = 83 and 91 against *S. aureus,* and AI = 73 and 82 against *B. subtilis*, respectively. Compounds **1f** and **2j** showed good antibacterial activity, AI = 79 & 75 against *S. aureus*, respectively. Concerning the activity against Gram-negative bacteria, compounds **2g** and **2j** demonstrated the highest inhibition effect, AI = 91 and 86 towards *P. aeruginosa* and AI = 73 and 69 against *E. coli*, respectively. The inhibitory effect of target compounds against fungi revealed that analogues **2a**, **2g**, and **2j** presented the highest antifungal activity, AI =88, 96, and 80 towards *A. flavus* and AI = 77, 85, and 70 against *C. albicans*, respectively. Accordingly, the best antibacterial activity was observed in the presence of analogue **2g**, asymmetric DHP, and compound **1f,** symmetric DHP, towards the six tested pathogens.

We have successfully synthesised compound **1g**, which revealed a remarkable mutual activity as an anticancer (mean GI% =41) and antimicrobial agent. Analogue **1g** displayed antimicrobial effects against six different pathogenic strains of bacteria and fungi (Table 4). However, it turned out that compound **2e** (mean GI%= 28) moderately affected three pathogenic strains of bacteria and fungi. Unfortunately, the best anticancer among the series, compound **2l** (mean GI%= 64), was inactive against tested pathogenic strains (Table 4).

##### 3.2.2.2. Determination of minimum inhibitory concentration (MIC)

The most active analogues **1f**, **1g**, **2a**, **2g**, **2j**, and **2k**, which demonstrated the strongest inhibition effect against tested pathogens, were selected for further determination of their MIC values, Table 5, *via* the microbroth dilution method.^68^ Compound **2g** disclosed MIC = 0.5 and 1.0 µg/mL against G-positive bacteria, *S. aureus* and *B. subtilis*, respectively, which was two**-**fold more potent than ciprofloxacin, MIC = 1.0 and 2.0 µg/mL. Compound **2a** was equipotent to ciprofloxacin against both G-positive bacteria, MIC = 1.0 and 2.0 µg/mL against *S. aureus* and *B. subtilis*, respectively. The six compounds were less potent than ciprofloxacin against G-negative bacteria. Regarding activity against fungi, the most active compound, **2g**, was two-fold more potent than clotrimazole against *C. albicans*, MIC = 1.0 µg/mL. Therefore, **2g** was the most active antibacterial analogue in this series, as mentioned before. Upon all of the above findings, synthesised 1,4-DHP derivatives are promising candidates for the development of more active antibacterial and antifungal agents.

**Table 5.** Antibacterial activity expressed as minimum inhibitory concentration (MIC) for analogues **1f**, **1 g**, **2a**, **2 g**, **2j**, and **2k** evaluated by the broth microdilution method.

Compound	MIC (µg/mL)					
	Bacteria				Fungi	
	G (+) bacteria		G (–) bacteria			
	*S. aureus*	*B. subtilis*	*E. coli*	*P. aeruginosa*	*C. albicans*	*A. flavus*
**1f**	**4**	**2**	**8**	**16**	**16**	**16**
**1g**	64	32	32	32	64	>64
**2a**	**1**	**2**	**2**	**4**	**4**	**8**
**2g**	**0.5**	**1**	**1**	**2**	**1**	**2**
**2j**	8	4	1	4	16	8
**2k**	16	8	4	8	32	32
Ciprofloxacin	1	2	0.5	1	NT	NT
Clotrimazole	NT	NT	NT	NT	2	1

NT: not tested.

### 3.3. Molecular docking study

**Figure 10.**
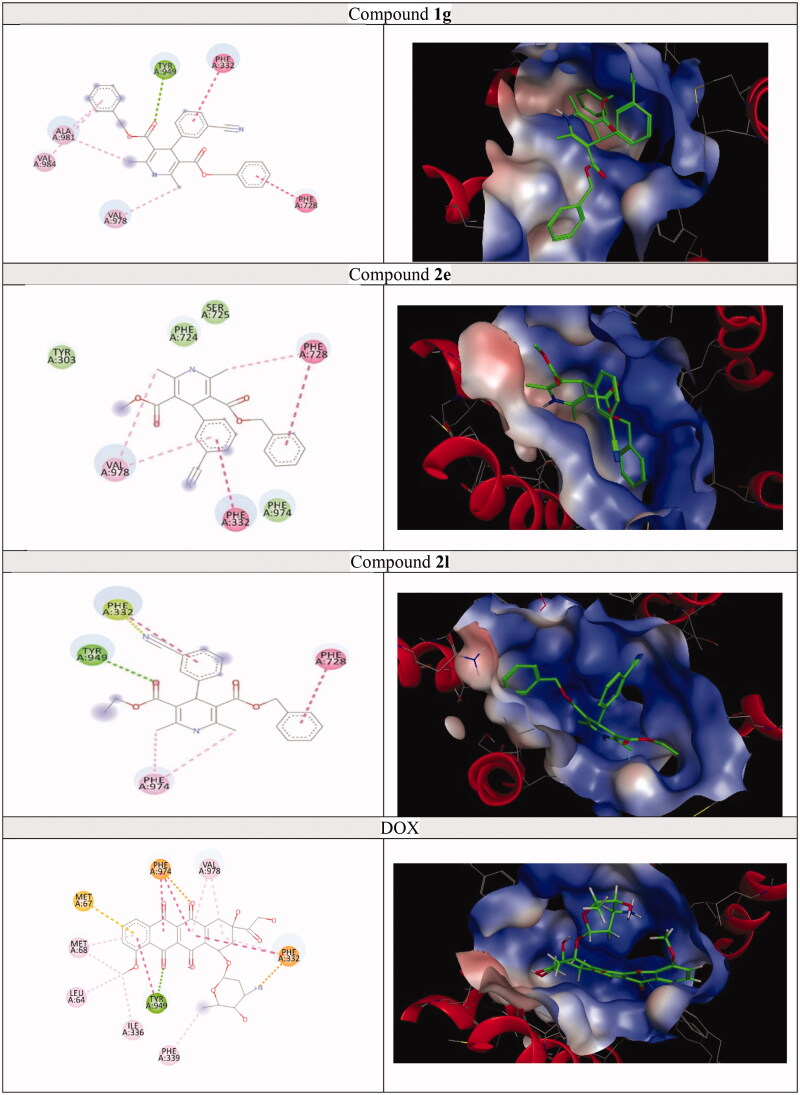
The 2D and 3D docking poses of compounds **1g**, **2e**, **2l** and DOX interactions with P-gp (PDB ID: 3G60).

**Figure 11.**
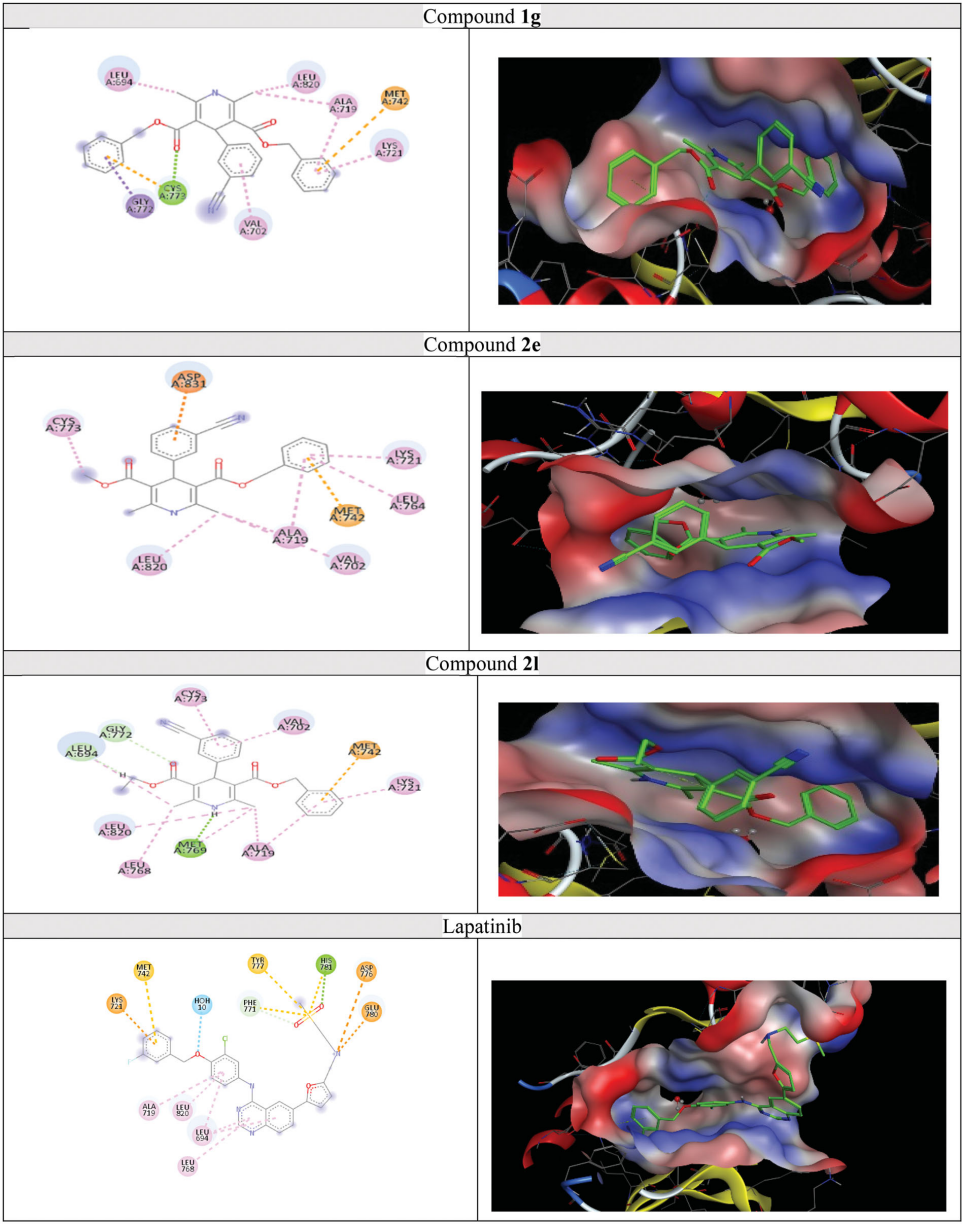
The 2D and 3D docking poses of compounds **1g**, **2e**, **2l** and lapatinib interactions with EGFR (PDB ID: 1M17).

**Figure 12.**
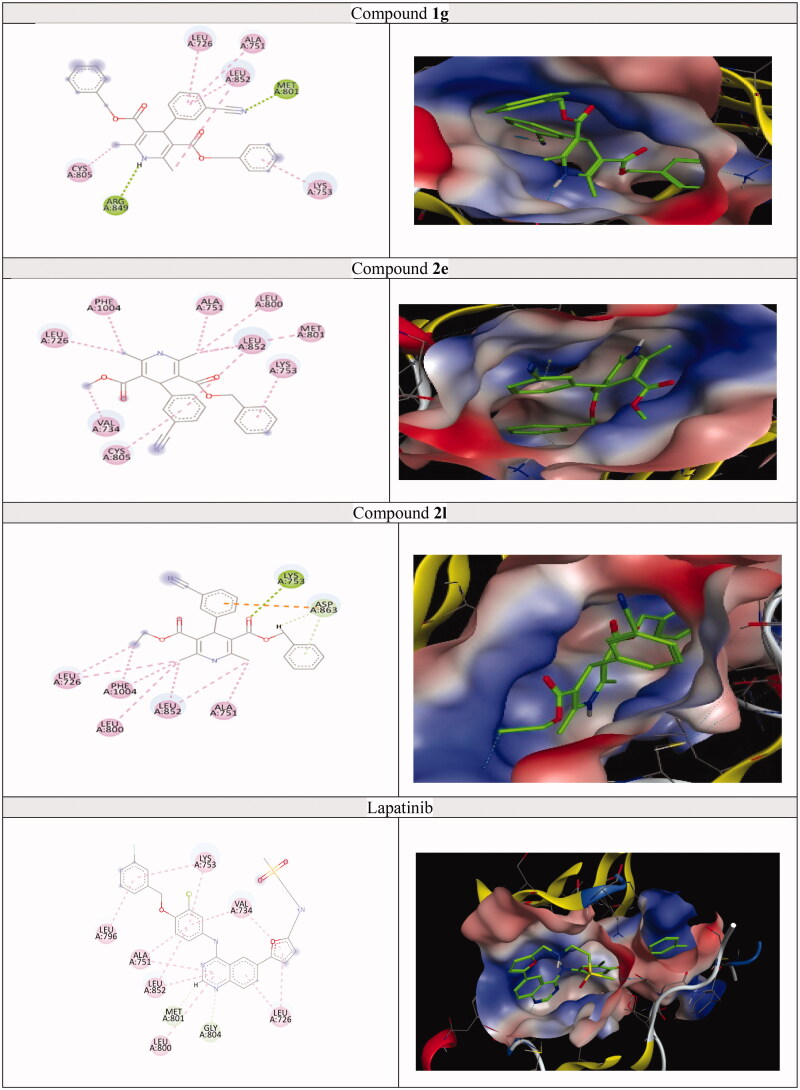
The 2D and 3D docking poses of compounds **1g**, **2e**, **2l** and lapatinib interactions with HER-2 (PDB ID: 3RCD).

**Figure 13.**
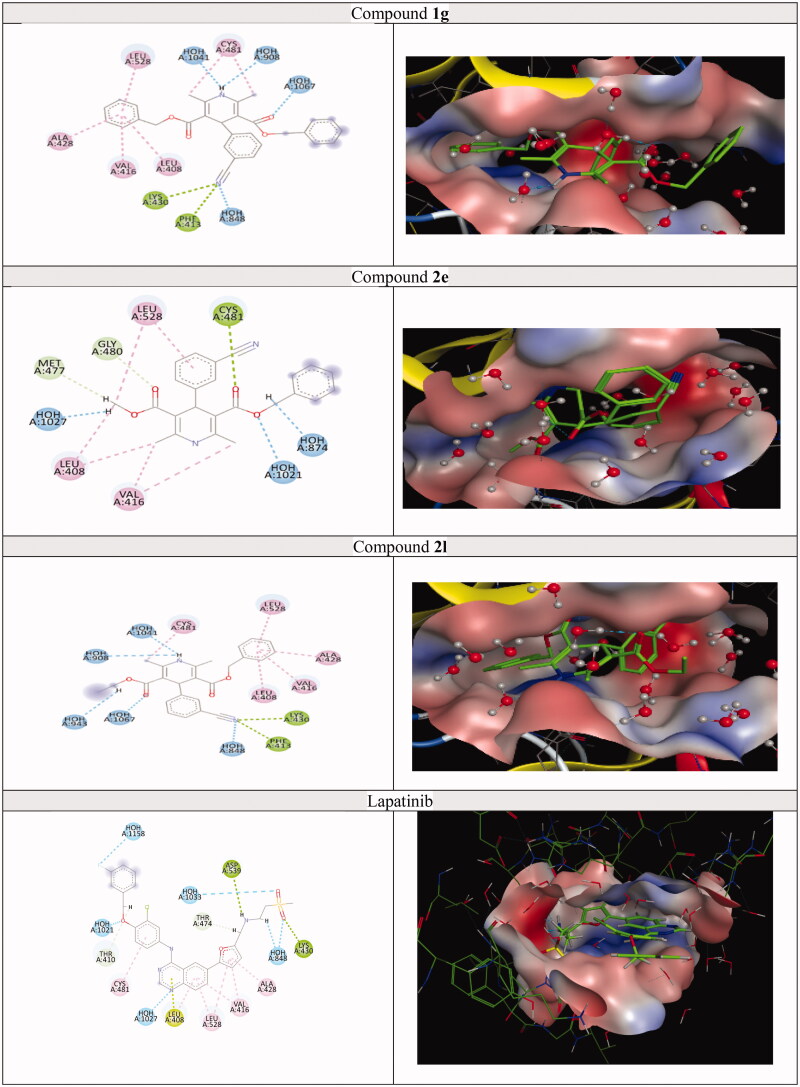
The 2D and 3D docking poses of compounds **1g**, **2e**, **2l** and lapatinib interactions with BTK (PDB ID: 4Z3V).

A molecular docking study was achieved on the active sites of targeted kinases and P-gp; P-gp (PDB ID: 3G60),^69^ EGFR (PDB ID: 1M17),^70^ HER-2 (PDB ID: 3RCD),^71^ and BTK (PDB ID: 4Z3V)^72^ and their alignment with co-crystalised ligand were shown in Figures S64–67. The docking results for each protein revealed that target compounds interacted with the values of the scoring functions, as reported in Tables 6–9 and Figures 10–13. Hydrophobic attraction forces and hydrogen bonds were accomplished by target compounds to engage with amino acid residues of the active sites.

**Table 6.** Docking outcomes for compounds **1 g**, **2e**, **2l,** and DOX in the active site of P-gp (PDB ID: 3G60).

Compound	Docking Score (kcal/mol)	π– π interaction		H-bond interaction		Hydrophobic interaction	
		Amino acid	Fragment	Amino acid	Fragment	Amino acid	Fragment
**1g**	−8.00	Phe332	Phenyl	Tyr949	C = O	Val978	Methyl (DHP)
		Phe728	Benzyl			Ala981	Benzyl Methyl (DHP)
						Val984	Benzyl
**2e**	−7.13	Phe332	Phenyl	-------	-------	Phe728	Methyl (DHP)
		Phe728	Benzyl			Val978	Phenyl (DHP)
**2l**	−7.33	Phe332	Phenyl	Tyr949	C = O	Phe974	Methyl (DHP)
		Phe728	Benzyl				
**DOX**	−8.21	Phe332	Anthraquinone ring	Tyr949	C = O	Leu64	Methoxy
		Phe728				Met67	Phenoxy
		Tyr949				Met68	Methoxy Phenoxy
						Phe332	Amino sugar
						Ile336	Methoxy
						Phe339	Amino sugar
						Phe974	Anthra-quinone
						Val978	Anthra-quinone

**Table 7.** Docking outcomes for compounds **1 g**, **2e**, **2 l,** and lapatinib in the active site of EGFR (PDB ID: 1M17).

Compound	Docking Score (kcal/mol)	π–π interaction		H-bond interaction		Hydrophobic interaction	
		Amino acid	Fragment	Amino acid	Fragment	Amino acid	Fragment
**1g**	−8.59	Met742	Benzyl	Cys773	C = O	Leu694	Methyl (DHP)
		Gly772	Benzyl			Val702	Phenyl
		Cys773	Benzyl			Ala719	Methyl (DHP) Benzyl
						Lys721	Benzyl
						Leu820	Methyl (DHP)
**2e**	−8.32	Met742	Benzyl	--------	--------	Val702	Methyl (DHP)
		Asp831	Phenyl			Ala719	Methyl (DHP) Benzyl
						Lys721	Benzyl
						Leu764	Benzyl
						Cys773	Methyl (Ester)
						Leu820	Methyl (DHP)
**2l**	−8.45	Met742	Benzyl	Met769	N-H	Leu694	Methyl (DHP)
						Val702	Phenyl
						Ala719	Methyl (DHP) Benzyl
						Lys721	Benzyl
						Leu768	Methyl (DHP)
						Met769	Methyl (DHP)
						Cys773	Phenyl
						Leu820	Methyl (DHP)
Lapatinib	−8.71	-----	-----	His781	SO_2_	Leu694	Quinazoline Phenoxy
						Ala719	Phenoxy
						Lys721	Phenyl
						Met742	Phenyl
						Leu768	Quinazoline
						Phe771	SO_2_
						Asp776	Alkylamine
						Tyr777	SO_2_
						Glu780	Alkylamine
						His781	SO_2_
						Leu820	Phenoxy

**Table 8.** Docking results for compounds **1 g**, **2e**, **2 l**, and lapatinib in the active site of HER-2 (PDB ID: 3RCD).

Compound	Docking Score (kcal/mol)	H-bond interaction		Hydrophobic interaction	
		Amino acid	Fragment	Amino acid	Fragment
**1g**	−7.72	Met801	C≡N	Leu726	Phenyl
		Arg849	N-H	Ala751	Phenyl
				Lys753	Benzyl
				Cys805	Methyl (DHP)
				Leu852	Methyl (DHP) Phenyl
**2e**	−7.60	------	------	Leu726	Methyl (DHP)
				Ala751	Methyl (DHP)
				Val734	Methyl (Ester)
				Lys753	Benzyl
				Leu800	Methyl (DHP)
				Met801	Methyl (DHP)
				Cys805	Phenyl
				Leu852	Phenyl
				Phe1004	Methyl (DHP)
**2l**	−7.51	Lys753	C = O	Leu726	Methyl (DHP) Methyl (Ester)
				Ala751	Methyl (DHP)
				Leu800	Methyl (DHP)
				Leu852	Methyl (DHP)
				Phe1004	Methyl (DHP) Methyl (Ester)
Lapatinib	−7.68	------	------	Leu726	Quinazoline Furan
				Val734	Furan Phenoxy
				Ala751	Quinazoline Phenoxy
				Lys753	Phenyl Phenoxy
				Leu796	Phenyl
				Leu800	Quinazoline
				Leu852	Quinazoline Phenyl

**Table 9.** Docking outcomes for compounds **1 g**, **2e**, **2 l,** and lapatinib in the active site of BTK (PDB ID: 4Z3V).

Compound	Docking Score (kcal/mol)	Water-mediated H-bond		H-bond interaction		Hydrophobic interaction	
		HOH no.	Fragment	Amino acid	Fragment	Amino acid	Fragment
**1g**	−8.50	HOH848	C≡N	Phe413	C≡N	Leu408	Benzyl
		HOH908	N-H	Lys430	C≡N	Val416	Benzyl
		HOH1041	N-H			Ala428	Benzyl
		HOH1067	C=O			Cys481	Methyl (DHP)
						Leu528	Benzyl
**2e**	−8.00	HOH874	OCH_2_	Cys481	C=O	Leu408	Methyl (DHP) Methyl (Ester)
		HOH1021	OCH_2_			Val416	Methyl (DHP)
		HOH1027	OCH_2_			Leu528	Methyl (Ester) Phenyl
**2l**	−8.33	HOH848	C≡N	Phe413	C≡N	Leu408	Benzyl
		HOH908	N-H	Lys430	C≡N	Val416	Benzyl
		HOH1041	N-H			Ala428	Benzyl
		HOH1067	C=O			Cys481	Methyl (DHP)
						Leu528	Benzyl
Lapatinib	−8.42	HOH848	SO_2_	Lys430	SO_2_	Leu408	Quinazoline
		HOH1021	OCH_2_	Asp539	NH	Val416	Quinazoline Furan
		HOH1027	N=C			Ala428	Furan
		HOH1033	SO_2_			Cys481	Phenoxy
		HOH1158	F			Leu528	Quinazoline Furan

#### 3.3.1. Docking of 1g, 2e, 2l, and DOX on the active site of P-gp

Docking results exposed that compounds **1g**, **2e**, **2l**, and DOX were well located within the active site of P-gp (PDB ID: 3G60). Generally, selected analogues demonstrated π–π attraction forces; between phenyl ring and Phe332 and between benzyl group and Phe728. DOX displayed π–π attraction forces with the same two amino acids beside Tyr949. H-bonds were observed between Tyr949 and carbonyl group in compounds **1g**, **2l**, and DOX however, it was not established by **2e**. The hydrophobicity factor played an important role in binding inhibitors within the active site, where **1g** (docking score = −8.00 kcal/mol) displayed the best affinity to the active site among its analogues, where it formed hydrophobic interactions with Val978 & Ala981 (with methyl groups in DHP ring) besides Ala981 and Val984 (with phenyl ring). In addition, methyl groups in DHP of compound **2e** interacted with Phe728 and Val978, while the phenyl ring interacted with Val978. Besides, compound **2l** exposed hydrophobic interaction between Phe974 and methyl groups in the DHP ring. Finally, DOX (docking score = −8.21 kcal/mol) showed hydrophobic interactions with Leu64, Met67, Met68, Ile336, Phe974, and Val978 (with anthraquinone ring and its substituents) and Phe332 and Phe339 (with sugar moiety) (Figure 10 and Table 6).

#### 3.3.2. Docking of analogues 1g, 2e, 2l, and lapatinib on the active site of EGFR

By inspecting the binding mode of compounds **1g, 2e**, and **2l** to EGFR (docking scores = −8.59, −8.32, and −8.45 kcal/mol, respectively), a remarkable network of hydrophobic interactions was set up around the phenyl ring, benzyl and methyl groups through 5–8 amino acids. Besides, both compounds **1g** and **2l** formed H-bonds with Cys773 and Met769, respectively. The benzyl group of **1g**, **2e**, and **2l** demonstrated π–π interaction with Met742. In addition, there were extra π–π interactions observed for compound **1g** through benzyl group with Gly772 and Cys773 and in compound **2e** through phenyl group with Asp831, as declared in (Figure 11 and Table 7). In comparison to lapatinib (docking scores = −8.71 kcal/mol), it did not undergo π–π interaction, but it still makes hydrogen bond with His781 and hydrophobic interaction with about eleven amino acids which enabled it to outperform the other compounds.

#### 3.3.3. Docking study for analogues 1g, 2e, 2l, and lapatinib on the active site of HER-2

HER-2 is overexpressed in various human malignancies, including not less than 40% of solid tumours, while its overexpression is linked to a bad prognosis. HER-2 is also an appealing target for tumour-specific therapy because it is expressed at low concentrations in normal human tissues. The normal kinase bilobed folding is adopted by HER-2.^73^ When compounds **1g** and **2l** interacted with HER-2 (PDB: 3RCD), two H-bonds were observed with Met801 and Arg849 in **1g** and one H-bond with Lys753 in **2l**. But compound **2e** and lapatinib didn’t interact with this target by any H-bond. Hydrophobicity of target compounds played an important role in increasing binding affinity and docking scores of **1g**, **2e**, **2l,** and lapatinib (docking scores = −7.72, −7.60, −7.51, and −7.68 kcal/mol, respectively), as presented in Table 8 and Figure 12.

#### 3.3.4. Docking of analogues 1g, 2e, 2l, and lapatinib in the active site of BTK

Several BTK mutations have been identified, which reveal the significance of specific amino acid residues in the ATP binding region (PDB: 4z3v).^74^^,^^75^ The best-suited synthesised compounds, **1g** (docking score = −8.50 kcal/mol) and **2l** (docking score = −8.33 kcal/mol), were docked into the active site of BTK, where the benzyl group fitted in the hydrophobic pocket and generated network of hydrophobic attraction forces with Leu408, Val416, Ala428, and Leu528. Similarly, Cys481 sorted hydrophobic interaction with two methyl groups at C2 and C6 of compound **1g** while making hydrophobic interaction with only one methyl group of compound **2l**. Nitrile group at **1g** and **2l** interact with Phe413 and Lys430 *via* two H-bonds. Finally, both compounds make four water-mediated H-bonds with HOH848, HOH908, HOH1041, and HOH1067, as displayed in Figure 13 and Table 9. Meanwhile, compound **2e** (docking score = −8.0 kcal/mol) displayed hydrophobic interaction through phenyl and methyl groups with Leu408, Val416, and Leu528. **2e** formed one H-bond through carbonyl group with Cys481 besides three water-mediated H-bonds with HOH874, HOH1021, and HOH1027, as shown in Figure 13 and Table 9. Finally, lapatinib (docking score = −8.42 kcal/mol) displayed similar binding interactions to compound **1g** (H-bond and hydrophobic) towards BTK.

## 4. Conclusion

Two new series comprised eight symmetric achiral (**1a-h)** and twelve asymmetric chiral (**2a-l)** 1,4-DHP derivatives were designed and synthesised. Chemical structures of target compounds were confirmed by elemental analysis, ^1^H NMR, ^13^C NMR, and mass spectroscopy. Screening of cytotoxic effect of target compounds against NCI sixty cell lines of nine different types of human tumour revealed that compounds **1g**, **2e**, and **2l** reported the best activities. EGFR, HER-2, and BTK inhibitory effects of compounds **1g**, **2e**, and **2l** were close to that of lapatinib. Annexin V–FITC apoptosis assay exposed that analogues **1g**, **2e**, and **2l** attained antiproliferative activity through apoptotic mechanism rather than a necrotic pathway. DNA flow cytometric analysis displayed that, for analogues **1g**, **2e**, and **2l,** the proportion of apoptotic cells is significantly increased at the G2/M phase; thus, they have arrested cells at the G2/M phase. Evaluation of the antimicrobial activity of the synthesised compounds revealed that compound **1g** has a remarkable effect as anticancer and antimicrobial together. Moreover, although being the greatest anticancer among the series, compound **2l** has no effect on microbial infection. Docking studies for selected compounds were performed in the active sites of crystal structures of four proteins to understand biological differences.

## 5. Experimental

### 5.1. Chemistry

All of the organic reagents used in this study were purchased from Sigma-Aldrich, Alfa Aesar, and Merck, and were utilised without additional purification. To improve the photo-stability of the products, they were prepared, collected, and purified in the dark in the absence of oxidising agents. Thin-layer chromatography (TLC) was utilised to monitor reaction courses, product mixes, and purity, utilising a pre-coated sheet with a Fluorescein indicator (Fastman Kodak Co., Silica 60 F_254_) and shortwave UV light at 254 nm. A PuriFlash 4100 system (Interchim; Montlucon, France) was utilised to separate compound **2c** from its product combination using preparative silica gel HPLC. The system includes a mixing HPLC quaternary pump, PDA-UV–Vis detector (190–840nm), fraction collector, and sample loading module. For system control and process monitoring, Interchim Software version 5.0 was used. The column utilised was a 25g normal phase (NP) column with a particle size of 30 m silica. The **2c** product combination was diluted in 100 mL of *n*-hexane: ethyl acetate (1:1) before being dry loaded into the column with 12g of silica. To improve separation and minimise separation time, HPLC chromatography was performed in elution gradient mode, with the mobile phase composition varying from 0% to 100% v/v ethyl acetate - *n*-hexane during separation.

Compounds **2a-l** had their optical rotation (α) determined on a Polax-2L Polarimeter (ATAGO Co., Ltd., Japan) at Tanta University’s Department of Pharmaceutical Chemistry, Faculty of Pharmacy, and there was no rotation. The open capillary method was utilised to measure melting points, which were not adjusted, using electro-thermal equipment (Stuart SMP10). Micro Analytical Centre, Faculty of Science, Cairo University, Giza, Egypt, used a Perkin-Elmer 2400 CHN analyser to do elemental analysis (percent C, H, and N), and all elemental analysis data were within ±0.4 of theoretical values. Using CDCl_3_ as a solvent, ^1^H NMR spectra were acquired at 400 MHz on a Bruker FT-NMR spectrometer. Using CDCl_3_ as a solvent, ^13^C NMR spectra were acquired at 125 MHz on a JEOL ECA-500 II FT-NMR spectrometer. Chemical shifts were measured in parts per million (ppm) compared to the internal reference, tetramethylsilane (TMS). The coupling constants were expressed in hertz (Hz). Faculty of Science, Kafrelsheikh University, Egypt, conducted ^1^H NMR spectra. Faculty of Science, Mansoura University, Egypt, conducted ^13^C NMR spectra. In negative ion mode, electrospray ionisation mass spectra (ESI–MS) were acquired on an Advion compact mass spectrometer (CMS). Nawah Scientific Co., Al-Asmarat, Almokattam, Cairo, Egypt, performed the ESI–MS.

#### 5.1.1. General procedure for the synthesis of symmetric compounds (1a-h)

Ammonium acetate (0.338g, 4.385 mmol) was added to a stirring solution of 3-cyanobenzaldehyde (0.500g, 3.813 mmol), and the corresponding alkyl acetoacetate (7.626 mmol) in methanol (10 mL) in a 50 mL round-bottomed flask. The reaction mixture was kept in the dark and heated under reflux for 24h. The cooled solution was stored in the refrigerator. To obtain products (**1a-h**), the crude crystals were filtered and refined *via* crystallisation from methanol.

##### 5.1.1.1. Dimethyl 4-(3-cyanophenyl)-2,6-dimethyl-1,4-dihydropyridine-3,5-dicarboxylate (1a)

Yellowish white powder, m.p. 200–202 °C.^76^ ^1^H NMR (400 MHz, CDCl_3_) δ (ppm): 2.29 (s, 6H, C_2_-**CH_3_** and C_6_-**CH_3_**), 3.58 (s, 6H, 2 of COO**CH_3_**), 4.95 (s, 1H, C_4_-**H**), 5.73 (s, 1H, N**H**), 7.24 (t, *J* = 7.70 Hz, 1H, C_5’_-**H**), 7.36 (d, *J* = 7.70 Hz, 1H, C_4’_-**H**), 7.46 (d, *J* = 7.70 Hz, 1H, C_6’_-**H**), 7.47 (s, 1H, C_2’_-**H**). Anal. (%) for C_18_H_18_N_2_O_4_, calcd. (found), C 66.25 (66.03), H 5.56 (5.52), N 8.58 (8.61).

##### 5.1.1.2. Diethyl 4-(3-cyanophenyl)-2,6-dimethyl-1,4-dihydropyridine-3,5-dicarboxylate (1b)

Yellow powder, m.p. 144–146 °C.^76^ ^1^H NMR (400 MHz, CDCl_3_) δ (ppm): 1.24 (t, *J* *=* 7.08 Hz, 6H, 2 of CH_2_**CH_3_**), 2.37 (s, 6H, C_2_-**CH_3_** and C_6_-**CH_3_**), 4.05–4.17 (m, 4H, 2 of **CH_2_**CH_3_), 5.03 (s, 1H, C_4_-**H**), 5.90 (s, 1H, N**H**), 7.32 (t, *J* = 7.60 Hz, 1H, C_5’_-**H**), 7.44 (d, *J* = 7.60 Hz, 1H, C_4’_-**H**), 7.57 (d, *J* = 7.60 Hz, 1H, C_6’_-**H**), 7.58 (s, 1H, C_2’_-**H**). Anal. (%) for C_20_H_22_N_2_O_4_, calcd. (found), C 67.78 (67.51), H 6.26 (6.14), N 7.90 (8.00).

##### 5.1.1.3. Diisopropyl 4-(3-cyanophenyl)-2,6-dimethyl-1,4-dihydropyridine-3,5-dicarboxylate (1c)

Yield (0.400g, 27%) as yellowish white powder, m.p. 124–125 °C. ^1^H NMR (400 MHz, CDCl_3_) δ (ppm): 1.04 (d, *J* = 6.24 Hz, 6H, 2 of **CH_3_**CHCH_3_), 1.17 (d, *J* = 6.24 Hz, 6H, 2 of CH_3_CH**CH_3_**), 2.26 (s, 6H, C_2_-**CH_3_** and C_6_-**CH_3_**), 4.83–4.93 (m, 3H, 2 of COO**CH** and C_4_-**H**), 5.81 (s, 1H, N**H**), 7.23 (t, *J* = 7.62 Hz, 1H, C_5’_-**H**), 7.35 (d, *J* = 7.62 Hz, 1H, C_4’_-**H**), 7.48 (d, *J* = 7.62 Hz, 1H, C_6’_-**H**), 7.49 (s, 1H, C_2’_-**H**). ESI–MS: *m/z* (% abundance): 382.6 (M^+^, 25), 381.6 ([M – 1]^+^, 100). Anal. (%) for C_22_H_26_N_2_O_4_, calcd. (found), C 69.09 (68.86), H 6.85 (6.89), N 7.32 (7.08).

##### 5.1.1.4. Diisobutyl 4-(3-cyanophenyl)-2,6-dimethyl-1,4-dihydropyridine-3,5-dicarboxylate (1d)

Yield (1.213g, 78%) as yellow powder, m.p. 146–148 °C. ^1^H NMR (400 MHz, CDCl_3_) δ (ppm): 0.90 (d, *J* = 6.78 Hz, 6H, 2 of **CH_3_**CHCH_3_), 0.92 (d, *J* = 6.78 Hz, 6H, 2 of CH_3_CH**CH_3_**), 1.87–1.97 (m, 2H, 2 of CH_2_**CH**), 2.39 (s, 6H, C_2_-**CH_3_** and C_6_-**CH_3_**), 3.86 (d, *J* = 6.48 Hz, 4H, 2 of COO**CH_2_**), 5.09 (s, 1H, C_4_-**H**), 5.83 (s, 1H, N**H**), 7.33 (t, *J* = 7.72 Hz, 1H, C_5’_-**H**), 7.45 (d, *J* = 7.72 Hz, 1H, C_4’_-**H**), 7.58 (d, *J* = 7.72 Hz, 1H, C_6’_-**H**), 7.59 (s, 1H, C_2’_-**H**). ESI–MS: *m/z* (% abundance): 410.7 (M^+^, 28), 409.7 ([M – 1]^+^, 100). Anal. (%) for C_24_H_30_N_2_O_4_, calcd. (found), C 70.22 (69.92), H 7.37 (6.99), N 6.82 (6.52).

##### 5.1.1.5. Di-*tert*-butyl 4-(3-cyanophenyl)-2,6-dimethyl-1,4-dihydropyridine-3,5-dicarboxylate (1e)

Yield (0.782g, 50%) as white powder, m.p. 166–168 °C. ^1^H NMR (400 MHz, CDCl_3_) δ (ppm): 1.41 (s, 18H, 2 of C**(CH_3_)_3_**), 2.32 (s, 6H, C_2_-**CH_3_** and C_6_-**CH_3_**), 4.95 (s, 1H, C_4_-**H**), 5.78 (s, 1H, N**H**), 7.33 (t, *J* = 7.18 Hz, 1H, C_5’_-**H**), 7.44 (d, *J* = 7.18 Hz, 1H, C_4’_-**H**), 7.57 (d, *J* = 7.18 Hz, 1H, C_6’_-**H**), 7.58 (s, 1H, C_2’_-**H**). ESI–MS: *m/z* (% abundance): 410.7 (M^+^, 27), 409.7 ([M – 1]^+^, 100). Anal. (%) for C_24_H_30_N_2_O_4_, calcd. (found), C 70.22 (70.23), H 7.37 (7.72), N 6.82 (7.21).

##### 5.1.1.6. Di(2-methoxyethyl) 4-(3-cyanophenyl)-2,6-dimethyl-1,4-dihydropyridine-3,5-dicarboxylate (1f)

Yield (0.589g, 37%) as white powder, m.p. 125–126 °C. ^1^H NMR (400 MHz, CDCl_3_) δ (ppm): 2.28 (s, 6H, C_2_-**CH_3_** and C_6_-**CH_3_**), 3.30 (s, 6H, 2 of O**CH_3_**), 3.41–3.52 (m, 4H, 2 of **CH_2_**OCH_3_), 4.04–4.17 (m, 4H, 2 of COO**CH_2_**), 4.98 (s, 1H, C_4_-**H**), 5.93 (s, 1H, N**H**), 7.24 (t, *J* = 7.61 Hz, 1H, C_5’_-**H**), 7.35 (d, *J* = 7.61 Hz, 1H, C_4’_-**H**), 7.51 (d, *J* = 7.61 Hz, 1H, C_6’_-**H**), 7.54 (s, 1H, C_2’_-**H**). ^13^C NMR (125 MHz, CDCl_3_) δ (ppm): 19.5 (2 C, C_2_-**CH_3_** and C_6_-**CH_3_**), 39.7 (1 C, C_4_), 58.8 (2 C, 2 of O**CH_3_**), 62.9 (2 C, 2 of **CH_2_**OCH_3_), 70.4 (2 C, 2 of COO**CH_2_**), 103.1 (2 C, C_3_ and C_5_), 111.6 (1 C, C_3’_), 119.5 (1 C, CN), 128.5 (1 C, C_5’_), 129.8 (1 C, C_4’_), 132.1 (1 C, C_6’_), 132.9 (1 C, C_2’_), 144.9 (2 C, C_2_ and C_6_), 149.0 (1 C, C_1’_), 167.2 (2 C, COO). ESI–MS: *m/z* (% abundance): 414.6 (M^+^, 27), 413.6 ([M – 1]^+^, 100). Anal. (%) for C_22_H_26_N_2_O_6_, calcd. (found), C 63.76 (63.68), H 6.32 (6.01), N 6.76 (6.81).

##### 5.1.1.7. Dibenzyl 4-(3-cyanophenyl)-2,6-dimethyl-1,4-dihydropyridine-3,5-dicarboxylate (1g)

Yield (0.884g, 48%) as yellow powder, m.p. 162–164 °C. ^1^H NMR (400 MHz, CDCl_3_) δ (ppm): 2.27 (s, 6H, C_2_-**CH_3_** and C_6_-**CH_3_**), 4.93 (d, *J*_gem_ = 12.29 Hz, 2H, 2 of **H**CHC_6_H_5_), 4.95 (s, 1H, C_4_-**H**), 5.02 (d, *J*_gem_ = 12.29 Hz, 2H, 2 of HC**H**C_6_H_5_), 5.75 (s, 1H, N**H**), 7.07–7.34 (m, 14H, 2 of **C_6_H_5_**, C_2’_-**H**, C_4’_-**H**, C_5’_-**H** and C_6’_-**H**). ^13^C NMR (125 MHz, CDCl_3_) δ (ppm): 19.7 (2 C, C_2_-**CH_3_** and C_6_-**CH_3_**), 39.6 (1 C, C_4_), 65.9 (2 C, 2 of **CH_2_**C_6_H_5_), 103.1 (2 C, C_3_, and C_5_), 111.7 (1 C, C_3’_), 119.3 (1 C, CN), 128.1 (4 C, 2 of (C_2_ and C_6_ of C_6_H_5_)), 128.5 (6 C, 2 of (C_3_, C_4_^,^ and C_5_ of C_6_H_5_)), 128.5 (1 C, C_5’_), 129.9 (1 C, C_4’_), 131.8 (1 C, C_6’_), 132.9 (1 C, C_2’_), 136.0 (2 C, 2 of (C_1_ of C_6_H_5_)), 145.0 (2 C, C_2_, and C_6_), 148.8 (1 C, C_1’_), 166.7 (2 C, COO). ESI–MS: *m/z* (% abundance): 478.8 (M^+^, 36), 477.8 ([M – 1]^+^, 100). Anal. (%) for C_30_H_26_N_2_O_4_, calcd. (found), C 75.30 (74.96), H 5.48 (5.18), N 5.85 (5.61).

##### 5.1.1.8. Diethyl 4-(3-cyanophenyl)-2,6-dipropyl-1,4-dihydropyridine-3,5-dicarboxylate (1h)

Yield (0.633g, 40%) as white powder, m.p. 140–141 °C. ^1^H NMR (400 MHz, CDCl_3_) δ (ppm): 1.02 (t, *J* = 7.30, 6H, 2 of (CH_2_)_2_**CH_3_**), 1.25 (t, *J* = 7.10, 6H, 2 of COOCH_2_**CH_3_**), 1.56–1.75 (m, 4H, 2 of CH_2_**CH_2_**CH_3_), 2.57–2.64 (m, 2H, 2 of **H**CHCH_2_CH_3_), 2.80–2.88 (m, 2H, 2 of HC**H**CH_2_CH_3_), 4.06–4.18 (m, 4H, 2 of COO**CH_2_**), 5.05 (s, 1H, C_4_-**H**), 5.77 (s, 1H, N**H**), 7.33 (t, *J*  = 7.58 Hz, 1H, C_5’_-**H**), 7.44 (d, *J* = 7.58 Hz, 1H, C_4’_-**H**), 7.57 (d, *J* = 7.58 Hz, 1H, C_6’_-**H**), 7.58 (s, 1H, C_2’_-**H**). ^13^C NMR (125 MHz, CDCl_3_) δ (ppm): 13.9 (2 C, 2 of (CH_2_)_2_**CH_3_**), 14.2 (2 C, 2 of COOCH_2_**CH_3_**), 21.9 (2 C, 2 of CH_2_**CH_2_**CH_3_), 34.6 (2 C, 2 of **CH_2_**CH_2_CH_3_), 39.8 (1 C, C_4_), 59.9 (2 C, 2 of COO**CH_2_**), 102.9 (2 C, C_3_, and C_5_), 111.7 (1 C, C_3’_), 119.4 (1 C, CN), 128.5 (1 C, C_5’_), 129.8 (1 C, C_4’_), 131.8 (1 C, C_6’_), 132.7 (1 C, C_2’_), 148.8 (2 C, C_2_, and C_6_), 149.2 (1 C, C_1’_), 166.7 (2 C, COO). ESI–MS: *m/z* (% abundance): 410.8 (M^+^, 27), 409.8 ([M – 1]^+^, 100). Anal. (%) for C_24_H_30_N_2_O_4_, calcd. (found), C 70.22 (70.48), H 7.37 (7.42), N 6.82 (6.61).

#### 5.1.2. General procedure for the synthesis of asymmetric compounds (2a-l)

A mixture of 3-cyanobenzaldehyde (0.500g, 3.813 mmol), respective alkyl acetoacetate (3.813 mmol), and respective alkyl 3-aminocrotonates (3.813 mmol) in methanol (10 mL) was added to a 50 mL round-bottomed flask. The reaction mixture was kept away from light and heated for 24h under reflux with stirring. The cooled solution was stored in the refrigerator. To obtain products (**2a-l**), the crude crystals were filtered and purified by crystallisation from methanol.

##### 5.1.2.1. (±)-3-Isopropyl 5-methyl 4-(3-cyanophenyl)-2,6-dimethyl-1,4-dihydropyridine-3,5-dicarboxylate (2a)

Yield (0.679g, 50%) as white powder, m.p. 128–130 °C. ^1^H NMR (400 MHz, CDCl_3_) δ (ppm): 1.13 (d, *J* =  6.20 Hz, 3H, **CH_3_**CHCH_3_), 1.24 (d, *J* = 6.20 Hz, 3H, CH_3_CH**CH_3_**), 2.37 (s, 6H, C_2_-**CH_3_,** and C_6_-**CH_3_**), 3.66 (s, 3H, O**CH_3_**), 4.95–5.01 (m, 2H, C_4_-**H** and **CH**(CH_3_)_2_), 5.81 (s, 1H, N**H**), 7.33 (t, *J* = 7.59 Hz, 1H, C_5’_-**H**), 7.44 (d, *J* = 7.59 Hz, 1H, C_4’_-**H**), 7.56 (d, *J* = 7.59  Hz, 1H, C_6’_-**H**), 7.57 (s, 1H, C_2’_-**H**). ESI–MS: *m/z* (% abundance): 354.6 (M^+^, 23), 353.6 ([M – 1]^+^, 100). Anal. (%) for C_20_H_22_N_2_O_4_, calcd. (found), C 67.78 (67.92), H 6.26 (5.98), N 7.90 (7.92).

##### 5.1.2.2. (±)-3-Isobutyl 5-methyl 4-(3-cyanophenyl)-2,6-dimethyl-1,4-dihydropyridine-3,5-dicarboxylate (2b)

Yield (0.853g, 61%) as white powder, m.p. 133–135 °C. ^1^H NMR (400 MHz, CDCl_3_) δ (ppm): 0.86 (d, *J* = 6.72 Hz, 3H, **CH_3_**CHCH_3_), 0.89 (d, *J* = 6.72 Hz, 3H, CH_3_CH**CH_3_**), 1.86–1.96 (m, 1H, **CH**(CH_3_)_2_), 2.36 (s, 3H, C_6_-**CH_3_**), 2.39 (s, 3H, C_2_-**CH_3_**), 3.68 (s, 3H, O**CH_3_**), 3.79–3.91 (m, 2H, COO**CH_2_**), 5.05 (s, 1H, C_4_-**H**), 5.93 (s, 1H, N**H**), 7.33 (t, *J* = 7.76 Hz, 1H, C_5’_-**H**), 7.45 (d, *J* = 7.76 Hz, 1H, C_4’_-**H**), 7.53–7.63 (m, 2H, C_2’_-**H** and C_6’_-**H**). ^13^C NMR (125 MHz, CDCl_3_) δ (ppm): 19.7 (2 C, 2 of CH**(CH_3_)_2_**, 19.7 (2 C, C_2_-**CH_3_,** and C_6_-**CH_3_**), 27.7 (1 C, **CH**(CH_3_)_2_), 39.5 (1 C, C_4_), 51.1 (1 C, O**CH_3_**), 70.4 (1 C, COO**CH_2_**), 103.2 (1 C, C_5_), 103.3 (1 C, C_3_), 111.9 (1 C, C_3’_), 119.4 (1 C, CN), 128.6 (1 C, C_5’_), 129.9 (1 C, C_4’_), 131.6 (1 C, C_6’_), 132.6 (1 C, C_2’_), 144.6 (1 C, C_6_), 144.7 (1 C, C_2_), 148.9 (1 C, C_1’_), 167.1 (1 C, C_3_-**COO**), 167.6 (1 C, C_5_-**COO**). ESI–MS: *m/z* (% abundance): 368.6 (M^+^, 22), 367.6 ([M – 1]^+^, 100). Anal. (%) for C_21_H_24_N_2_O_4_, calcd. (found), C 68.46 (68.64), H 6.57 (6.39), N 7.60 (7.55).

##### 5.1.2.3. (±)-3-*Tert*-butyl 5-Methyl 4-(3-cyanophenyl)-2,6-dimethyl-1,4-dihydropyridine-3,5-dicarboxylate (2c)

Yield (0.579g, 41%) as white powder, m.p. 157–159 °C. ^1^H NMR (400 MHz, CDCl_3_) δ (ppm): 1.32 (s, 9H, C(**CH_3_**)_3_), 2.25 (s, 3H, C_6_-**CH_3_**), 2.26 (s, 3H, C_2_-**CH_3_**), 3.57 (s, 3H, O**CH_3_**), 4.88 (s, 1H, C_4_-**H**), 5.59 (s, 1H, N**H**), 7.24 (t, *J* = 7.67 Hz, 1H, C_5’_-**H**), 7.35 (d, *J* = 7.67 Hz, 1H, C_4’_-**H**), 7.47 (d, *J* = 7.67 Hz, 1H, C_6’_-**H**), 7.48 (s, 1H, C_2’_-**H**). ESI–MS: *m/z* (% abundance): 368.7 (M^+^, 25), 367.7 ([M – 1]^+^, 100). Anal. (%) for C_21_H_24_N_2_O_4_, calcd. (found), C 68.46 (68.23), H 6.57 (6.87), N 7.60 (7.21).

##### 5.1.2.4. (±)-3-(2-Methoxyethyl) 5-methyl 4-(3-cyanophenyl)-2,6-dimethyl-1,4-dihydropyridine-3,5-dicarboxylate (2d)

Yield (1.045g, 74%) as white powder, m.p. 127–128 °C. ^1^H NMR (400 MHz, CDCl_3_) δ (ppm): 2.37 (s, 3H, C_6_-**CH_3_**), 2.38 (s, 3H, C_2_-**CH_3_**), 3.39 (s, 3H, CH_2_O**CH_3_**), 3.51–3.62 (m, 2H, **CH_2_**OCH_3_), 3.66 (s, 3H, COO**CH_3_**), 4.14–4.19 (m, 1H, **H**CHCH_2_OCH_3_), 4.23–4.29 (m, 1H, HC**H**CH_2_OCH_3_), 5.05 (s, 1H, C_4_-**H**), 5.90 (s, 1H, N**H**), 7.33 (t, *J* = 7.56 Hz, 1H, C_5’_-**H**), 7.45 (d, *J* = 7.56 Hz, 1H, C_4’_-**H**), 7.58 (d, *J* = 7.56 Hz, 1H, C_6’_-**H**), 7.59 (s, 1H, C_2’_-**H**). ESI–MS: *m/z* (% abundance): 370.6 (M^+^, 22), 369.6 ([M – 1]^+^, 100). Anal. (%) for C_20_H_22_N_2_O_5_, calcd. (found), C 64.85 (64.86), H 5.99 (6.11), N 7.56 (7.27).

##### 5.1.2.5. (±)-3-Benzyl 5-methyl 4-(3-cyanophenyl)-2,6-dimethyl-1,4-dihydropyridine-3,5-dicarboxylate (2e)

Yield (1.211g, 79%) as white powder, m.p. 129–131 °C. ^1^H NMR (400 MHz, CDCl_3_) δ (ppm): 2.36 (s, 3H, C_6_-**CH_3_**), 2.38 (s, 3H, C_2_-**CH_3_**), 3.65 (s, 3H, O**CH_3_**), 5.03 (d, *J*_gem_ = 12.13 Hz, 1H, **H**CHC_6_H_5_), 5.05 (s, 1H, C_4_-**H**), 5.17 (d, *J*_gem_ = 12.13 Hz, 1H, HC**H**C_6_H_5_), 6.10 (s, 1H, N**H**), 7.24–7.56 (m, 9H, **C_6_H_5_**, C_2’_-**H**, C_4’_-**H**, C_5’_-**H** and C_6’_-**H**). ESI–MS: *m/z* (% abundance): 402.6 (M^+^, 27), 401.6 ([M – 1]^+^, 100). Anal. (%) for C_24_H_22_N_2_O_4_, calcd. (found), C 71.63 (71.91), H 5.51 (5.80), N 6.96 (6.67).

##### 5.1.2.6. (±)-3-Ethyl 5-methyl 4-(3-cyanophenyl)-6-methyl-2-propyl-1,4-dihydropyridine-3,5-dicarboxylate (2f)

Yield (0.223g, 16%) as white powder, m.p. 121–122 °C. ^1^H NMR (400 MHz, CDCl_3_) δ (ppm): 1.02 (t, *J*  = 7.16, 3H, (CH_2_)_2_**CH_3_**), 1.25 (t, *J* = 6.96, 3H, COOCH_2_**CH_3_**), 1.59–1.75 (m, 2H, CH_2_**CH_2_**CH_3_), 2.38 (s, 3H, C_6_-**CH_3_**), 2.64–2.80 (m, 2H, **CH_2_**CH_2_CH_3_), 3.67 (s, 3H, COO**CH_3_**), 4.06–4.18 (m, 2H, COO**CH_2_**), 5.05 (s, 1H, C_4_-**H**), 5.78 (s, 1H, N**H**), 7.33 (t, *J* = 7.53 Hz, 1H, C_5’_-**H**), 7.45 (d, *J* = 7.53 Hz, 1H, C_4’_-**H**), 7.56 (d, *J*  = 7.53 Hz, 1H, C_6’_-**H**), 7.57 (s, 1H, C_2’_-**H**). ESI–MS: *m/z* (% abundance): 368.7 (M^+^, 25), 367.7 ([M – 1]^+^, 100). Anal. (%) for C_21_H_24_N_2_O_4_, calcd. (found), C 68.46 (68.50), H 6.57 (6.39), N 7.60 (7.33).

##### 5.1.2.7. (±)-3-Ethyl 5-methyl 4-(3-cyanophenyl)-2,6-dimethyl-1,4-dihydropyridine-3,5-dicarboxylate (2g)

Yield (0.557g, 43%) as white powder, m.p. 151–153 °C. ^1^H NMR (400 MHz, CDCl_3_) δ (ppm): 1.24 (t, *J* = 7.10 Hz, 3H, CH_2_**CH_3_**), 2.37 (s, 6H, C_2_-**CH_3_** and C_6_-**CH_3_**), 3.67 (s, 3H, COO**CH_3_**), 4.07–4.16 (m, 2H, COO**CH_2_**), 5.03 (s, 1H, C_4_-**H**), 5.85 (s, 1H, N**H**), 7.33 (t, *J* = 7.73 Hz, 1H, C_5’_-**H**), 7.45 (d, *J* = 7.73 Hz, 1H, C_4’_-**H**), 7.56 (d, *J* = 7.73 Hz, 1H, C_6’_-**H**), 7.57 (s, 1H, C_2’_-**H**). ESI–MS: *m/z* (% abundance): 340.5 (M^+^, 19), 339.5 ([M – 1]^+^, 100). Anal. (%) for C_19_H_20_N_2_O_4_, calcd. (found), C 67.05 (66.81), H 5.92 (6.15), N 8.23 (8.04).

##### 5.1.2.8. (±)-3-Ethyl 5-isopropyl 4-(3-cyanophenyl)-2,6-dimethyl-1,4-dihydropyridine-3,5-dicarboxylate (2h)

Yield (0.361g, 26%) as yellow powder, m.p. 136–138 °C. ^1^H NMR (400 MHz, CDCl_3_) δ (ppm): 1.13 (d, *J* = 6.12 Hz, 3H, **CH_3_**CHCH_3_), 1.24 (t, *J* = 7.04 Hz, 3H, CH_2_**CH_3_**), 1.27 (d, *J* = 6.12 Hz, 3H, CH_3_CH**CH_3_**), 2.37 (s, 6H, C_2_-**CH_3_,** and C_6_-**CH_3_**), 4.05–4.18 (m, 2H, **CH_2_**CH_3_), 4.93–5.03 (m, 2H, **CH**(CH_3_)_2_ and C_4_-**H**), 5.77 (s, 1H, N**H**), 7.32 (t, *J* = 7.56 Hz, 1H, C_5’_-**H**), 7.44 (d, *J* = 7.56 Hz, 1H, C_4’_-**H**), 7.57 (d, *J* = 7.56 Hz, 1H, C_6’_-**H**), 7.58 (s, 1H, C_2’_-**H**). ESI–MS: *m/z* (% abundance): 368.7 (M^+^, 23), 367.7 ([M – 1]^+^, 100). Anal. (%) for C_21_H_24_N_2_O_4_, calcd. (found), C 68.46 (68.57), H 6.57 (6.33), N 7.60 (7.45).

##### 5.1.2.9. (±)-3-Ethyl 5-isobutyl 4-(3-cyanophenyl)-2,6-dimethyl-1,4-dihydropyridine-3,5-dicarboxylate (2i)

Yield (0.299g, 21%) as yellow powder, m.p. 120–121 °C. ^1^H NMR (400 MHz, CDCl_3_) δ (ppm): 0.87 (d, *J* = 6.72 Hz, 3H, **CH_3_**CHCH_3_), 0.90 (d, *J* = 6.72 Hz, 3H, CH_3_CH**CH_3_**), 1.26 (t, *J* = 7.16 Hz, 3H, CH_2_**CH_3_**), 1.86–1.96 (m, 1H, **CH**(CH_3_)_2_, 2.37 (s, 3H, C_2_-**CH_3_**), 2.40 (s, 3H, C_6_-**CH_3_**), 3.80–3.90 (m, 2H, COO**CH_2_**CH_3_), 4.07–4.17 (m, 2H, COO**CH_2_**CH), 5.06 (s, 1H, C_4_-**H**), 5.85 (s, 1H, N**H**), 7.33 (t, *J* = 7.79 Hz, 1H, C_5’_-**H**), 7.45 (d, *J* = 7.79 Hz, 1H, C_4’_-**H**), 7.58 (d, *J* = 7.79 Hz, 1H, C_6’_-**H**), 7.59 (s, 1H, C_2’_-**H**). ESI–MS: *m/z* (% abundance): 382.7 (M^+^, 25), 381.7 ([M – 1]^+^, 100). Anal. (%) for C_22_H_26_N_2_O_4_, calcd. (found), C 69.09 (69.18), H 6.85 (7.00), N 7.32 (7.10).

##### 5.1.2.10. (±)-3-*Tert*-butyl 5-ethyl 4-(3-cyanophenyl)-2,6-dimethyl-1,4-dihydropyridine-3,5-dicarboxylate (2j)

Yield (0.623g, 43%) as yellow powder, m.p. 152–153 °C. ^1^H NMR (400 MHz, CDCl_3_) δ (ppm): 1.24 (t, *J* = 7.10 Hz, 3H, CH_2_**CH_3_**), 1.41 (s, 9H, C**(CH_3_)_3_**), 2.34 (s, 3H, C_6_-**CH_3_**), 2.36 (s, 3H, C_2_-**CH_3_**), 4.07–4.16 (m, 2H, COO**CH_2_**), 4.98 (s, 1H, C_4_-**H**), 5.65 (s, 1H, N**H**), 7.33 (t, *J* = 7.56 Hz, 1H, C_5’_-**H**), 7.45 (d, *J* = 7.56 Hz, 1H, C_4’_-**H**), 7.57 (d, *J* = 7.56 Hz, 1H, C_6’_-**H**), 7.58 (s, 1H, C_2’_-**H**). ^13^C NMR (125 MHz, CDCl_3_) δ (ppm): 14.2 (1 C, CH_2_**CH_3_**), 19.6 (1 C, C_6_-**CH_3_**), 19.7 (1 C, C_2_-**CH_3_**), 28.2 (3 C, C**(CH_3_)_3_**), 40.0 (1 C, C_4_), 59.9 (1 C, COO**CH_2_**), 80.2 (1 C, **C**(CH_3_)_3_), 103.0 (1 C, C_5_), 104.8 (1 C, C_3_), 111.7 (1 C, C_3’_), 119.4 (1 C, CN), 128.4 (1 C, C_5’_), 129.7 (1 C, C_4’_), 131.9 (1 C, C_6’_), 132.8 (1 C, C_2’_), 143.4 (1 C, C_6_), 144.5 (1 C, C_2_), 149.2 (1 C, C_1’_), 166.4 (1 C, C_3_-**COO**), 167.2 (1 C, C_5_-**COO**). ESI–MS: *m/z* (% abundance): 382.6 (M^+^, 26), 381.6 ([M – 1]^+^, 100). Anal. (%) for C_22_H_26_N_2_O_4_, calcd. (found), C 69.09 (68.96), H 6.85 (7.15), N 7.32 (7.02).

##### 5.1.2.11. (±)-3-Ethyl 5-(2-methoxyethyl) 4-(3-cyanophenyl)-2,6-dimethyl-1,4-dihydropyridine-3,5-dicarboxylate (2k)

Yield (0.629g, 43%) as yellowish white powder, m.p. 133–134 °C. ^1^H NMR (400 MHz, CDCl_3_) δ (ppm): 1.23 (t, *J* = 7.10 Hz, 3H, CH_2_**CH_3_**), 2.37 (s, 3H, C_2_-**CH_3_**), 2.38 (s, 3H, C_6_-**CH_3_**), 3.39 (s, 3H, O**CH_3_**), 3.52–3.62 (m, 2H, **CH_2_**OCH_3_), 4.06–4.27 (m, 4H, **CH_2_**CH_3_ and COO**CH_2_**), 5.05 (s, 1H, C_4_-**H**), 5.84 (s, 1H, N**H**), 7.33 (t, *J* = 7.66 Hz, 1H, C_5’_-**H**), 7.45 (d, *J* = 7.66 Hz, 1H, C_4’_-**H**), 7.59 (d, *J* = 7.66 Hz, 1H, C_6’_-**H**), 7.61 (s, 1H, C_2’_-**H**). ESI–MS: *m/z* (% abundance): 384.8 (M^+^, 20), 383.8 ([M – 1]^+^, 100). Anal. (%) for C_21_H_24_N_2_O_5_, calcd. (found), C 65.61 (65.35), H 6.29 (6.09), N 7.29 (6.97).

##### 5.1.2.12. (±)-3- Benzyl 5-ethyl 4-(3-cyanophenyl)-2,6-dimethyl-1,4-dihydropyridine-3,5-dicarboxylate (2l)

Yield (1.208g, 76%) as off-white powder, m.p. 115–117 °C. ^1^H NMR (400 MHz, CDCl_3_) δ (ppm): 1.22 (t, *J* = 7.12 Hz, 3H, CH_2_**CH_3_**), 2.37 (s, 3H, C_6_-**CH_3_**), 2.39 (s, 3H, C_2_-**CH_3_**), 4.04–4.16 (m, 2H, **CH_2_**CH_3_), 5.04 (d, *J*_gem_ = 12.40 Hz, 1H, **H**CHC_6_H_5_), 5.05 (s, 1H, C_4_-**H**), 5.16 (d, *J*_gem_ = 12.40 Hz, 1H, HC**H**C_6_H_5_), 5.80 (s, 1H, N**H**), 7.22–7.52 (m, 9H, **C_6_H_5_**, C_2’_-**H**, C_4’_-**H**, C_5’_-**H** and C_6’_-**H**). ^13^C NMR (125 MHz, CDCl_3_) δ (ppm): 14.2 (1 C, CH_2_**CH_3_**), 19.6 ((1 C, C_6_-**CH_3_**), 19.7 (1 C, C_2_-**CH_3_**), 39.7 (1 C, C_4_), 59.9 (1 C, **CH_2_**CH_3_), 65.9 (1 C, **CH_2_**C_6_H_5_), 102.9 (1 C, C_5_), 103.5 (1 C, C_3_), 111.7 (1 C, C_3’_), 119.3 (1 C, CN), 128.0 (2 C, C_2_ and C_6_ of C_6_H_5_), 128.5 (3 C, C_3_, C_4_ and C_5_ of C_6_H_5_), 128.5 (1 C, C_5’_), 129.8 (1 C, C_4’_), 131.9 (1 C, C_6’_), 132.9 (1 C, C_2’_), 136.1 (1 C, C_1_ of C_6_H_5_), 144.3 (1 C, C_6_), 145.2 (1 C, C_6_), 149.0 (1 C, C_1’_), 166.8 (1 C, C_5_-**COO**), 167.0 (1 C, C_3_-**COO**). ESI–MS: *m/z* (% abundance): 416.8 (M^+^, 26), 415.8 ([M – 1]^+^, 100). Anal. (%) for C_25_H_24_N_2_O_4_, calcd. (found), C 72.10 (71.99), H 5.81 (5.96), N 6.73 (6.54).

### 5.2. Pharmacological evaluation of target compounds

#### 5.2.1. Anticancer activity

##### 5.2.1.1. *In vitro* single dose (10 µM) anticancer screening on NCI 60 cancer cell lines

All 60 cancer cell lines were cultured in RPMI 1640 medium. Cells were inoculated into 96 well microtiter plates then incubated in 5% CO_2_, 95% air, and 100% relative humidity for 24h at 37 °C, before the tested compound was added. After 24h, trichloroacetic acid (TCA) was used to fix two plates of each cell line (one as a test and the other as a control) *in situ* to reflect a measurement of cell population for each cell line at time zero of compound addition (Tz). Prior to usage, the tested chemical was solubilised in 400-fold the intended final maximum test concentration in DMSO and stored frozen. The needed final compound concentration was achieved by adding aliquots of 100 μL of this chemical’s solution to appropriate microtiter wells already holding 100 μL of medium. Following the addition of the chemical, the two plates were incubated for an additional 48h at 37 °C, 5% CO_2_, 95% air, and 100% relative humidity. The test was ended by the addition of cold TCA for adhering cells. The cells were fixed *in situ* by gently adding 50 μL of cold 50% (w/v) TCA (final concentration, 10% TCA) and incubating at 4 °C for 60 min. The supernatant was discarded, and the plates were rinsed and air dried five times with tap water. SRB solution (100 μL) containing 0.4% (w/v) sulforhodamine in 1% acetic acid was added to each well, and plates were incubated at room temperature for 10 min. The bound dye was then solubilised with 10 mM trizma base, and the absorbance was measured at 515 nm using an automated plate reader.^57^^,^^58^

##### 5.2.1.2. *In vitro* cytotoxicity of target compounds, 1g, 2e, and 2l in human HCT-116 and HCT-116/ADR cells and their potentiation of DOX cytotoxicity in drug-resistant HCT-116/ADR cell

We investigated the reversal DOX effect of the three most active compounds, **1g**, **2e**, and **2l**, and assessed the IC_50_ of DOX and RF, which was measured by dividing the IC_50_ (DOX) values without P-gp modulators by IC_50_ (DOX) values with P-gp modulators as previously described.^77^^,^^78^

##### 5.2.1.3. Mechanistic insight of 1g, 2e, and 2l induced cytotoxicity

EGFR, HER-2, and BTK inhibitory activity of the potent antitumor compounds **1g**, **2e**, and **2l** were studied, and results were displayed as IC_50_ (nM) and % potency, and they are compared to erlotinib as a reference drug as previously described.^15^

##### 5.2.1.4. Annexin V–FITC apoptosis assay

PS externalisation was identified using the apoptosis detection kit (Annexin V-FITC/PI) (BD Biosciences) in accordance with the manufacturer’s guidelines.^79^

##### 5.2.1.5. *In vitro* cell cycle analysis

HCT-116 cells were treated for 24h with the IC_50_ concentrations of DHPs **1g**, **2e**, and **2l**, then washed three times with cold phosphate buffered saline. The cells were centrifuged, frozen in cold 75% ethanol, washed in phosphate buffered saline, resuspended with 100 mg/mL RNase, then stained with 40 mg/mL PI, and analysed with a FACS Calibur (Becton Dickinson, BD, Franklin Lakes, NJ). The cell cycle distributions were determined using Becton Dickinson’s CellQuest software version 5.1.^80^

#### 5.2.2. Antimicrobial activity

##### 5.2.2.1. *In vitro* antibacterial and antifungal evaluation

By the agar well diffusion method, all of the synthesised compounds were individually evaluated against six pathogen strains; two gram-positive bacteria (*S. aureus* and *B. subtilis*), two gram-negative bacteria (*E. coli* and *P. aeruginosa*), and two fungi (*C. albicans* and *A. flavus*). To make a 1 mg/mL solution, each chemical was dissolved separately and aseptically in DMSO. Whatman filter paper discs of a standard size (5 mm diameter) were manufactured, cut, and sterilised in an autoclave. The paper discs were soaked in the compound solution to the necessary concentration before being inserted aseptically on petri plates containing nutrient agar media (20g agar, 3g beef extract, and 5g peptone) inoculated with the pathogen under study. After 24h of incubation at 36 °C, the inhibition zones were measured in millimetres. Standard antibacterial and antifungal compounds, ciprofloxacin and clotrimazole, were employed in the same operation and under the same settings. As a control, DMSO solvent was employed. Three times each treatment was carried out.^51^^,^^67^

##### 5.2.2.2. Determination of minimum inhibitory concentration (MIC)

The MIC of selected compounds **1f**, **1g**, **2a**, **2g**, **2j**, **and 2k** were determined by using the two-fold microbroth dilution method. The selected compounds’ solutions in different concentrations of 64, 32, 16, 8, 4, 2, 1, and 0.5 µg/mL were aseptically prepared using DMSO as a solvent and put in different wells. The broth containing the tested pathogen suspension at 106 CFU/mL was added evenly to each well. The sealed plates were incubated at 36 °C for 24h. Ciprofloxacin and clotrimazole were used as standard antibacterial and antifungal agents, respectively, using the same procedure under the same conditions. Positive control of wells with DMSO and inoculated media and negative control of wells with DMSO and uninoculated media were run parallel to each tested compound experiment. Each experiment was performed in triplicate. MIC was determined as the lowest concentration that had no visible turbidity.^68^

### 5.3. Molecular docking

For complex modelling, the RSCB Protein Data Bank was used to obtain the structure of various proteins (PDB IDs: 4MS2, 3G60, 1M17, 3RCD, and 4Z3V). The MOE suite was utilised to create target compound structures. A Tripos force field and energy minimisation were used to optimise the structure in vacuum. The partial atomic charges were calculated using the Gasteiger–Huckel method. The docking was done with the MOE package. The MOE Tools package was used to set the docking parameters. The ligand poses obtained through docking were graded and chosen based on the values of their scoring functions and poses in the binding site. Crystal structure ligand locations were used as a reference template to assess the docked molecules’ correctness.^81^

## Supplementary Material

Supplemental Material
