# Supplementary material for: Discovery of new symmetrical and asymmetrical nitrile-containing 1,4-dihydropyridine derivatives as dual kinases and P-glycoprotein inhibitors: synthesis, in vitro assays, and in silico studies
Source: J Enzyme Inhib Med Chem. 2022 Sep 12;37(1):2489–511. doi: 10.1080/14756366.2022.2120478 (PMC9481151; doi:10.1080/14756366.2022.2120478)
Supplement: Supplemental Material [file IENZ_A_2120478_SM9051.pdf]

Discovery of new symmetrical and asymmetrical nitrile-containing 1,4-dihydropyridine derivatives as dual kinases and P-glycoprotein inhibitors: Synthesis, *in vitro* assays and *in silico* studies

Mohamed H. Saad[1], Tarek F. El-Moselhy[2], Nabaweya S. El-Din[2], Ahmed B. M. Mehany[3], Amany Belal[4,5], Mohammad A.S. Abourehab[6,7], Haytham O. Tawfik[2,*], Mervat H. El-Hamamsy[2]

Supplementary Material

Mechanism of formation of 1,4-DHP **1a-h** …………….... p2-p4
Mechanism of formation of 1,4-DHP **2a-l** ……………..... p5
Spectral data section ……………………………….…… p6-p27
Biological section ……………………………………….. p28-p47
Docking section …………………………………….…..... p48-p49
References ……………………………………….…..….. p50

Mechanism of formation of 1,4-DHP **1a-h**

The first series, eight symmetric achiral 1,4-DHPs **1a-h**, was synthesized by the classical Hantsch reaction for direct synthesis of symmetric 1,4-DHP [1]. The classical Hantsch reaction is one of the most popular multicomponent condensation reactions (MCRs) [2] of an aldehyde, a β-ketoester (2 equivalents) and ammonia or ammonium salts such as ammonium acetate as declared in **Scheme S.1**.

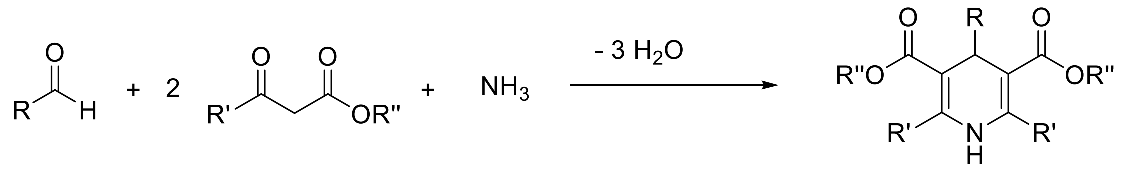

Aldehyde β-ketoester Ammonia 1,4-DHP **1a-h**

Scheme S.1. General classical Hantzsch reaction for synthesis of 1,4-DHP.

Many scientists studied the Hantzsch reaction trying to realize the mechanism by which the reaction undergoes, so at least five major different mechanistic pathways were proposed [3]. Extensive studies of the mechanism of Hantzsch reaction were made by monitoring the reaction intermediates and elucidating their structures by ^{15}N NMR, ^{13}C NMR [4] and mass spectroscopy [3], revealing that the reaction proceeds via a mechanism in which enamine intermediate combines with typical Knoevenagel product [5]. So, the Hantzsch reaction can be illustrated by three main steps as the following [2]:

Step 1: Condensation of ammonia with β-ketoester to afford the ester enamine intermediate (intermediate **1**), **Scheme S.2**.

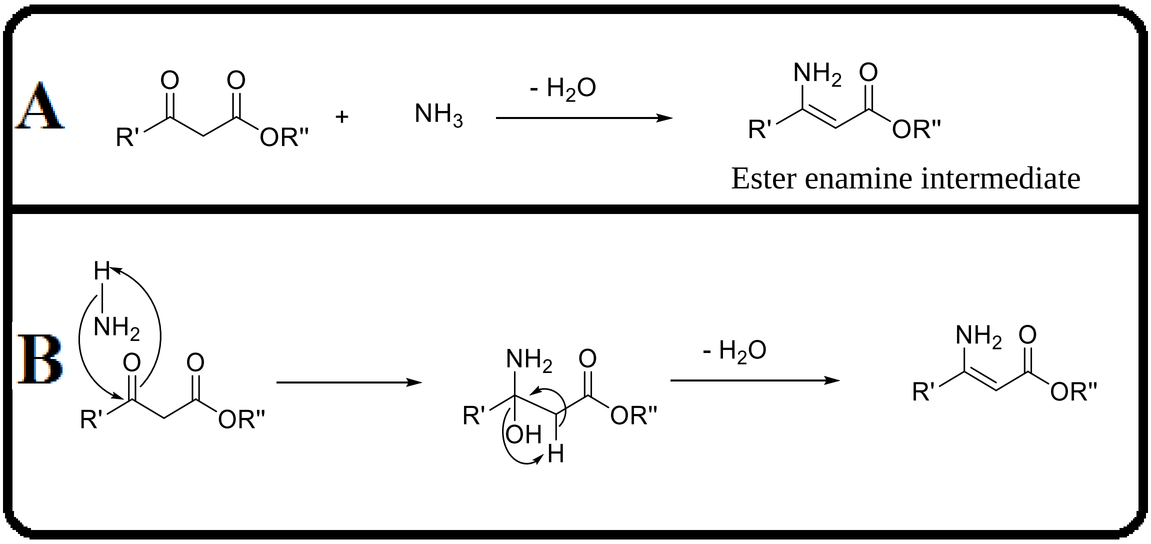

Scheme S.2. Formation of ester enamine intermediate (intermediate **1**) (A) and its proposed mechanism (B) [2].

Step 2: Condensation of aldehyde with the second equivalent of the β-ketoester form a typical Knoevenagel product (intermediate **2**) as shown in **Scheme S.3**. Intermediate **2** is called arylidene when aryl aldehyde reacts, whereas it is called alkylidene when alkyl aldehyde reacts.

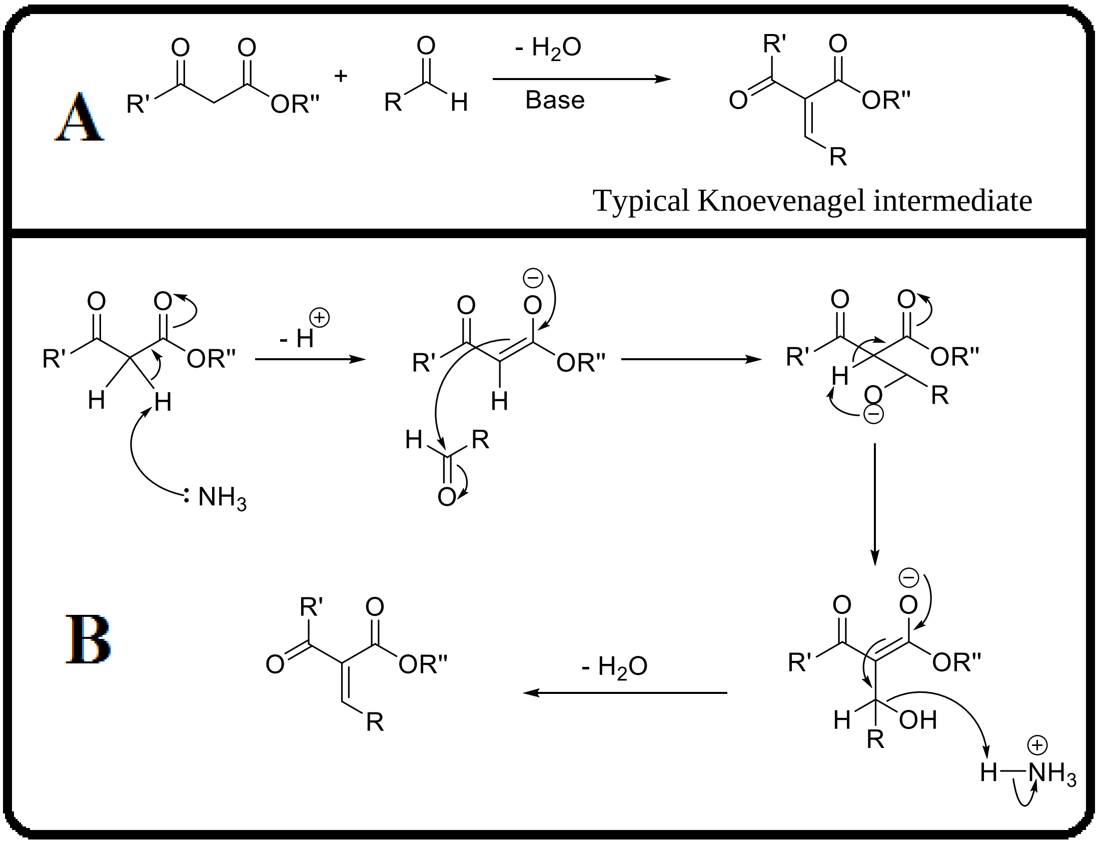

Scheme S.3. Construction of typical Knoevenagel product (intermediate 2) (A) and its proposed mechanism (B) [2].

Step 3: Finally, 1,4-DHPs were prepared via condensation of intermediate **1** and intermediate **2** through Michael addition as displayed in **Scheme S.4**.

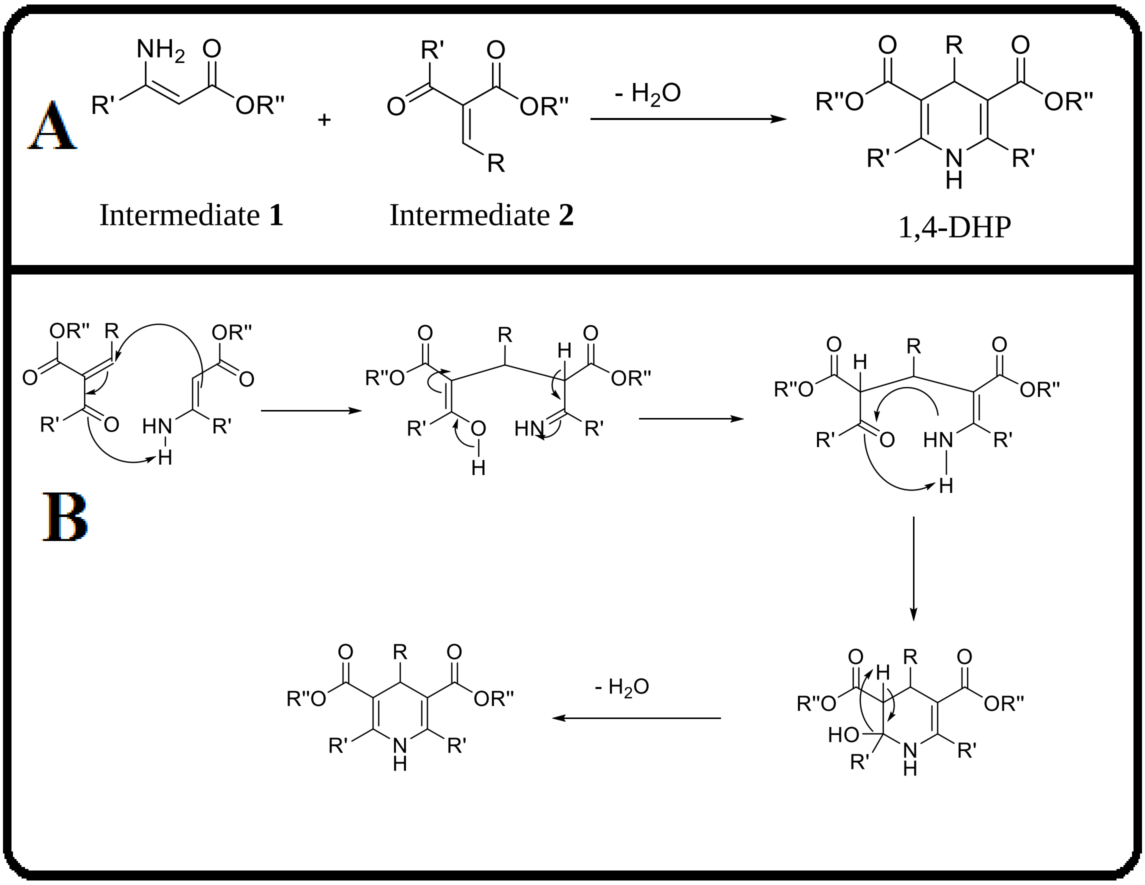

Scheme S.4. Preparation of 1,4-DHP derivatives (A) and its proposed mechanism (B) [2].

<u>Mechanism of formation of 1,4-DHP **2a-l**</u>

The second series, twelve asymmetric chiral 1,4-DHPs **2a-l**, was synthesized via Iwanami reported method [6,7] which is a modified Hantsch reaction. This modified reaction is a one-pot condensation reaction between aldehyde, β-ketoester and an alkyl 3-aminocrotonate, **Scheme S.5**.

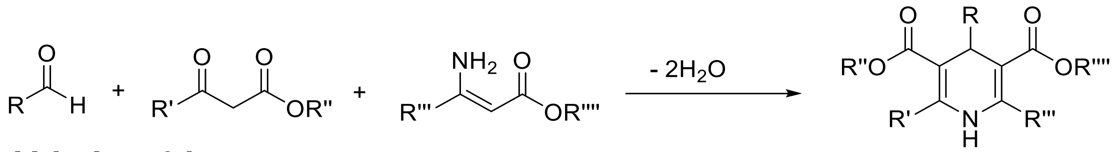

Aldehyde β-ketoester alkyl 3-aminocrotonate 1,4-DHP **2a-l**

Scheme S.5. Iwanami method for synthesis of asymmetric chiral 1,4-DHPs.

Iwanami modified reaction can be illustrated by 2 main steps as the following:

<u>Step 1</u>: Formation of the typical Knoevenagel product by the reaction of the aldehyde with the β-ketoester as the same manner of the information of intermediate **2** in **Scheme S.3**.

<u>Step 2</u>: Establishment of the 1,4-DHP derivative through, condensation of intermediate **2** and alkyl 3-aminocrotonate as reported in **Scheme S.4**, while the formed 1,4-DHP is an asymmetric chiral derivative.

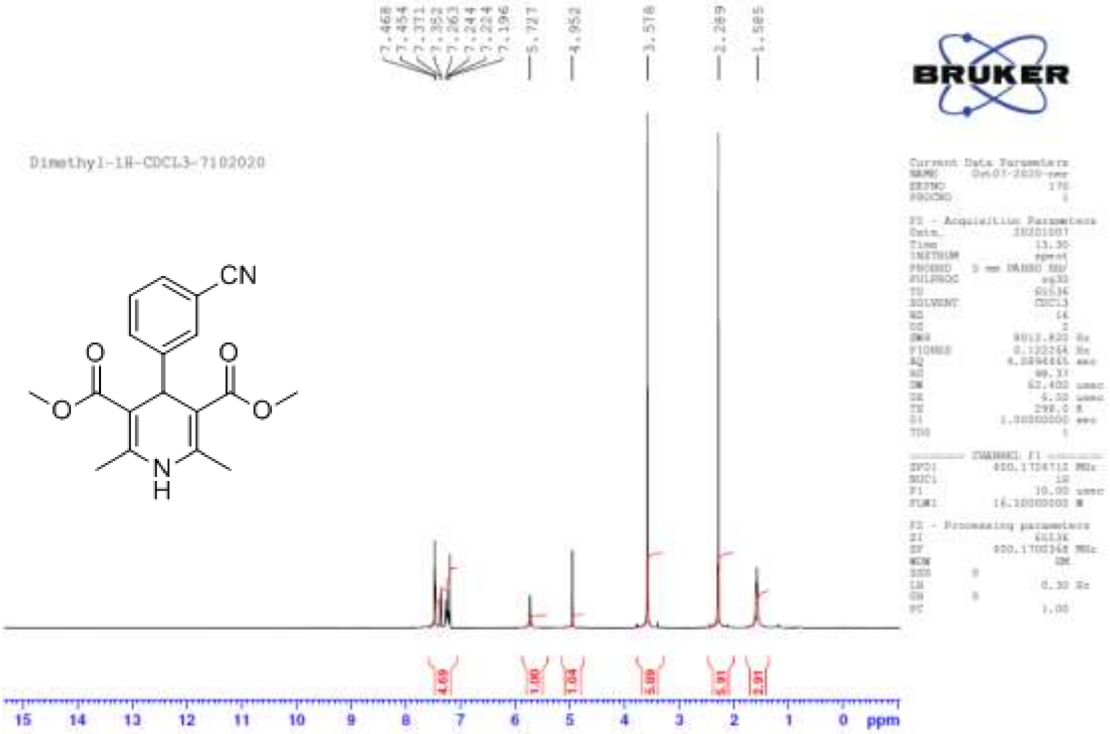

Figure S1. ^{1}H NMR (400 MHz, $CDCl_3$) spectrum of compound **1a**

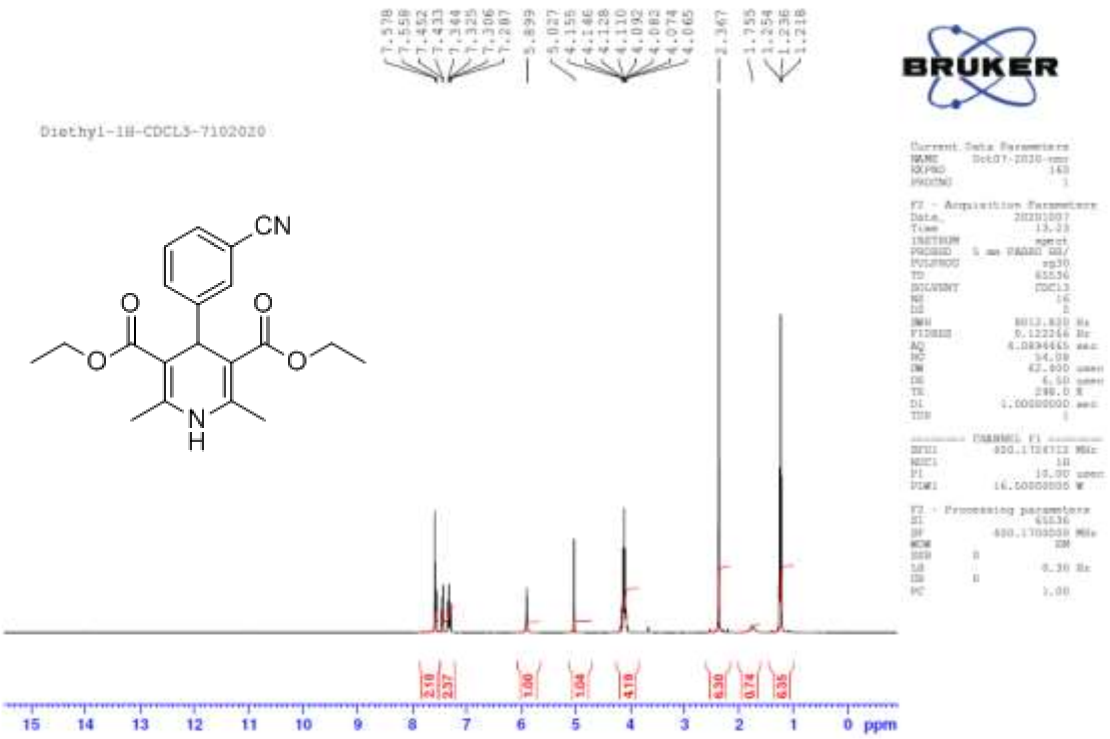

Figure S2. ^{1}H NMR (400 MHz, $CDCl_3$) spectrum of compound **1b**

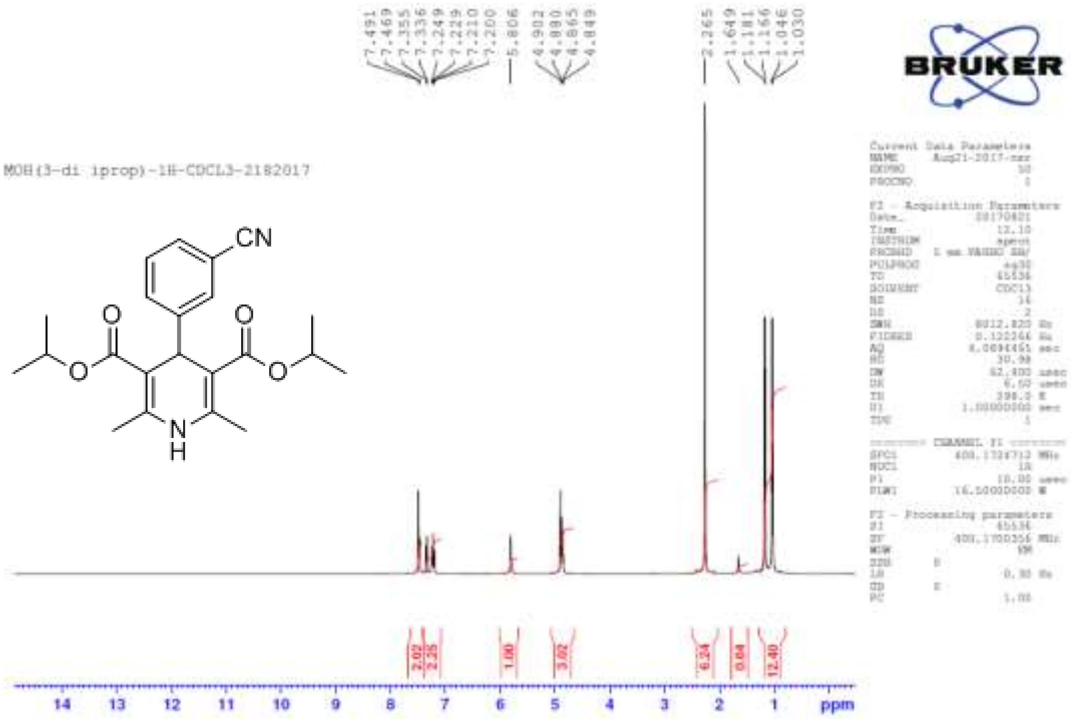

Figure S3. ^{1}H NMR (400 MHz, $CDCl_3$) spectrum of compound **1c**

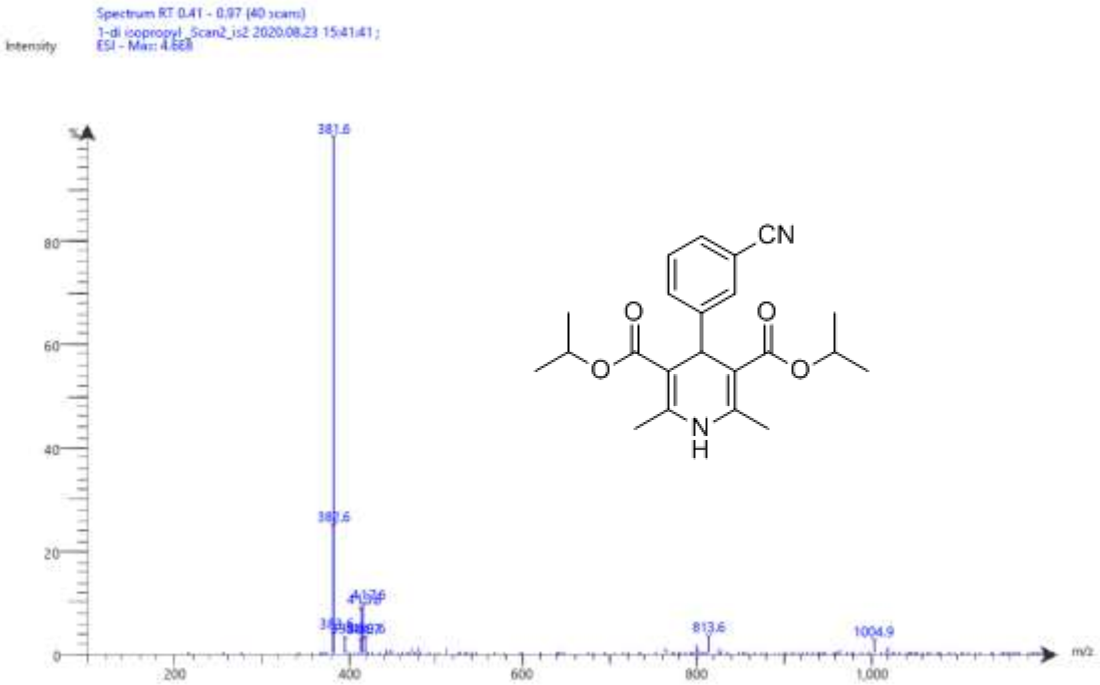

Figure S4. Negative ion ESI-MS spectrum of compound **1c**

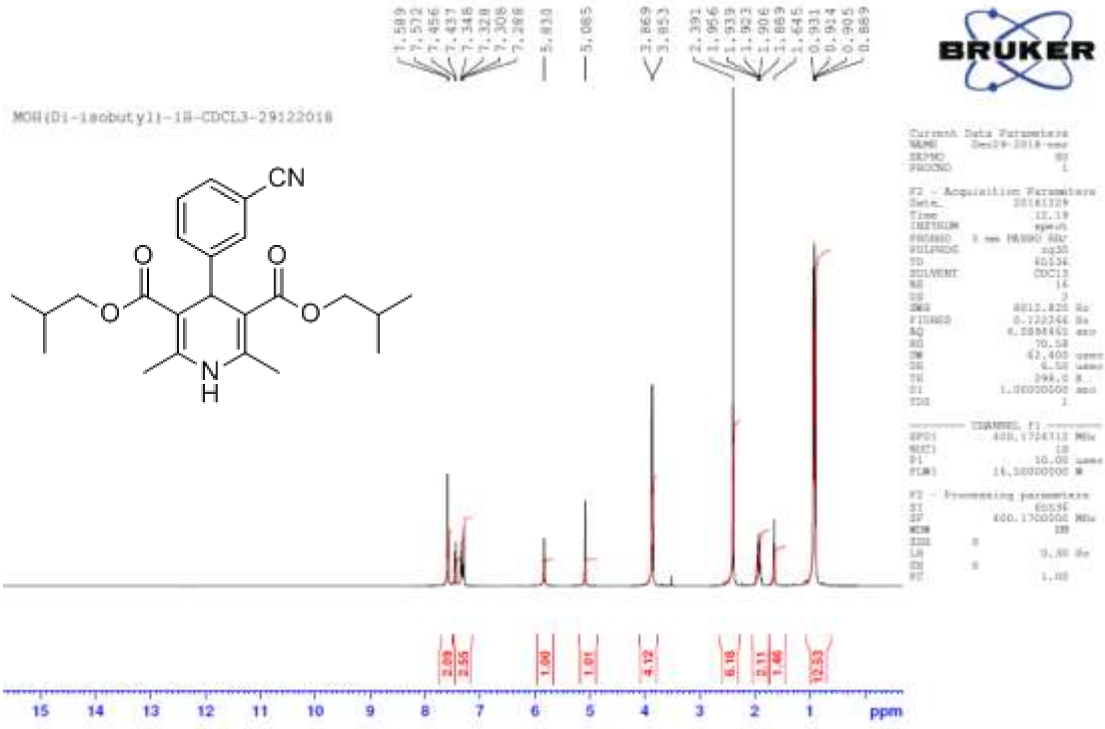

Figure S5. ^1H NMR (400 MHz, $CDCl_3$) spectrum of compound **1d**

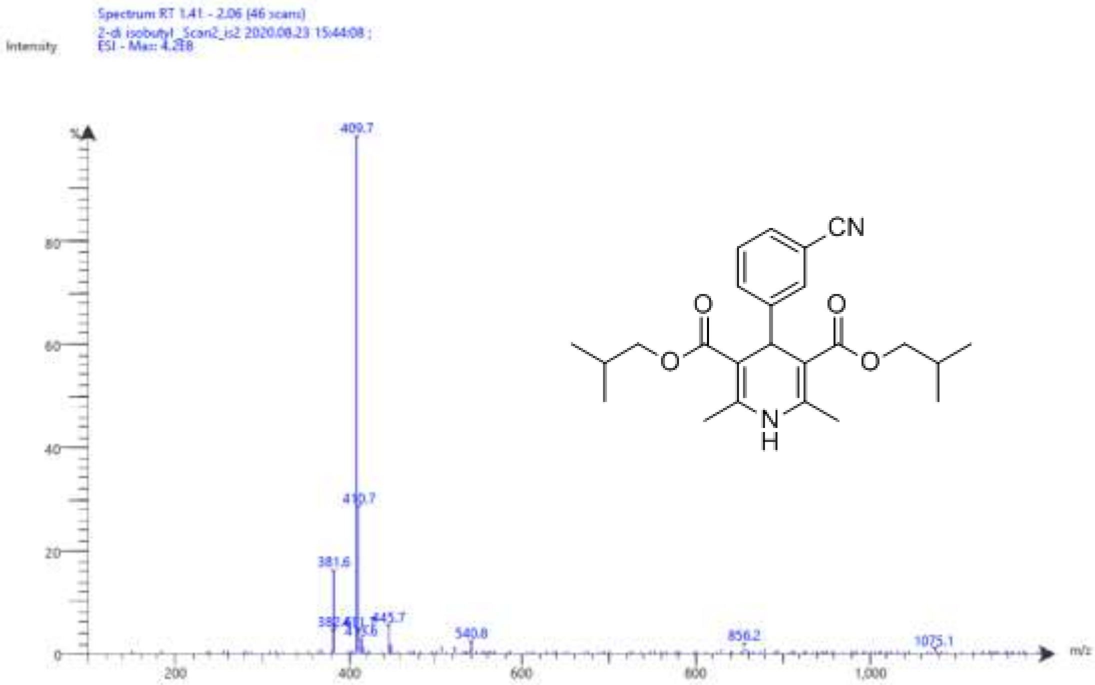

Figure S6. Negative ion ESI-MS spectrum of compound **1d**

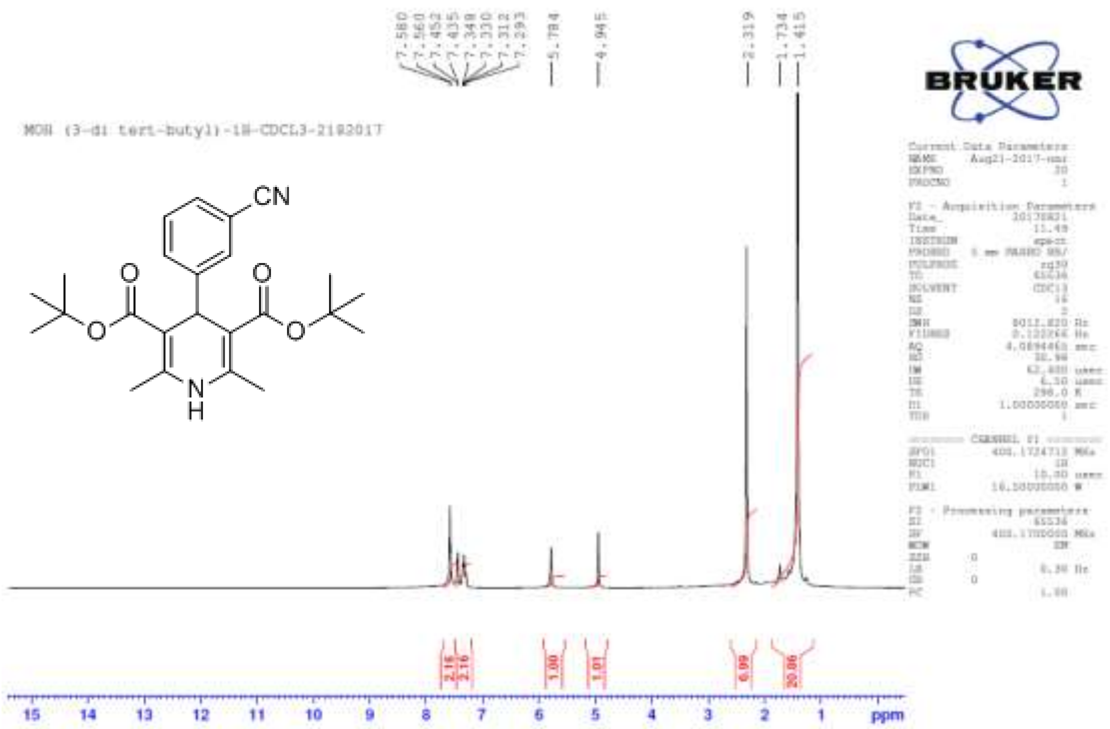

Figure S7. ^{1}H NMR (400 MHz, $CDCl_3$) spectrum of compound **1e**

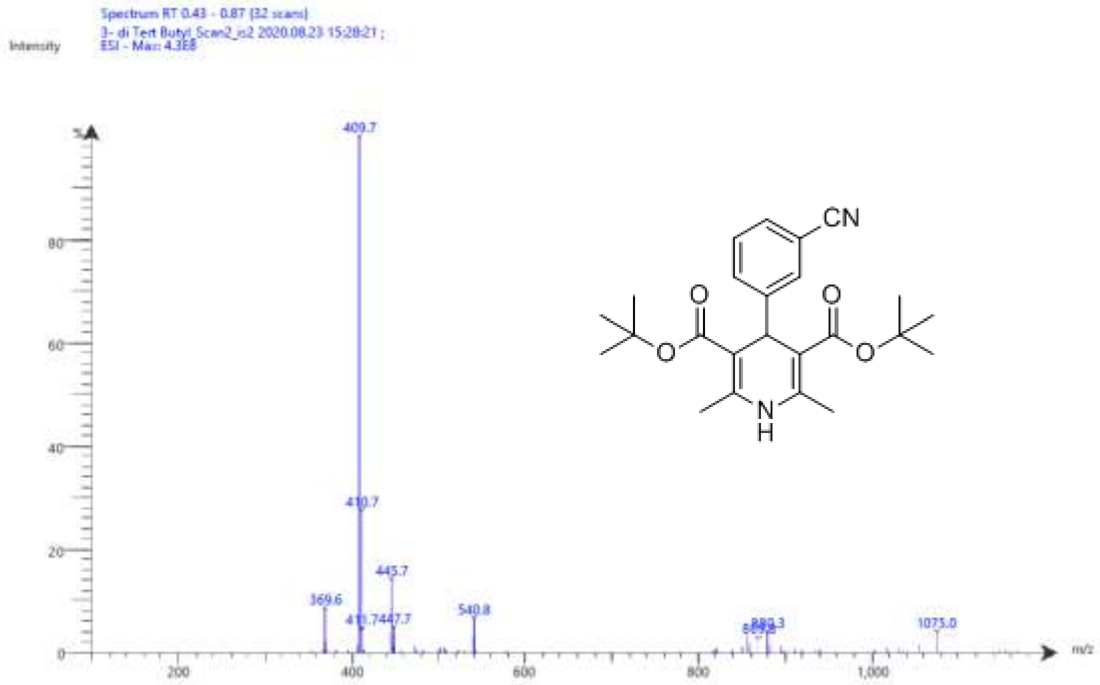

Figure S8. Negative ion ESI-MS spectrum of compound **1e**

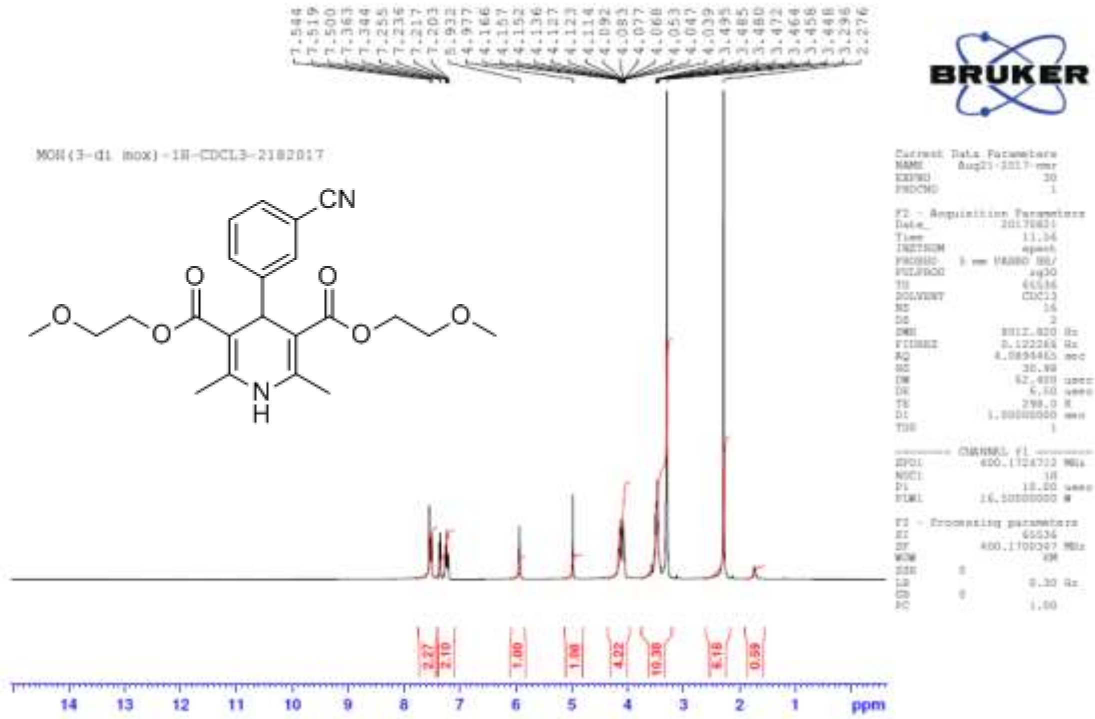

Figure S9. ^{1}H NMR (400 MHz, $CDCl_3$) spectrum of compound **1f**

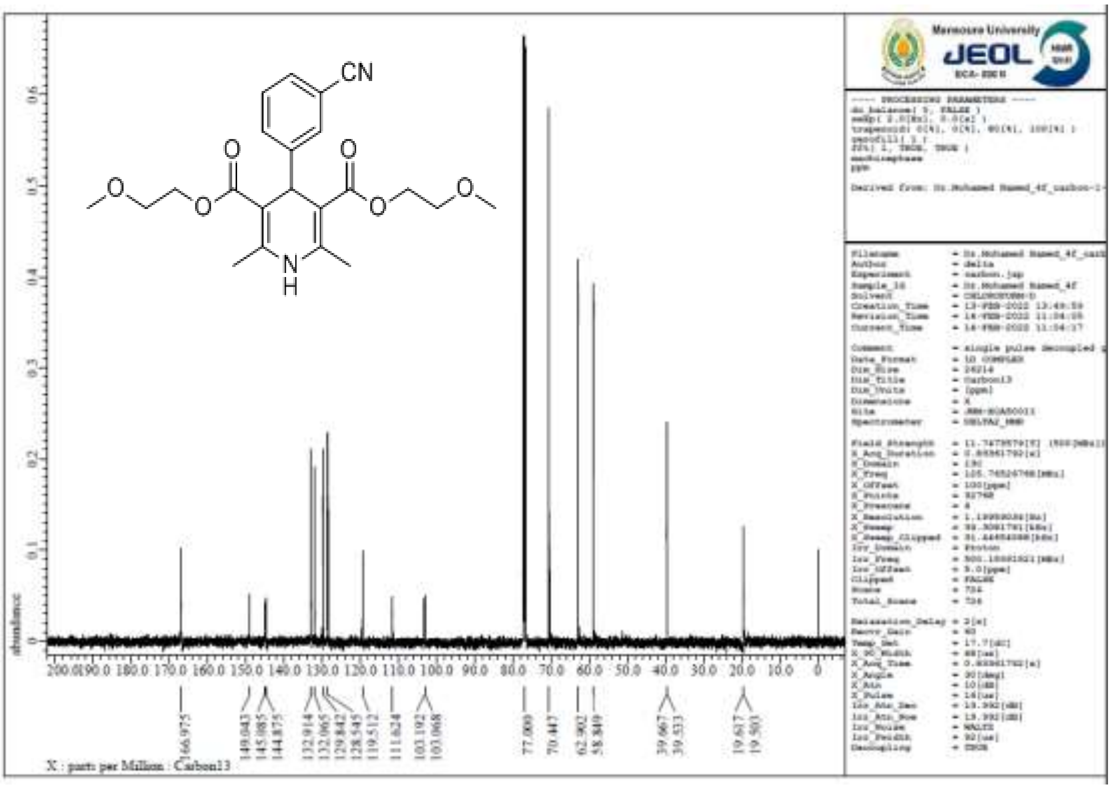

Figure S10. ^{13}C NMR (125 MHz, $CDCl_3$) spectrum of compound **1f**

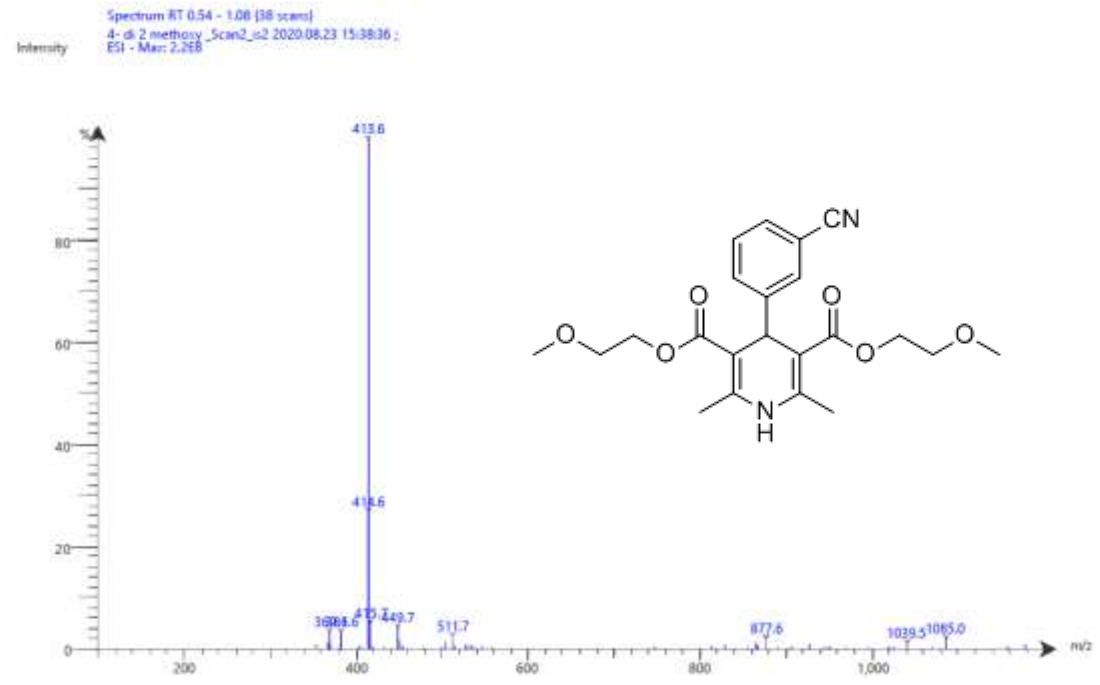

Figure S11. Negative ion ESI-MS spectrum of compound **1f**

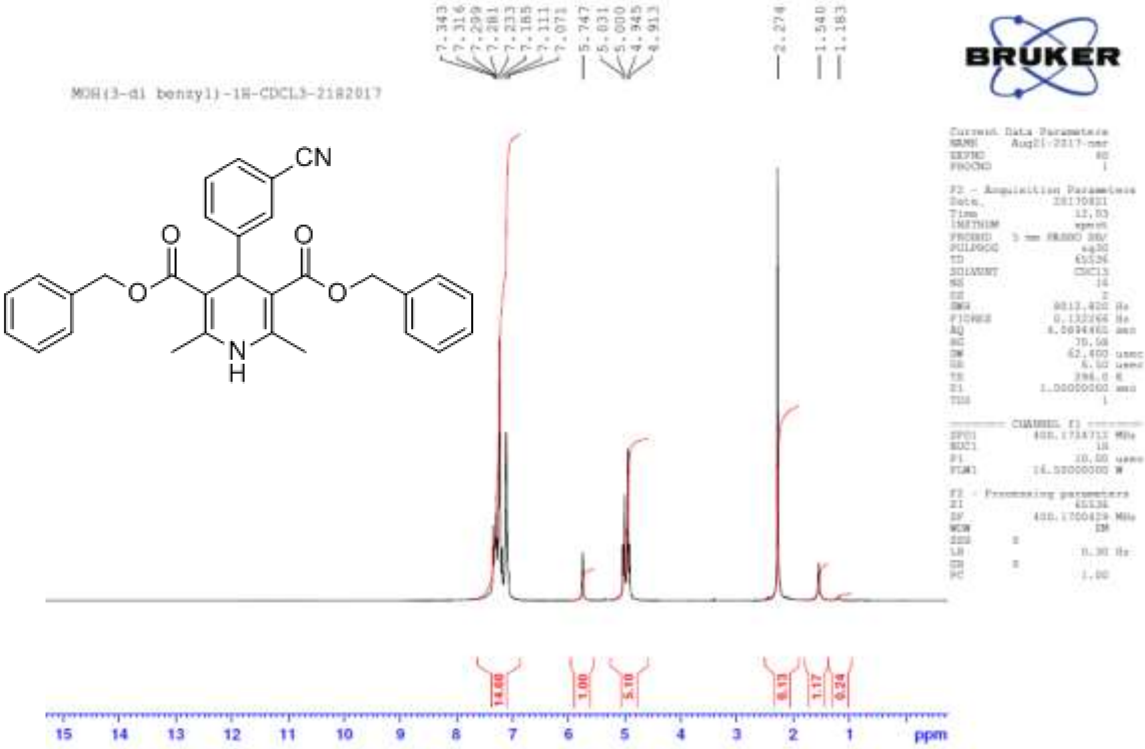

Figure S12. ^{1}H NMR (400 MHz, $CDCl_3$) spectrum of compound **1g**

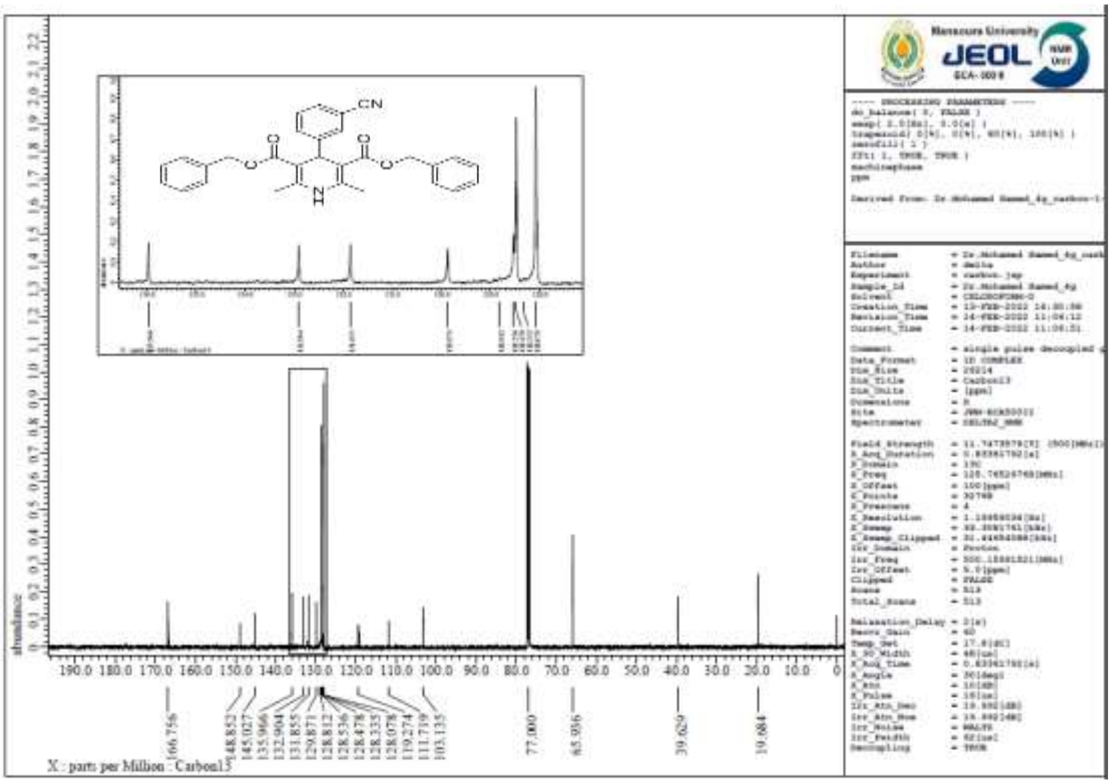

Figure S13. ^{13}C NMR (125 MHz, $CDCl_3$) spectrum of compound **1g**

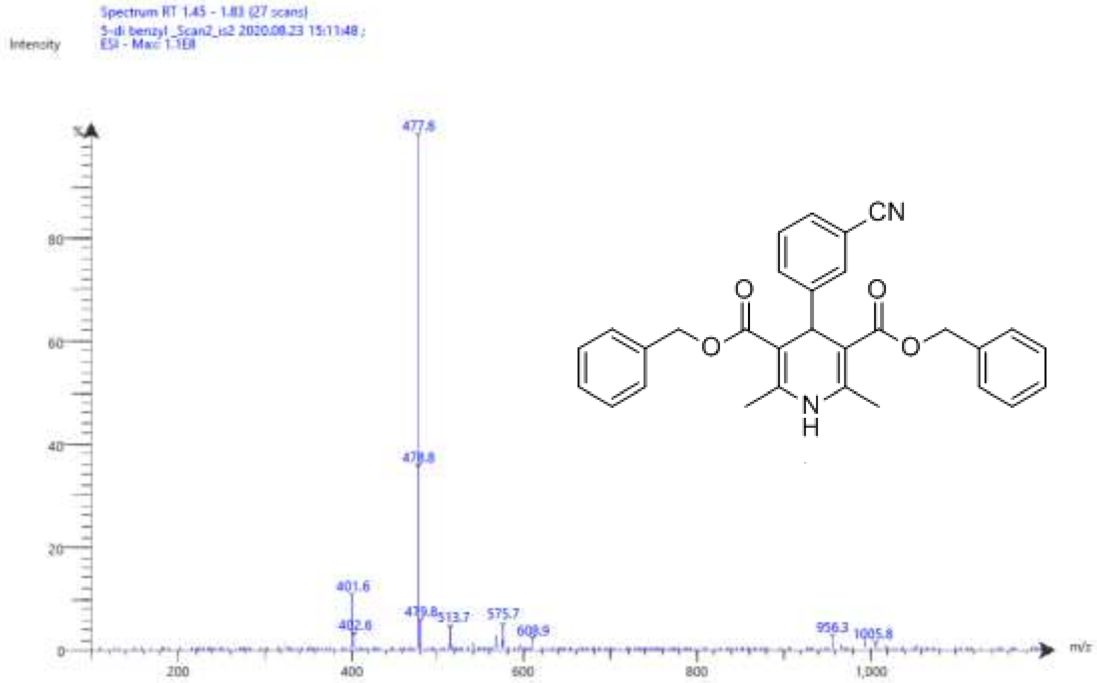

Figure S14. Negative ion ESI-MS spectrum of compound **1g**

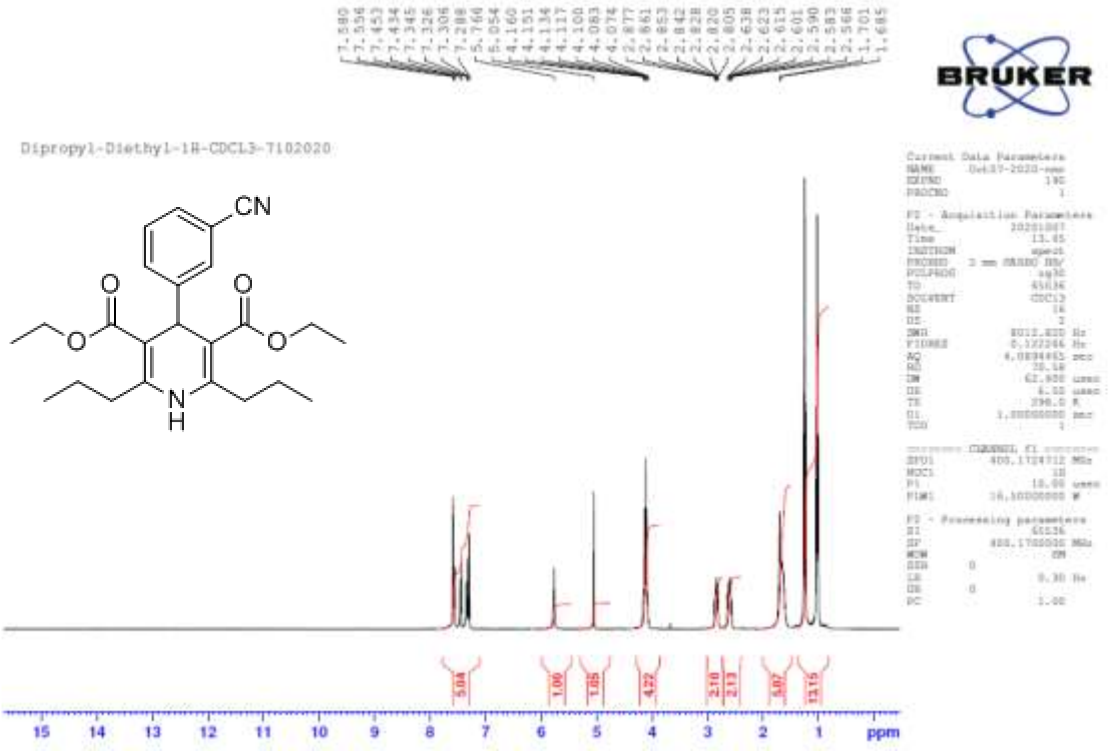

Figure S15. ^{1}H NMR (400 MHz, $CDCl_3$) spectrum of compound **1h**

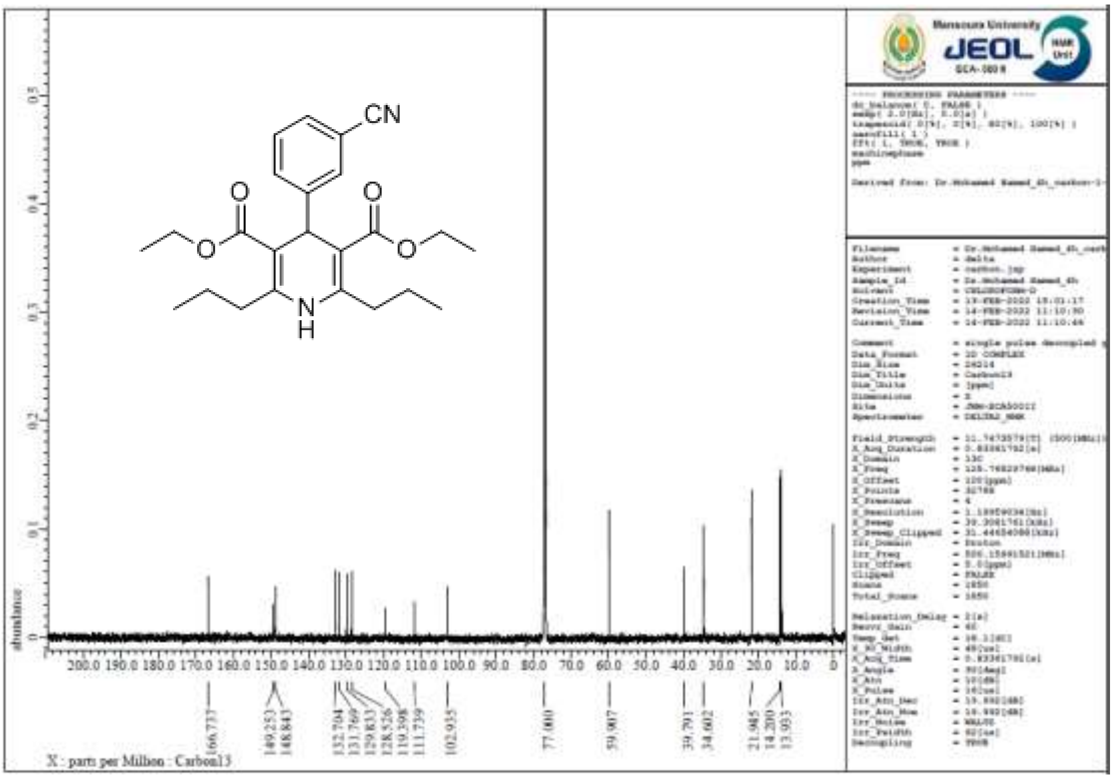

Figure S16. ^{13}C NMR (125 MHz, $CDCl_3$) spectrum of compound **1h**

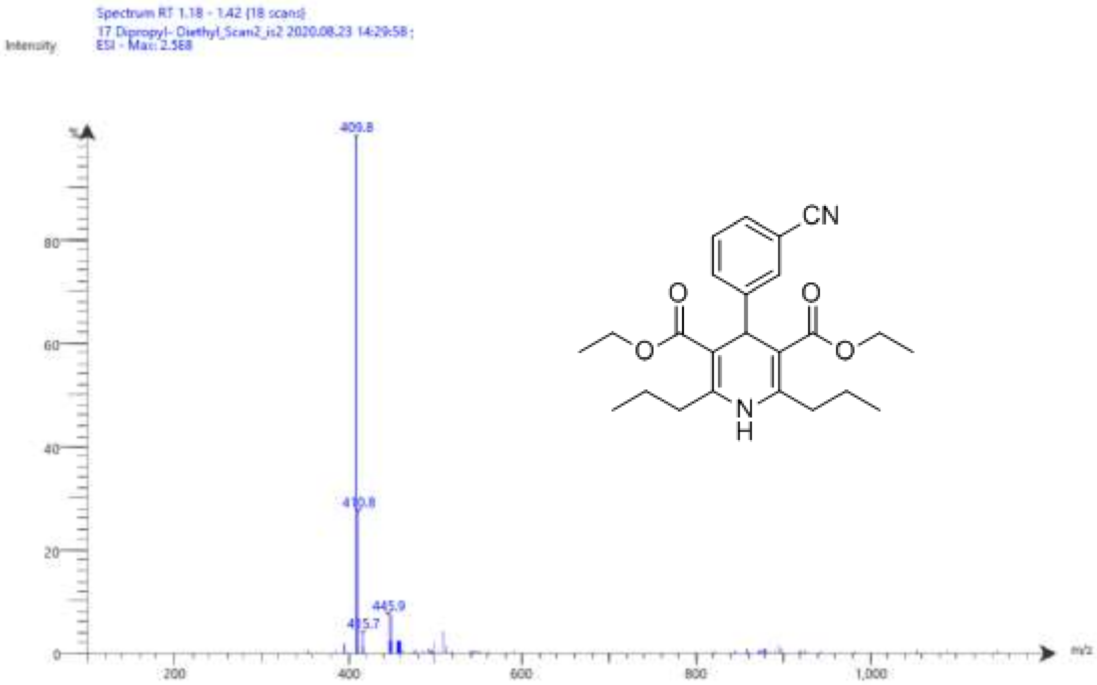

Figure S17. Negative ion ESI-MS spectrum of compound **2h**

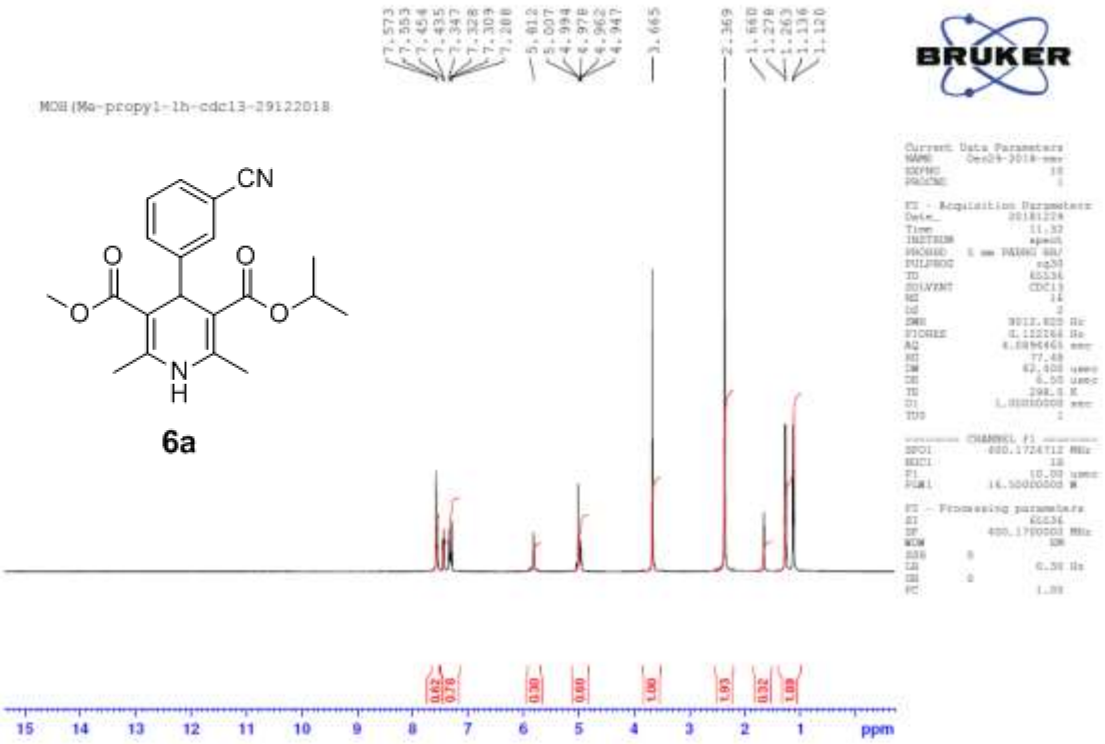

Figure S18. ^{1}H NMR (400 MHz, $CDCl_3$) spectrum of compound **2a**

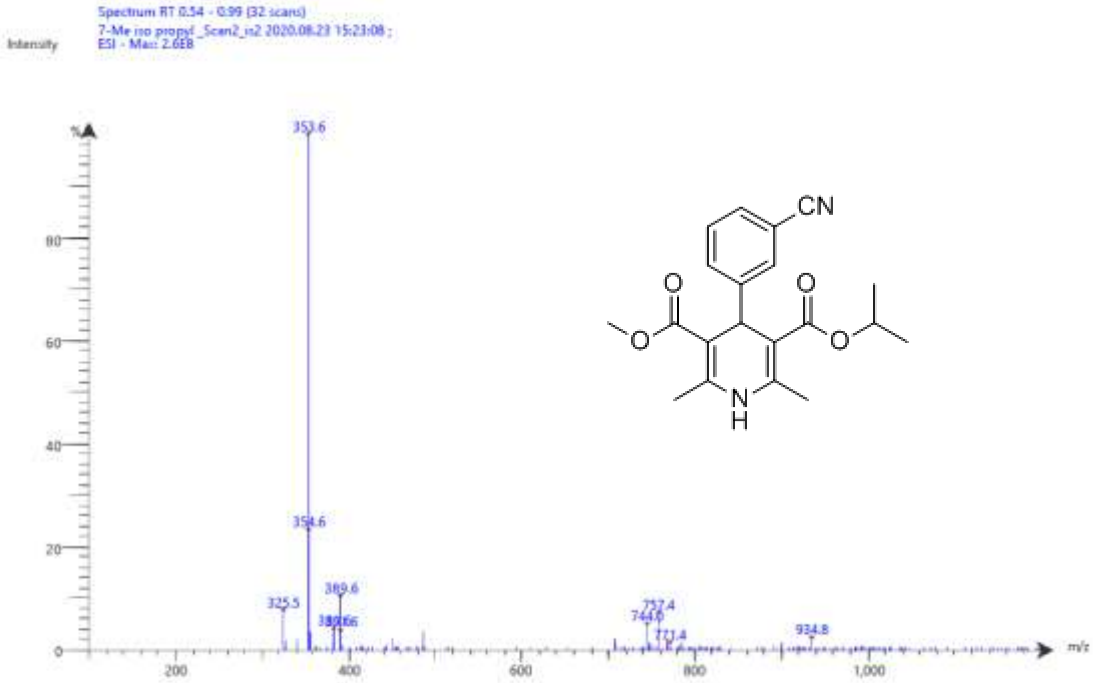

Figure S19. Negative ion ESI-MS spectrum of compound **2a**

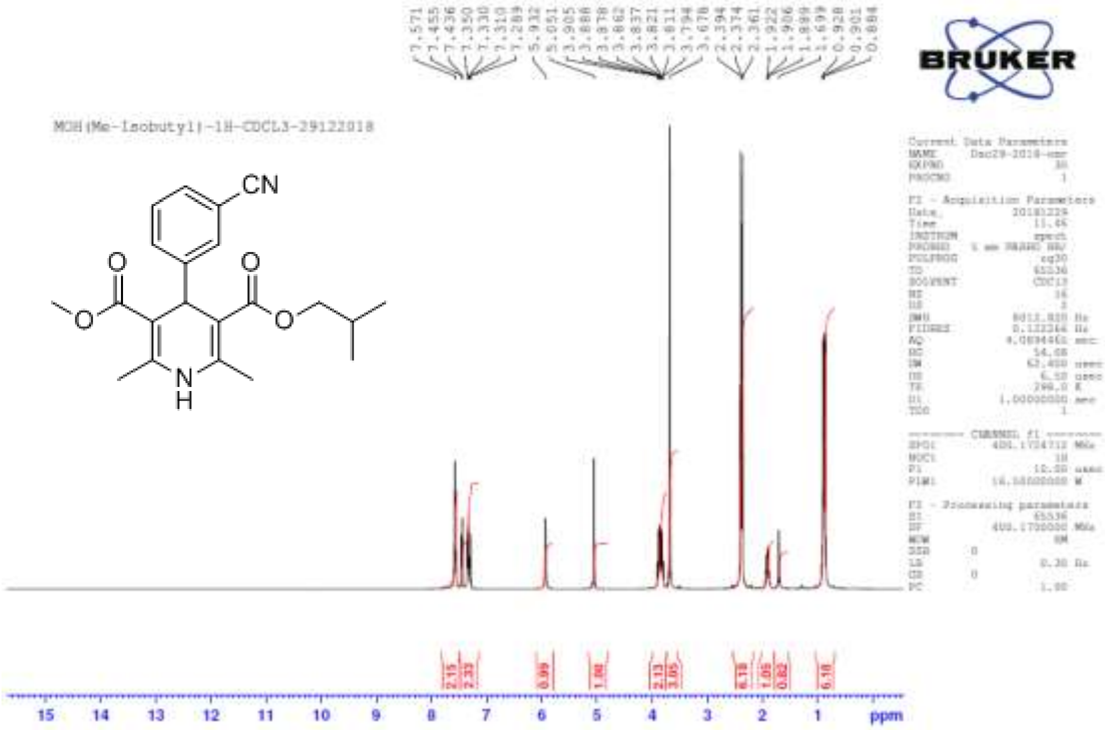

Figure S20. ^1H NMR (400 MHz, $CDCl_3$) spectrum of compound **2b**

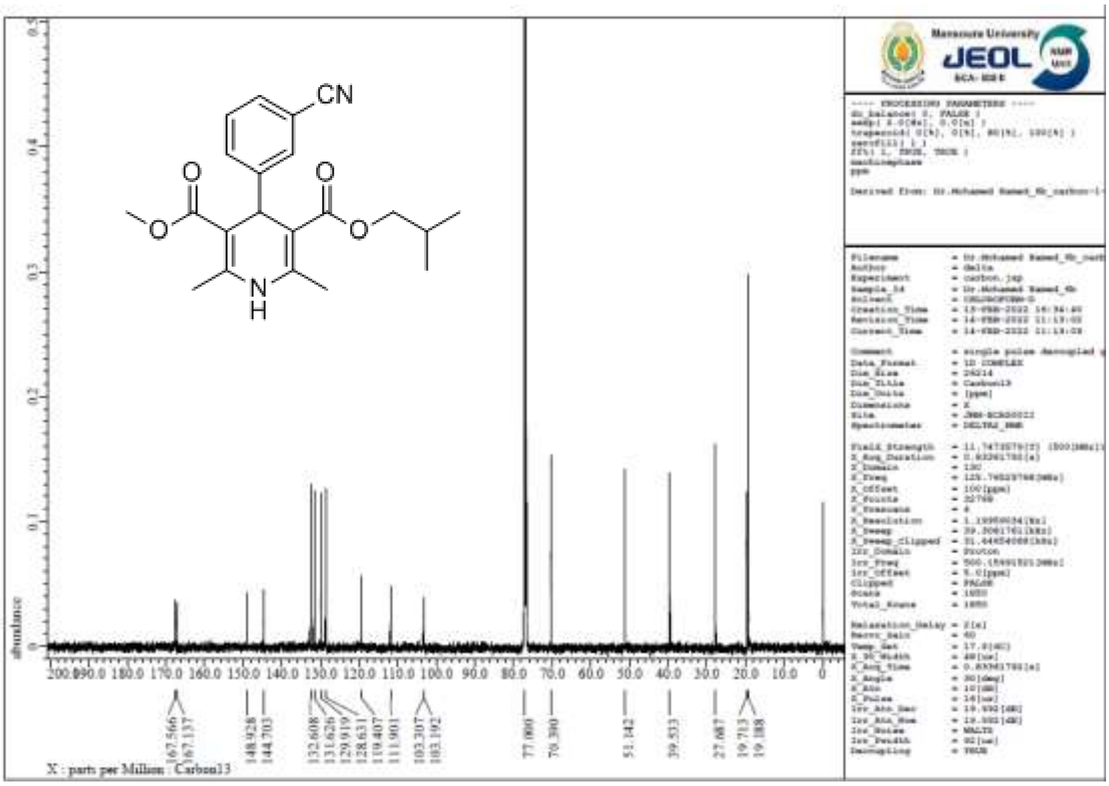

Figure S21. ^{13}C NMR (125 MHz, $CDCl_3$) spectrum of compound **2b**

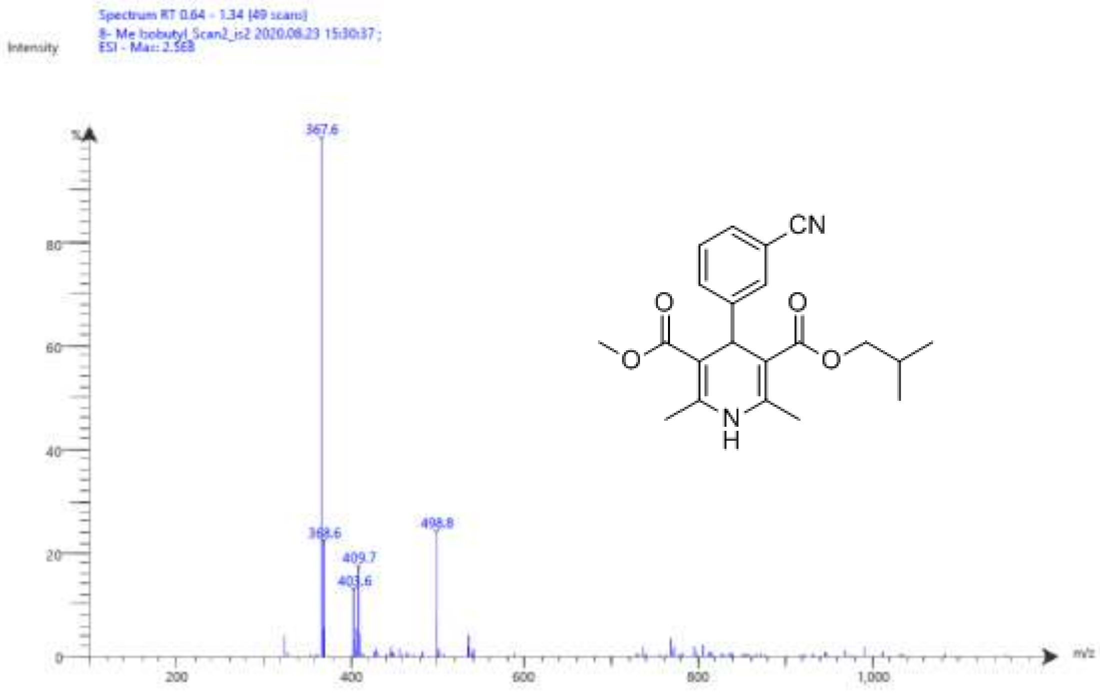

Figure S22. Negative ion ESI-MS spectrum of compound **2b**

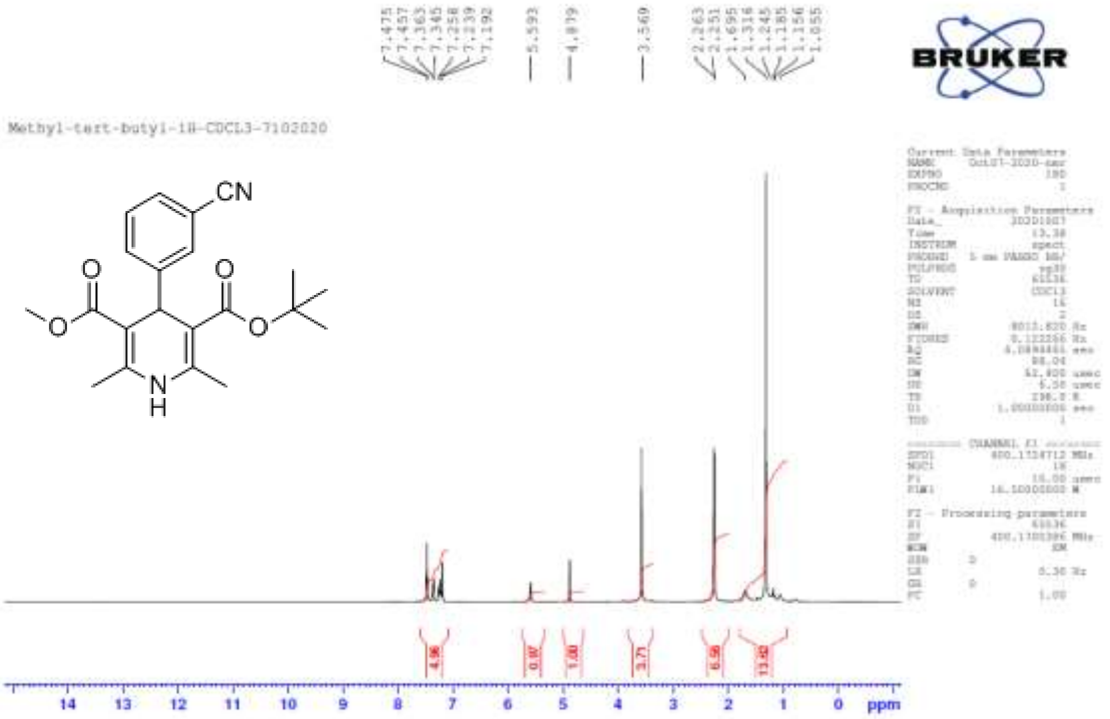

Figure S23. ^{1}H NMR (400 MHz, $CDCl_3$) spectrum of compound **2c**

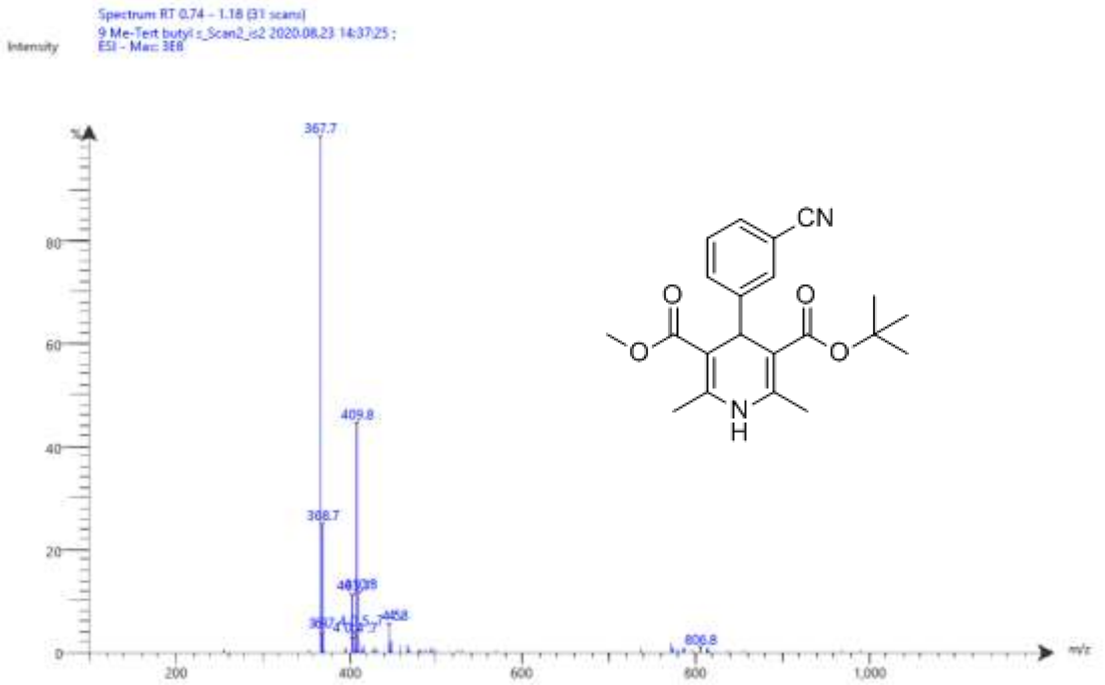

Figure S24. Negative ion ESI-MS spectrum of compound **2c**

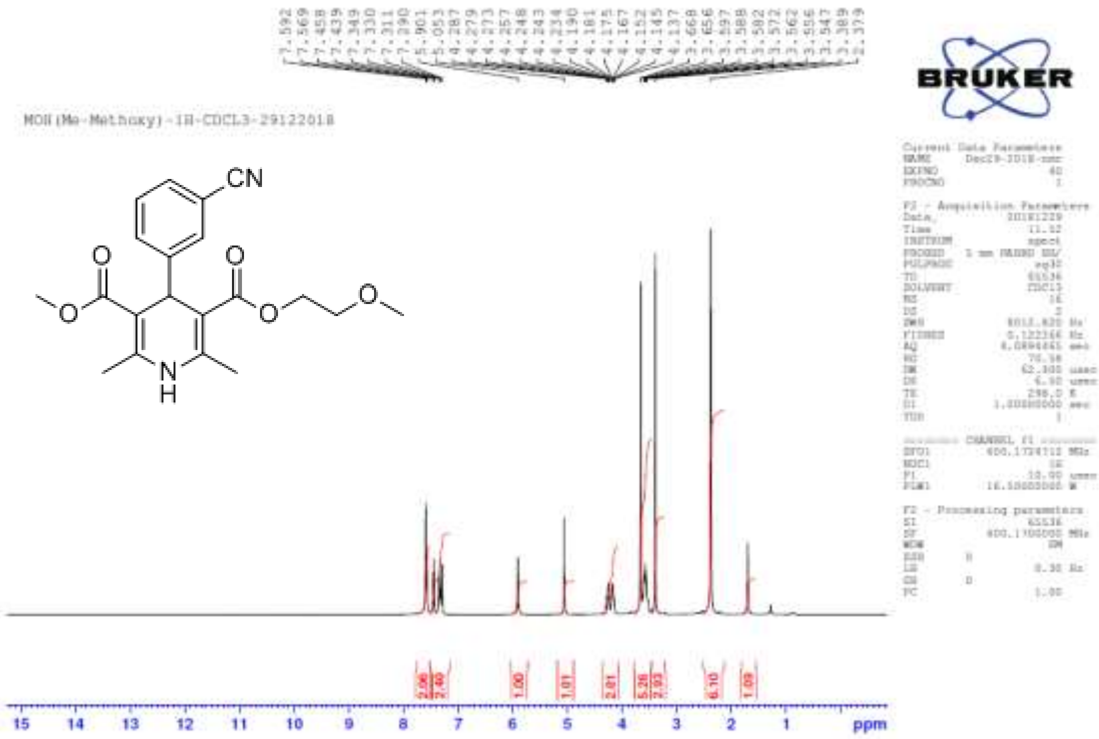

Figure S25. ^{1}H NMR (400 MHz, $CDCl_3$) spectrum of compound **2d**

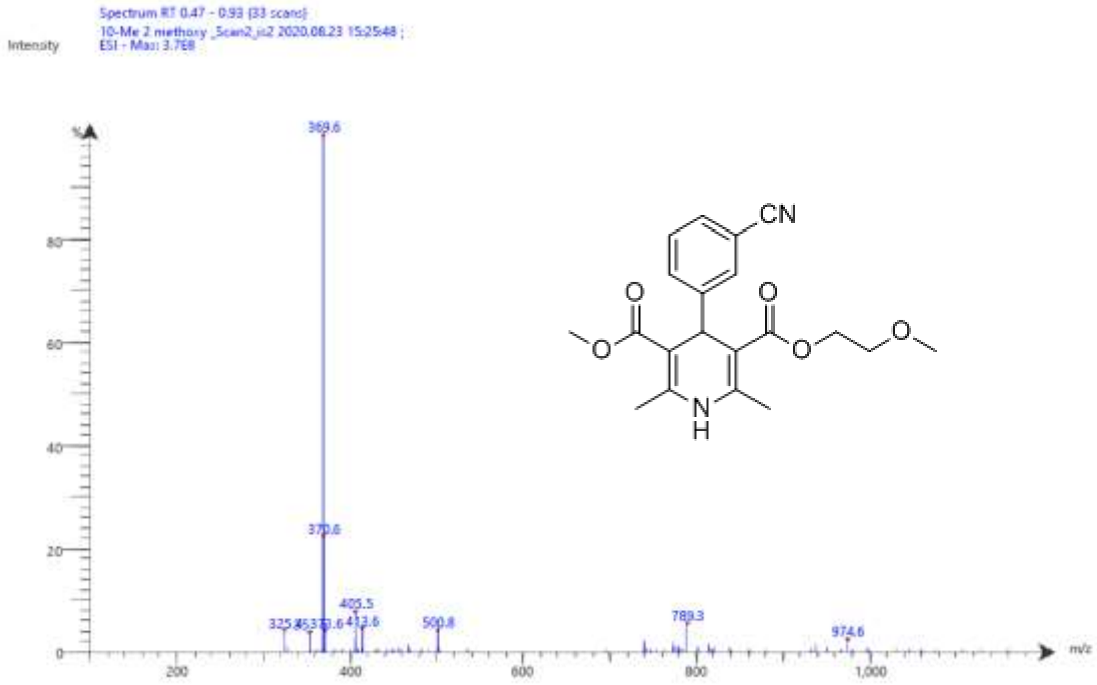

Figure S26. Negative ion ESI-MS spectrum of compound **2d**

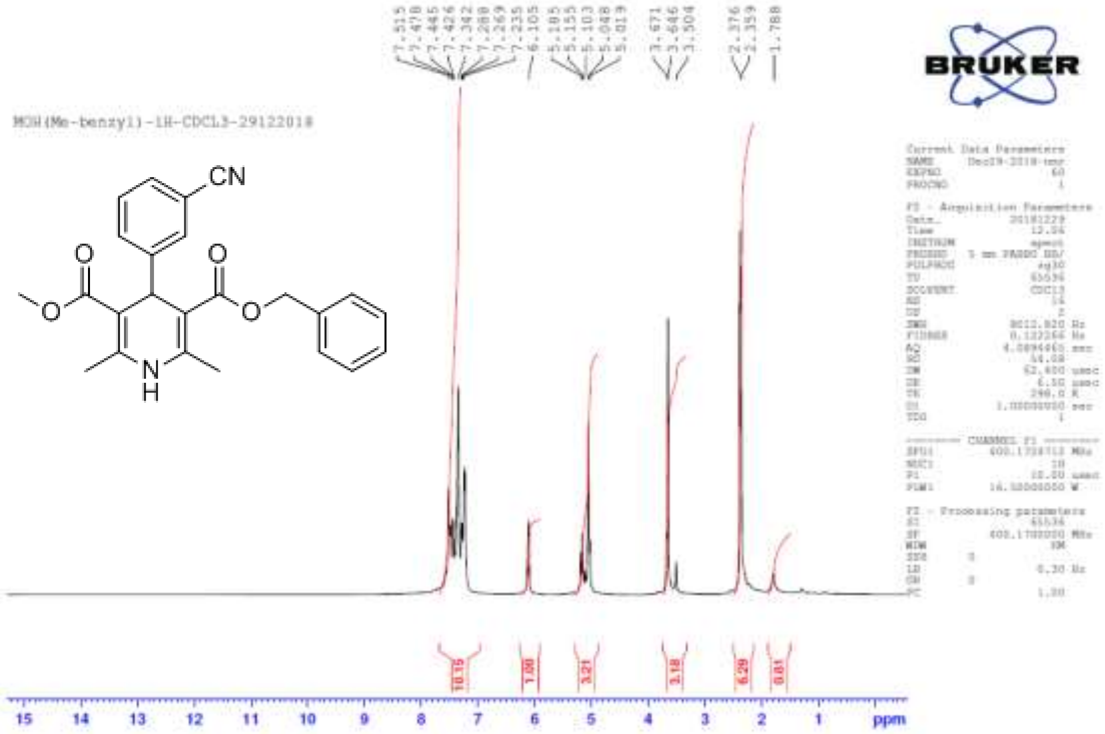

Figure S27. ^{1}H NMR (400 MHz, $CDCl_3$) spectrum of compound **2e**

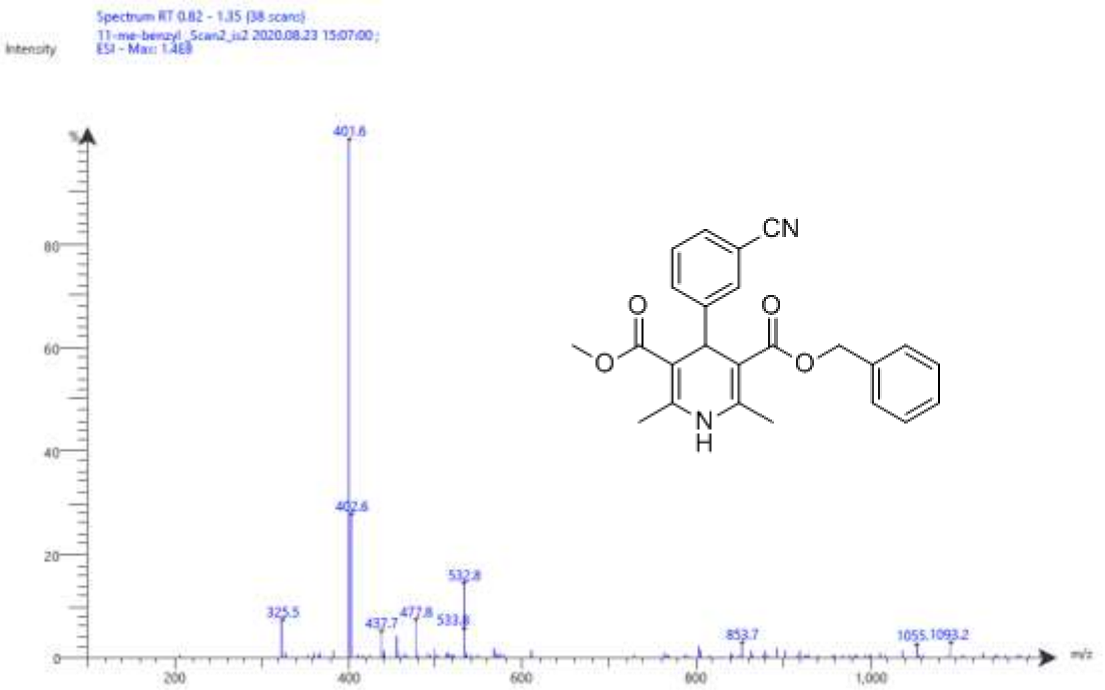

Figure S28. Negative ion ESI-MS spectrum of compound **2e**

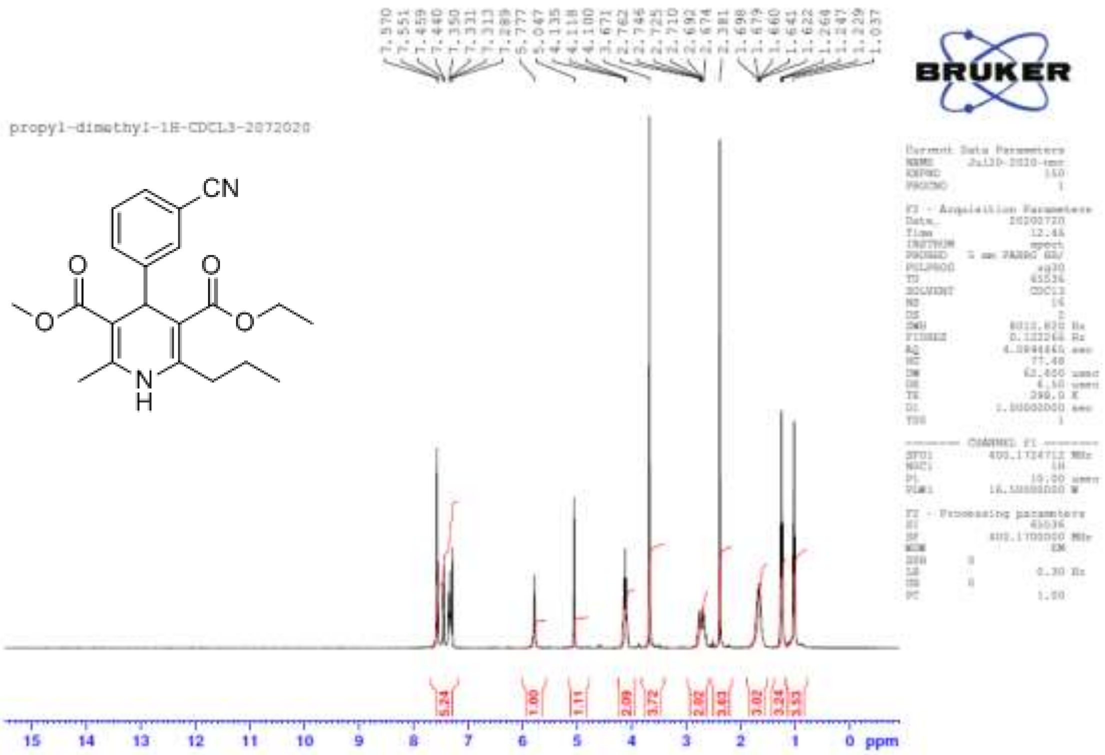

Figure S29. ^{1}H NMR (400 MHz, $CDCl_3$) spectrum of compound **2f**

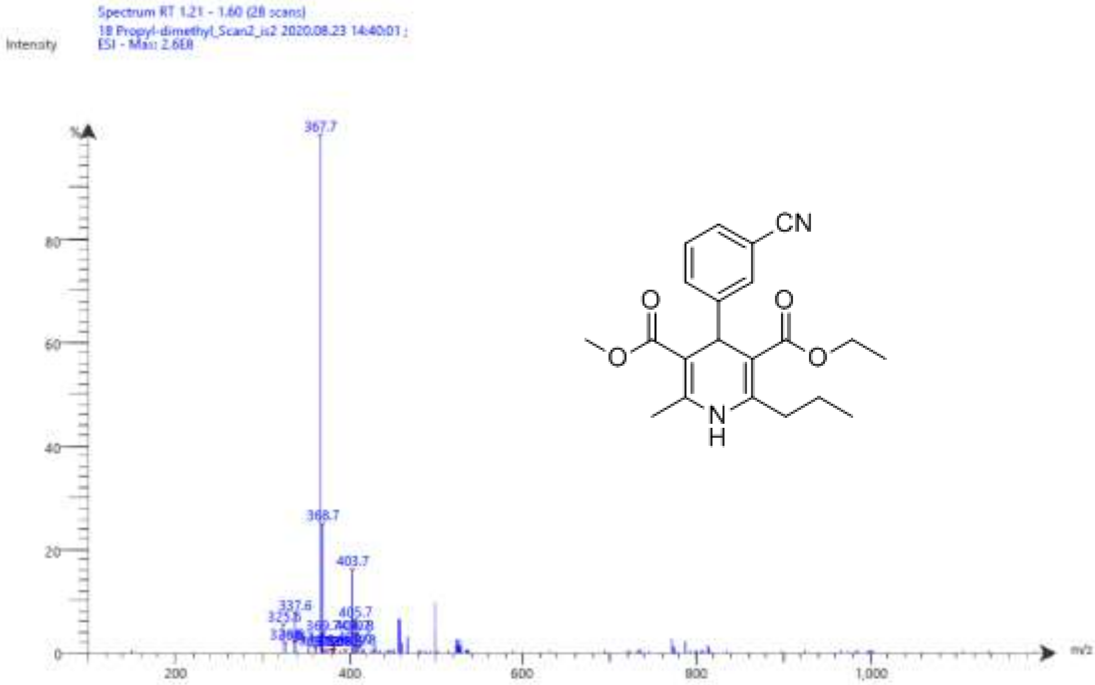

Figure S30. Negative ion ESI-MS spectrum of compound **2f**

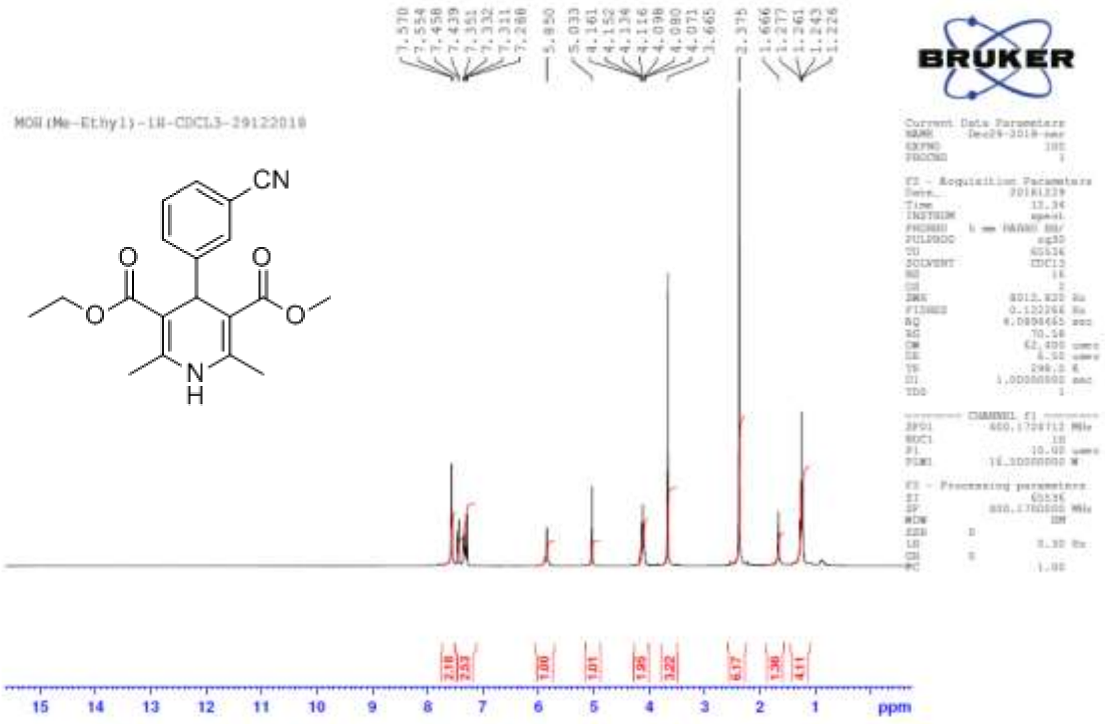

Figure S31. ^{1}H NMR (400 MHz, $CDCl_3$) spectrum of compound **2g**

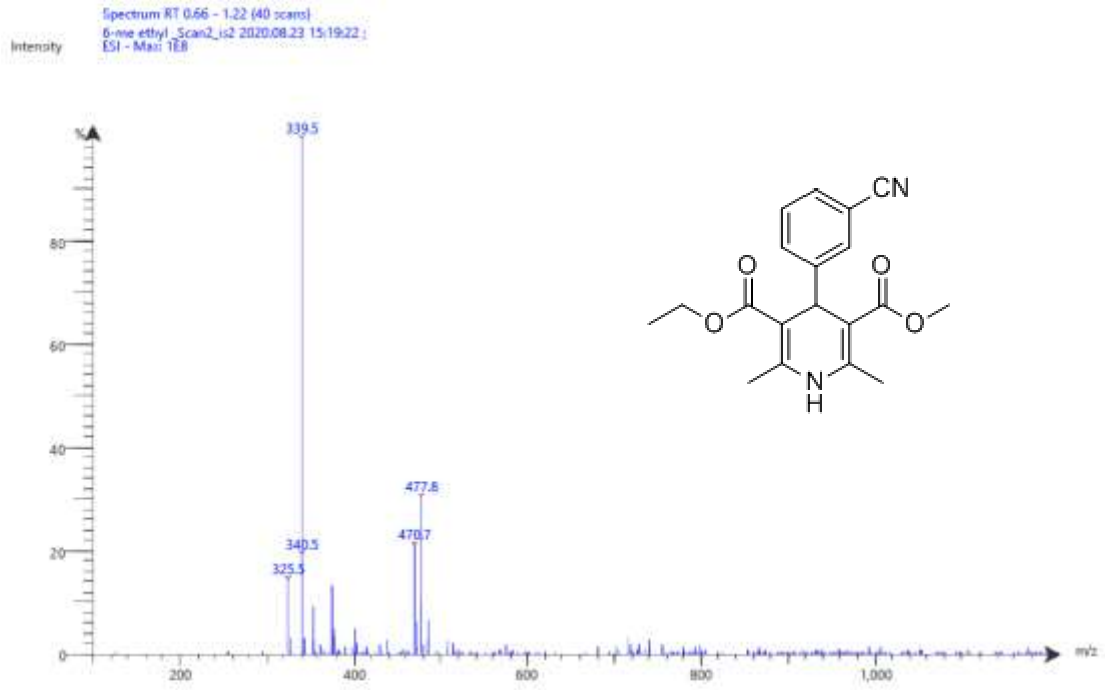

Figure S32. Negative ion ESI-MS spectrum of compound **2g**

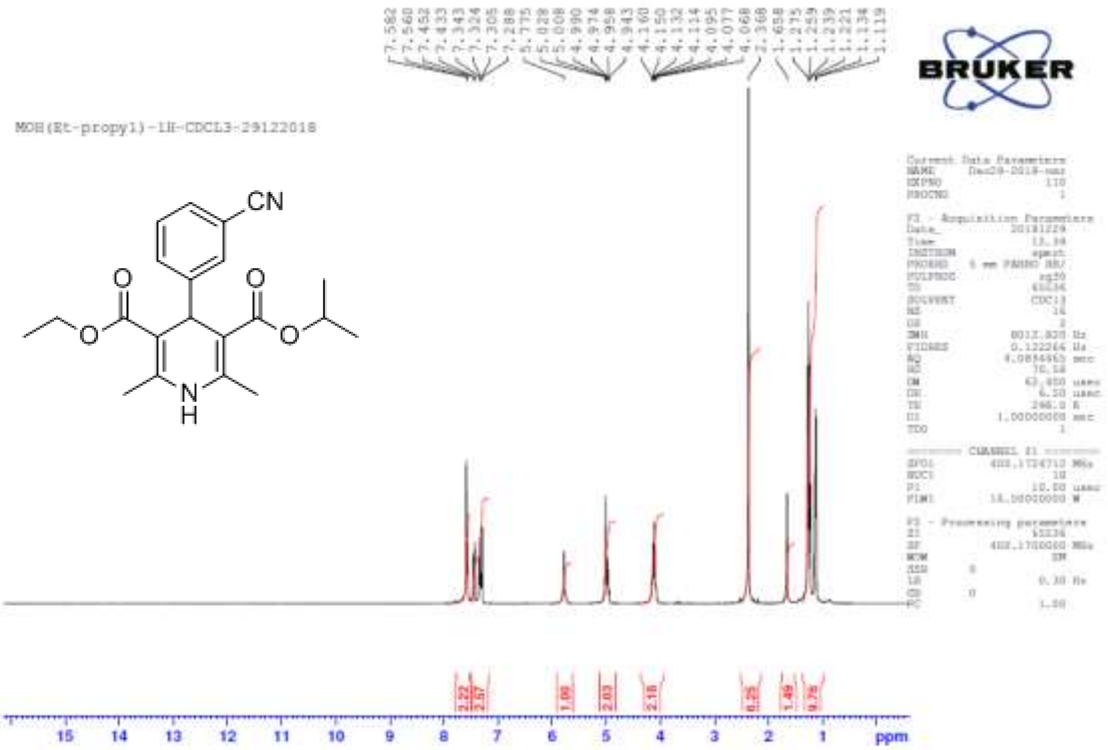

Figure S33. ^{1}H NMR (400 MHz, $CDCl_3$) spectrum of compound **2h**

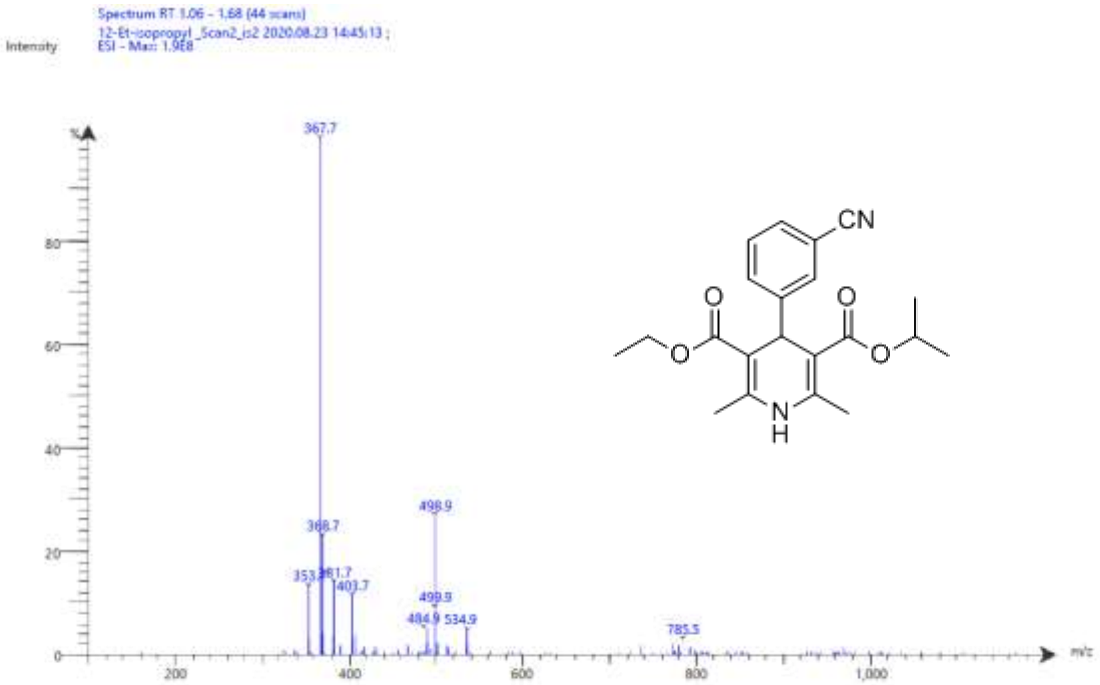

Figure S34. Negative ion ESI-MS spectrum of compound **2h**

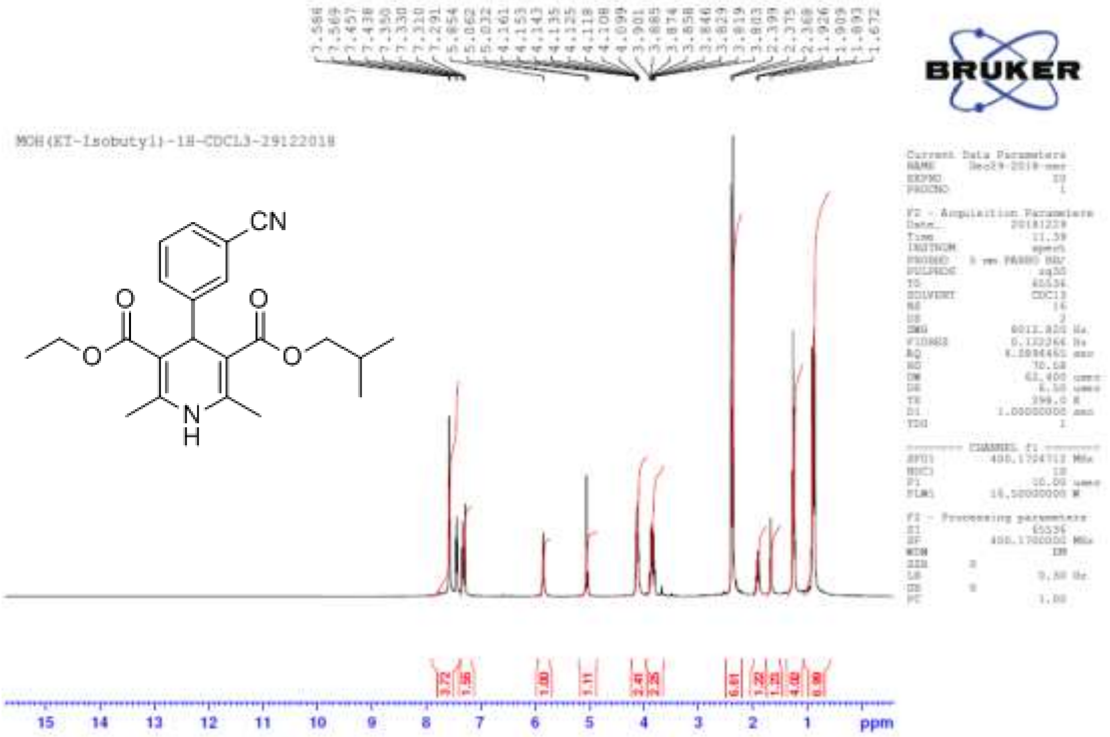

Figure S35. ^{1}H NMR (400 MHz, $CDCl_3$) spectrum of compound **2i**

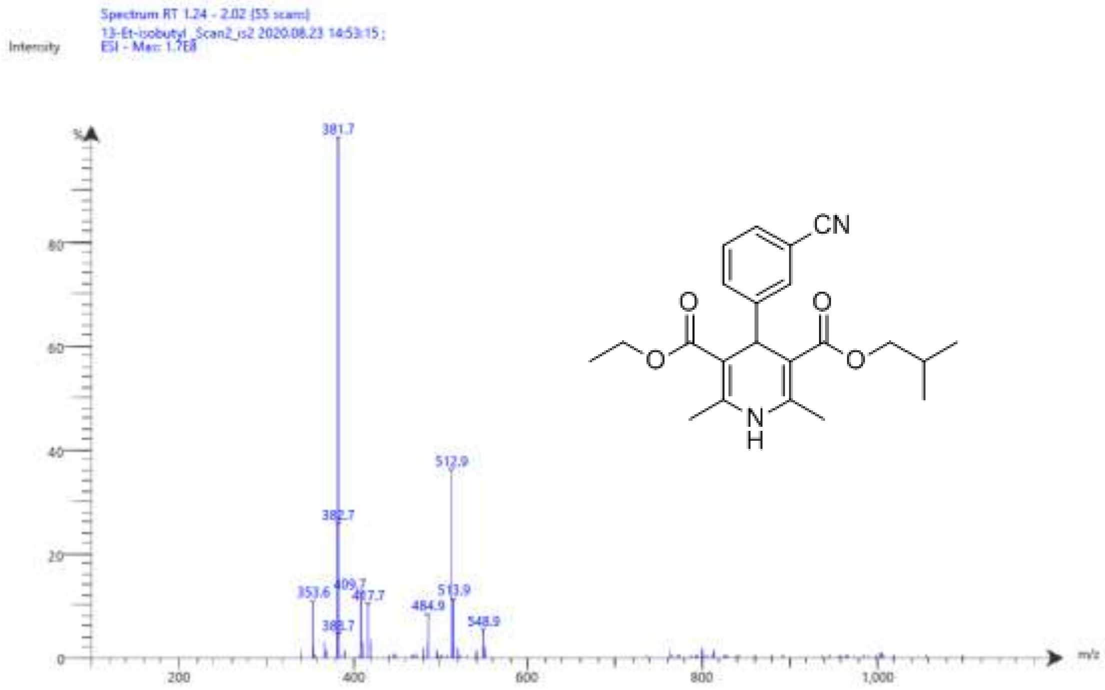

Figure S36. Negative ion ESI-MS spectrum of compound **2i**

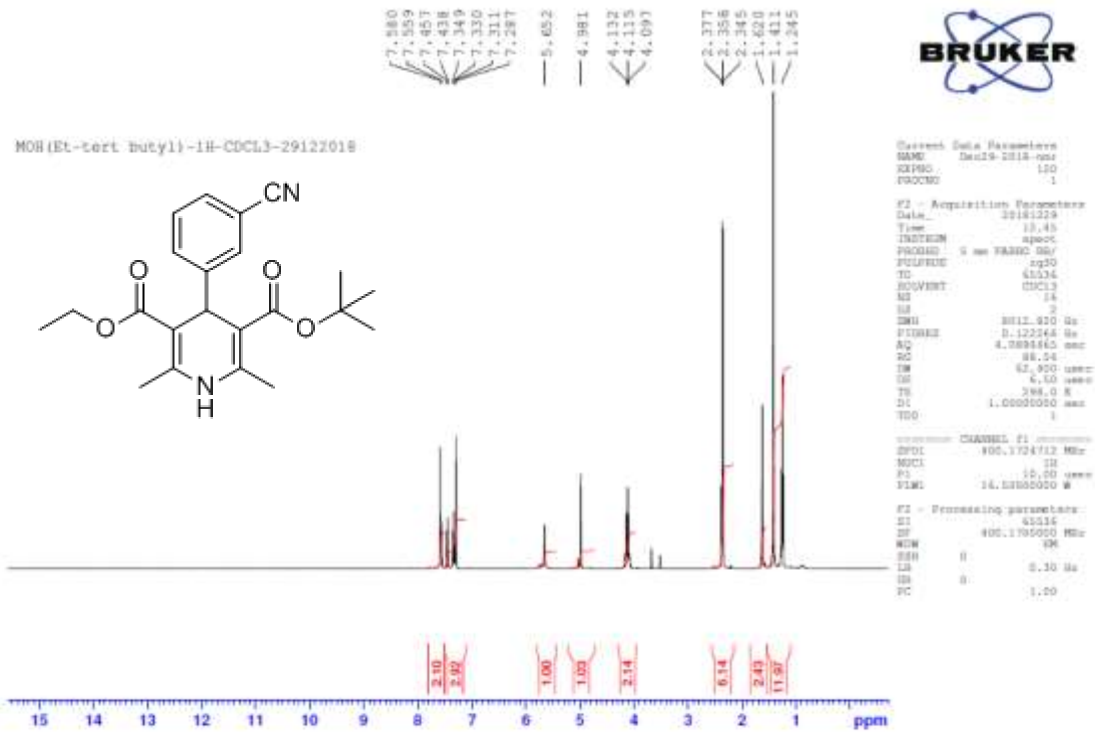

Figure S37. ^{1}H NMR (400 MHz, $CDCl_3$) spectrum of compound **2j**

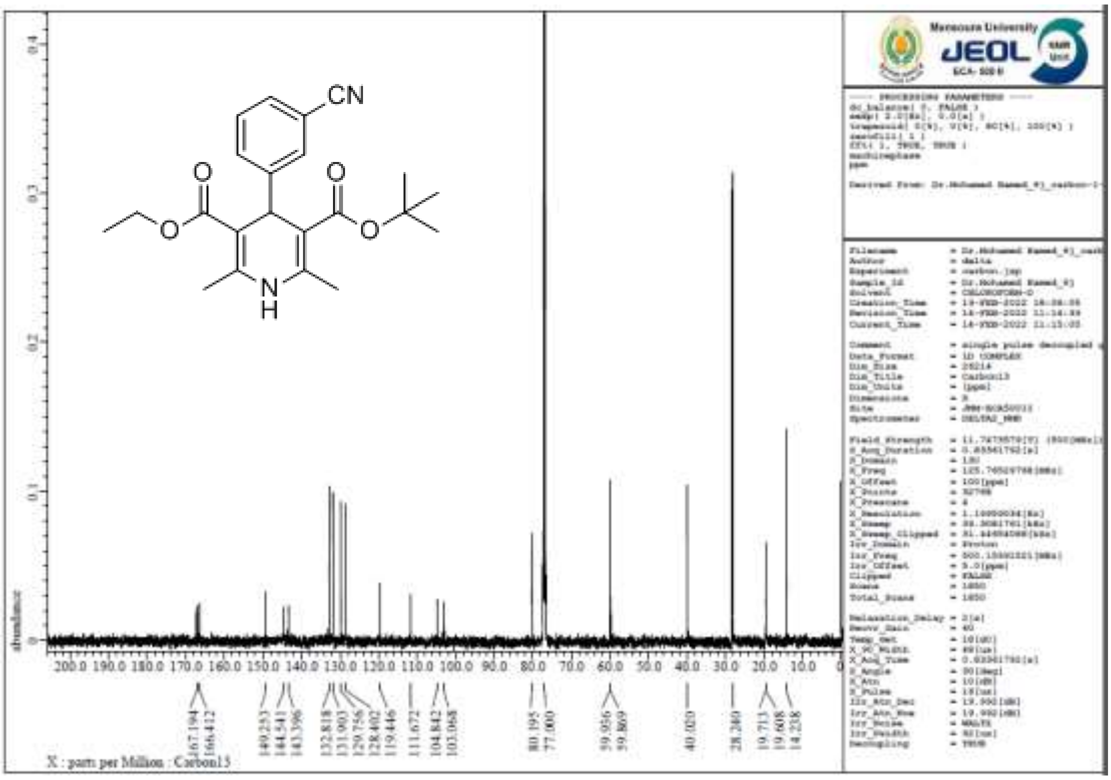

Figure S38. ^{13}C NMR (125 MHz, $CDCl_3$) spectrum of compound **2j**

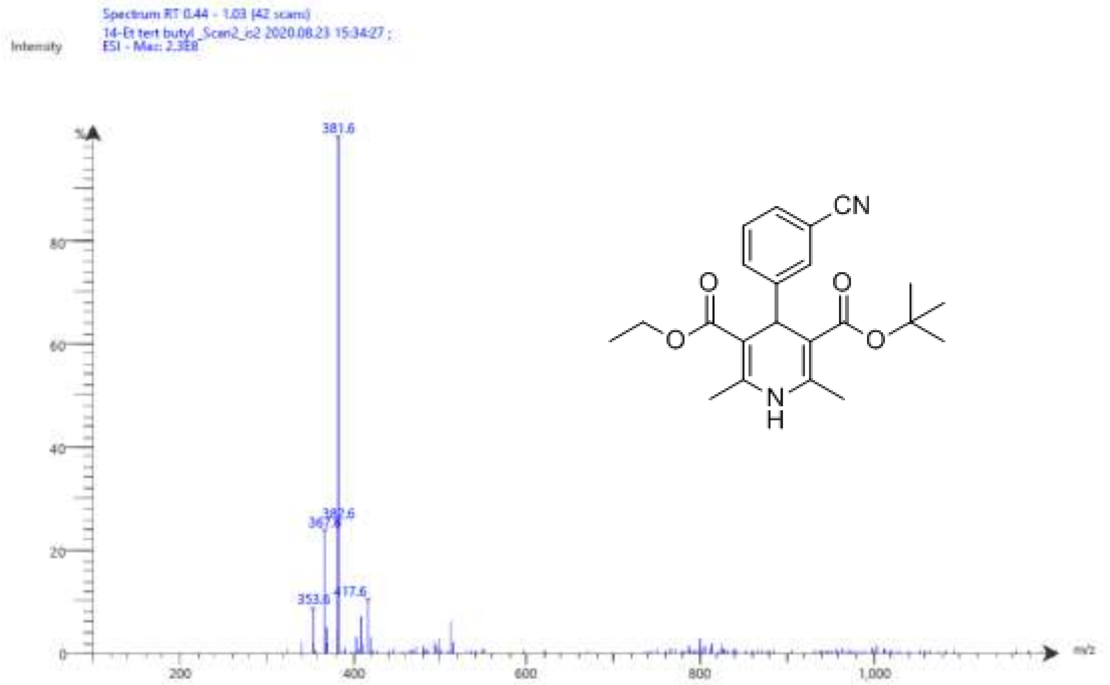

Figure S39. Negative ion ESI-MS spectrum of compound **2j**

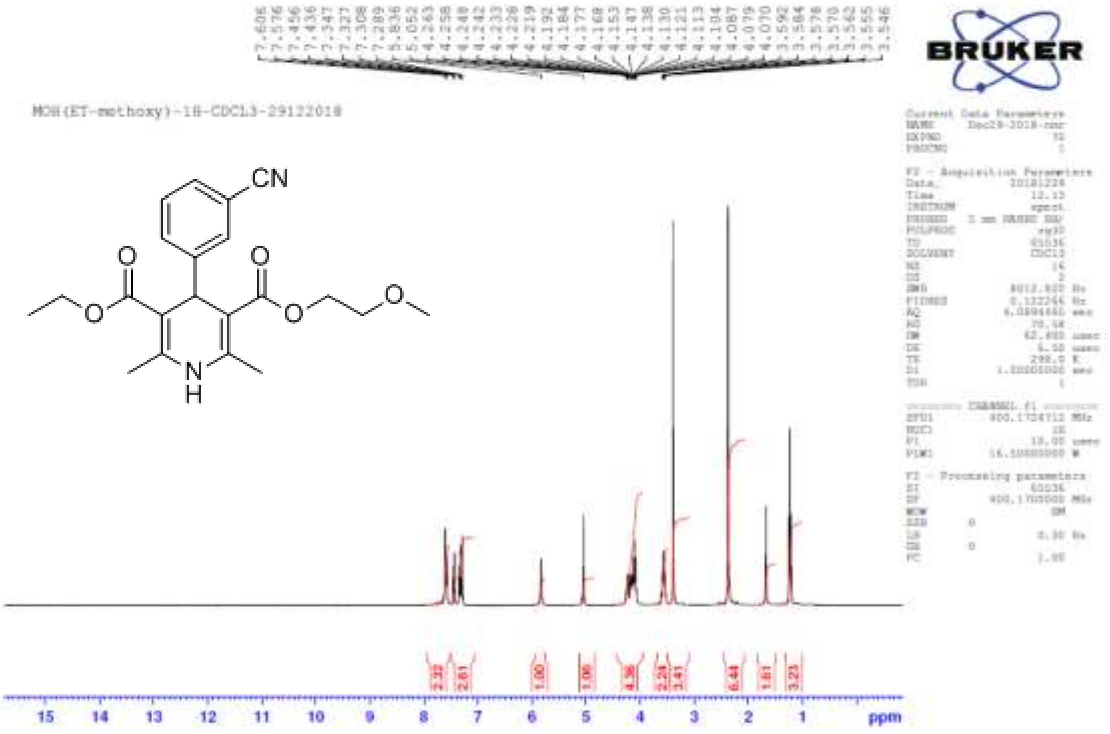

Figure S40. ^{1}H NMR (400 MHz, $CDCl_3$) spectrum of compound **2k**

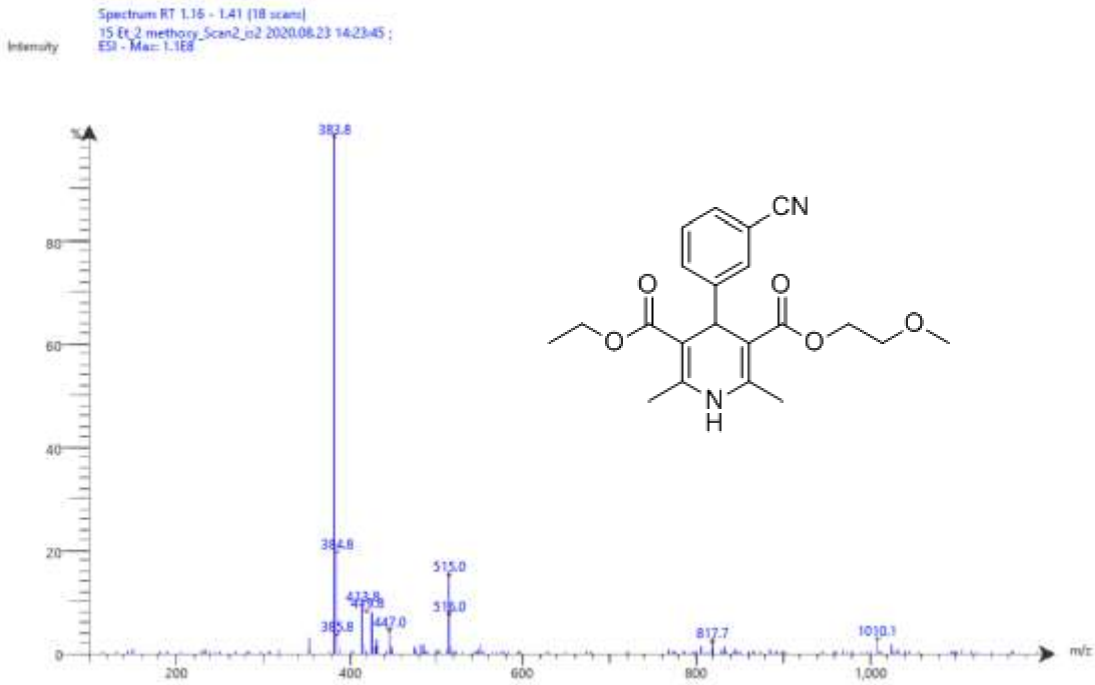

Figure S41. Negative ion ESI-MS spectrum of compound **2k**

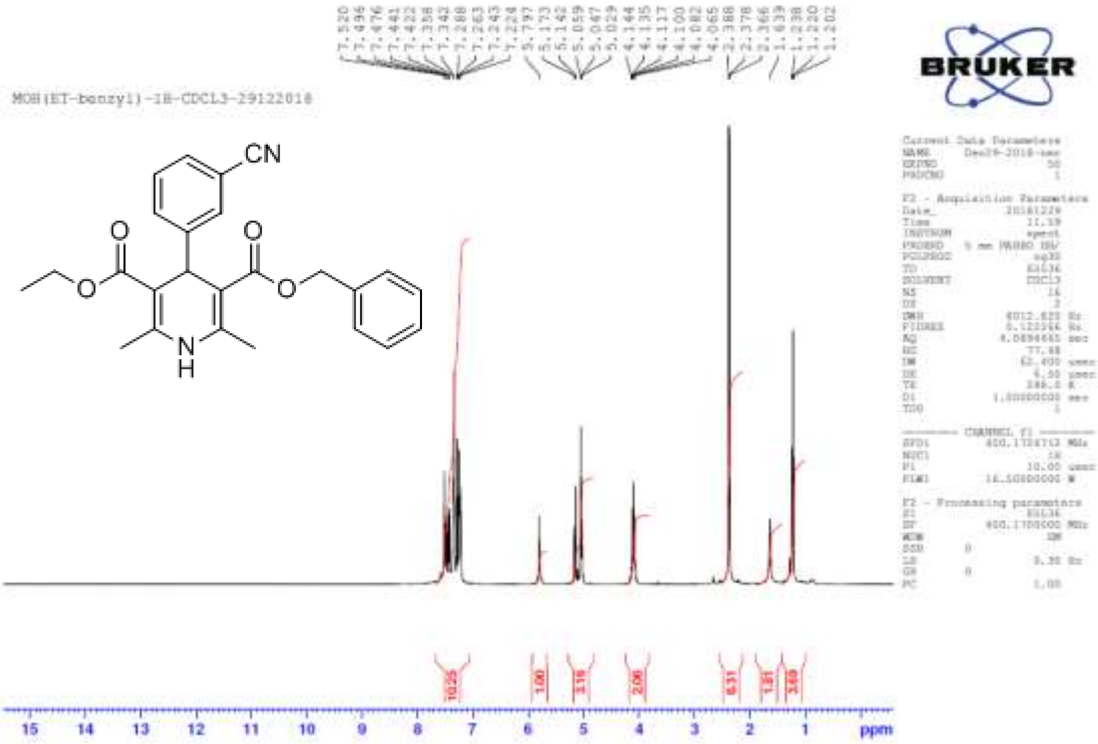

Figure S42. ^{1}H NMR (400 MHz, $CDCl_3$) spectrum of compound **2l**

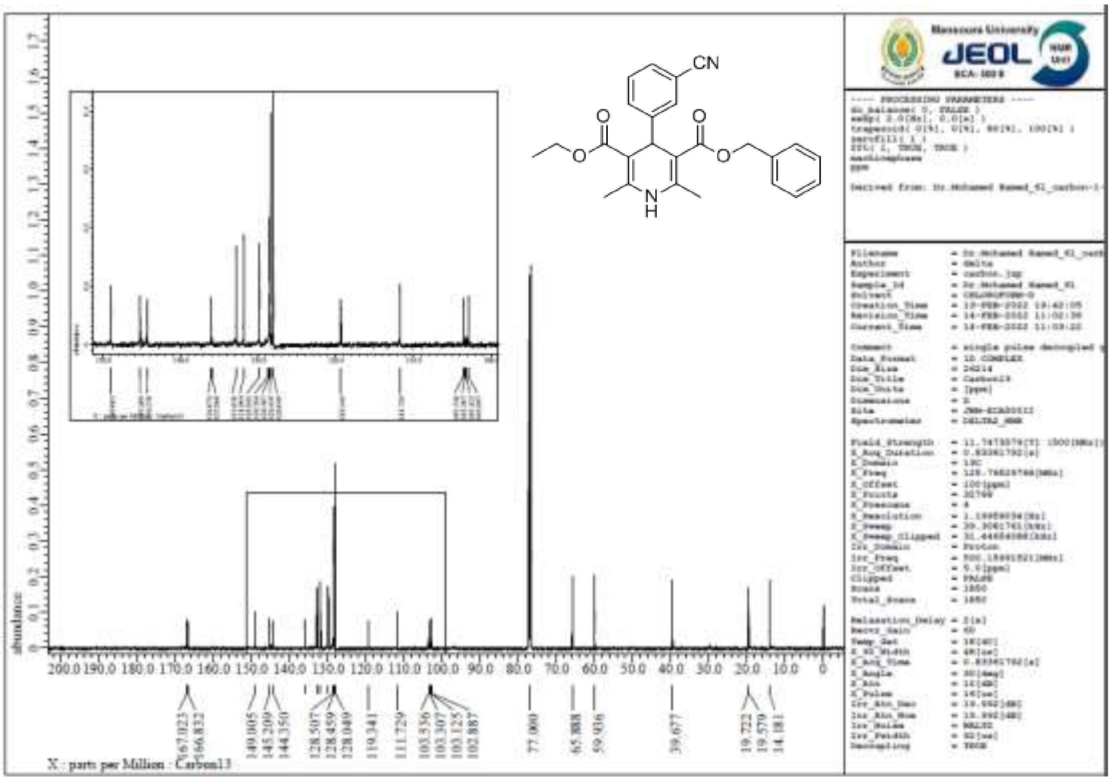

Figure S43. ^{13}C NMR (125 MHz, $CDCl_3$) spectrum of compound **2l**

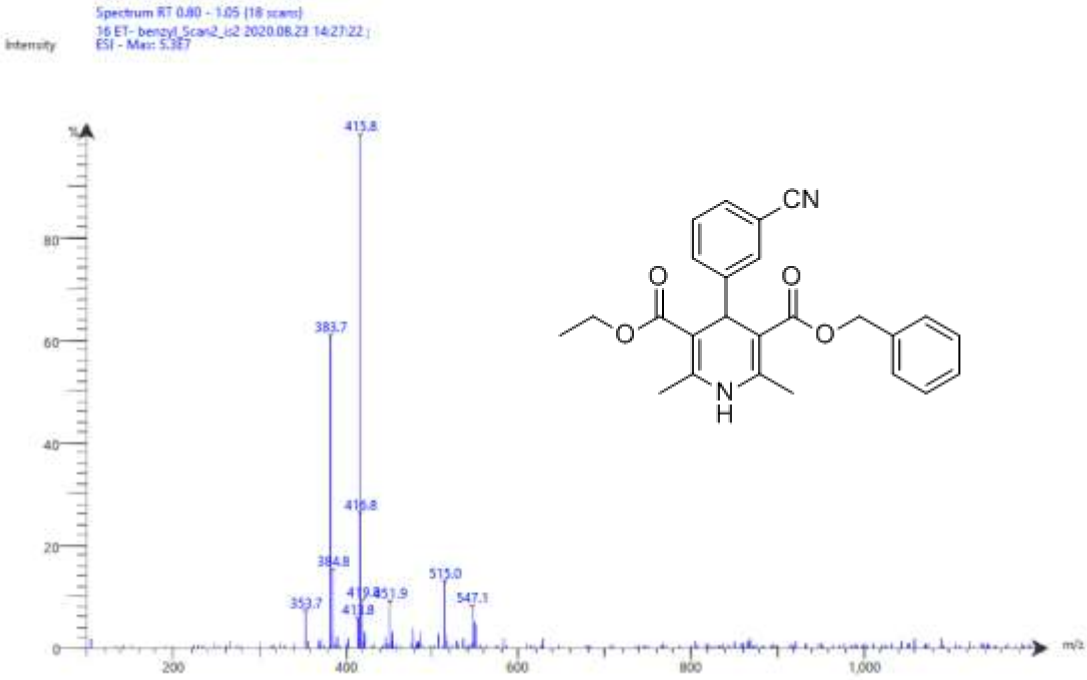

Figure S44. Negative ion ESI-MS spectrum of compound **2l**

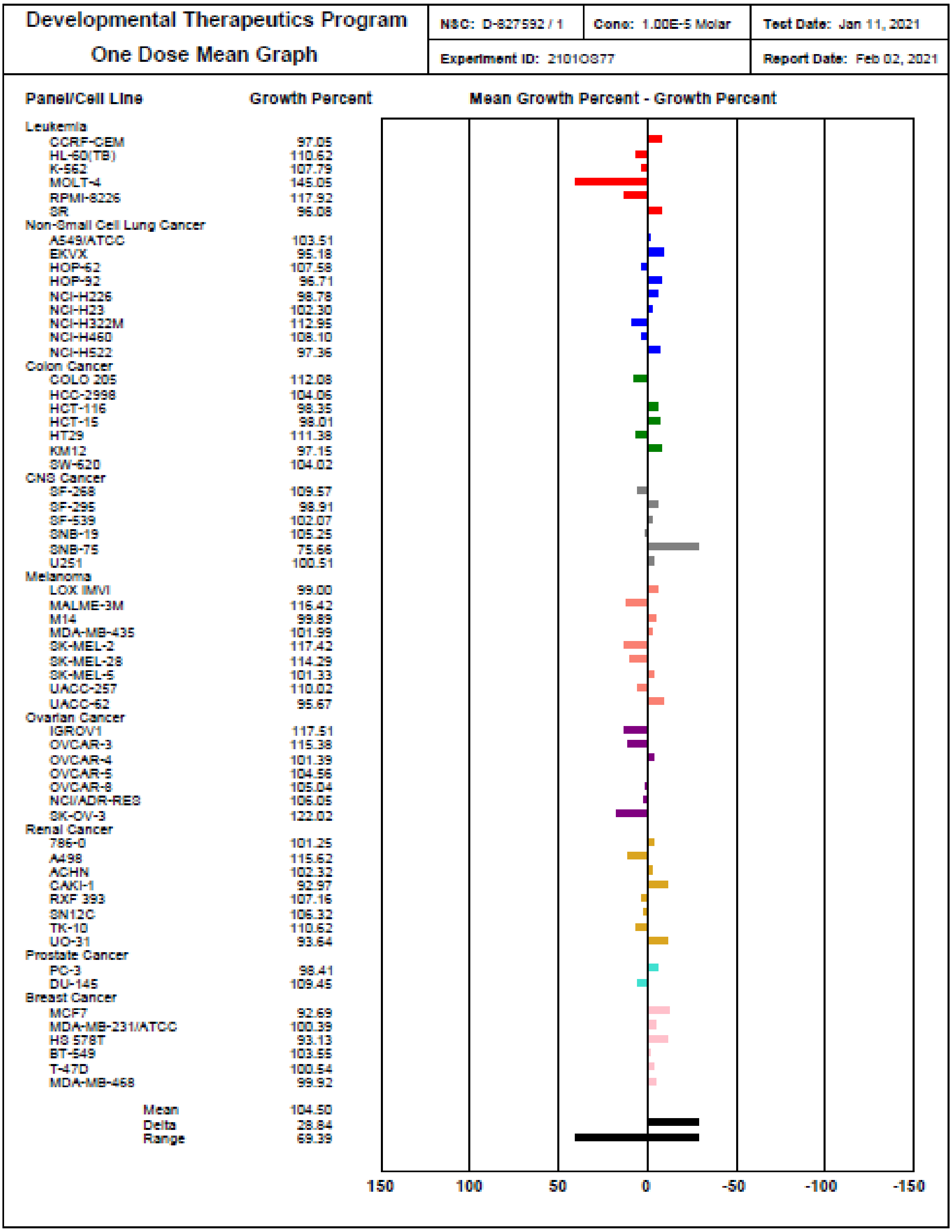

Figure S45. One dose mean graph for compound **1a** (NSC 827592) at 10 µM concentration

Developmental Therapeutics Program One Dose Mean Graph	NSC: D-827593 / 1	Conc: 1.00E-5 Molar	Test Date: Jan 11, 2021
	Experiment ID: 2101OS77		Report Date: Feb 02, 2021

Panel/Cell Line	Growth Percent
**Leukemia**	
CCRF-CEM	96.91
HL-60(TB)	86.15
K-562	56.51
MOLT-4	97.85
RPMI-8226	94.58
SR	51.06
**Non-Small Cell Lung Cancer**	
A549/ATCC	96.21
EKVX	95.07
HOP-62	93.07
HOP-92	89.70
NCI-H226	91.82
NCI-H23	100.55
NCI-H322M	91.47
NCI-H460	109.24
NCI-H522	87.40
**Colon Cancer**	
COLO 205	100.89
HCC-2998	102.02
HCT-116	88.16
HCT-15	76.40
HT29	100.32
KM12	72.05
SW-620	98.98
**CNS Cancer**	
SF-268	96.02
SF-295	92.85
SF-539	101.32
SNB-19	94.70
SNB-75	61.67
U251	91.46
**Melanoma**	
LOX IMVI	92.41
MALME-3M	97.76
M14	100.30
MDA-MB-435	57.11
SK-MEL-2	109.65
SK-MEL-28	105.03
SK-MEL-5	100.62
UACC-257	99.04
UACC-62	79.51
**Ovarian Cancer**	
IGROV1	91.53
OVCAR-3	108.56
OVCAR-4	99.28
OVCAR-5	94.74
OVCAR-8	97.33
NCI/ADR-RES	92.76
SK-OV-3	95.05
**Renal Cancer**	
786-0	92.60
A498	105.81
ACHN	94.63
CAKI-1	68.26
RXF 393	89.61
SN12C	107.49
TK-10	99.96
UO-31	86.31
**Prostate Cancer**	
PC-3	79.23
DU-145	111.27
**Breast Cancer**	
MCF7	84.95
MDA-MB-231/ATCC	94.73
HS 578T	83.88
BT-549	103.15
T-47D	76.51
MDA-MB-468	77.51
Mean	91.52
Delta	40.46
Range	60.21

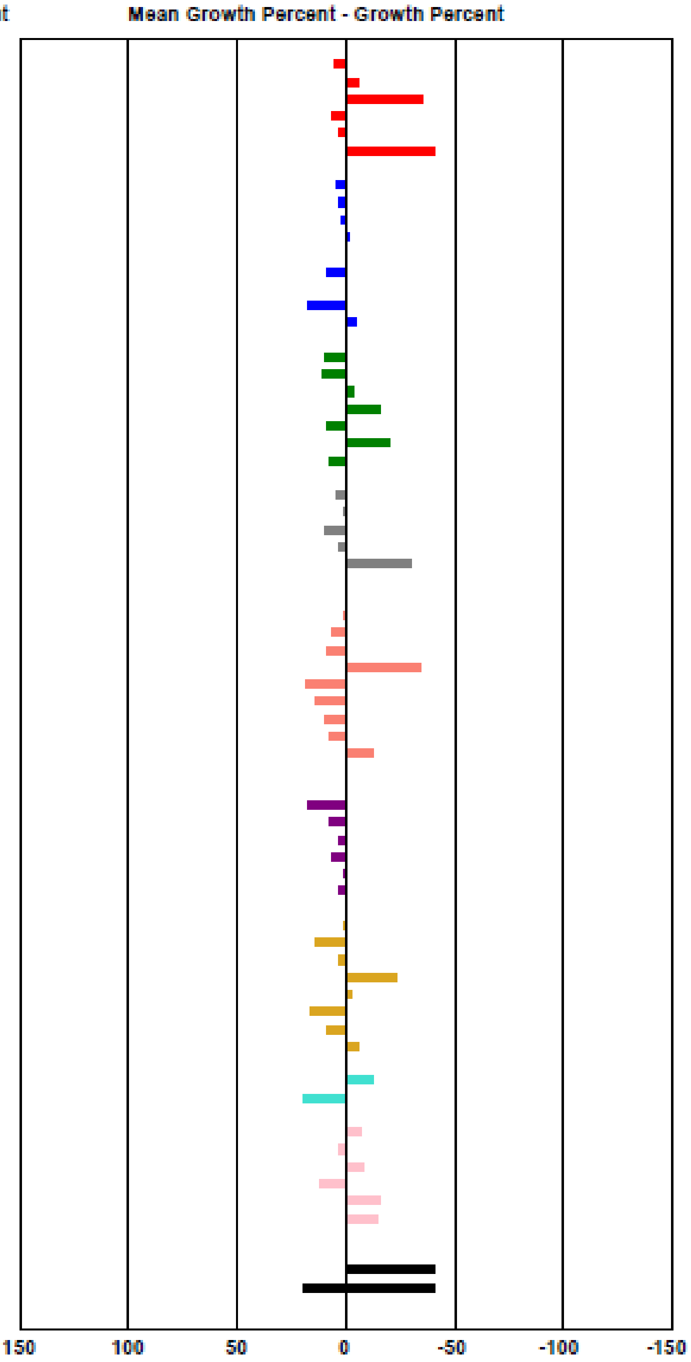

Figure S46. One dose mean graph for compound **1b** (NSC 827593) at 10 µM concentration

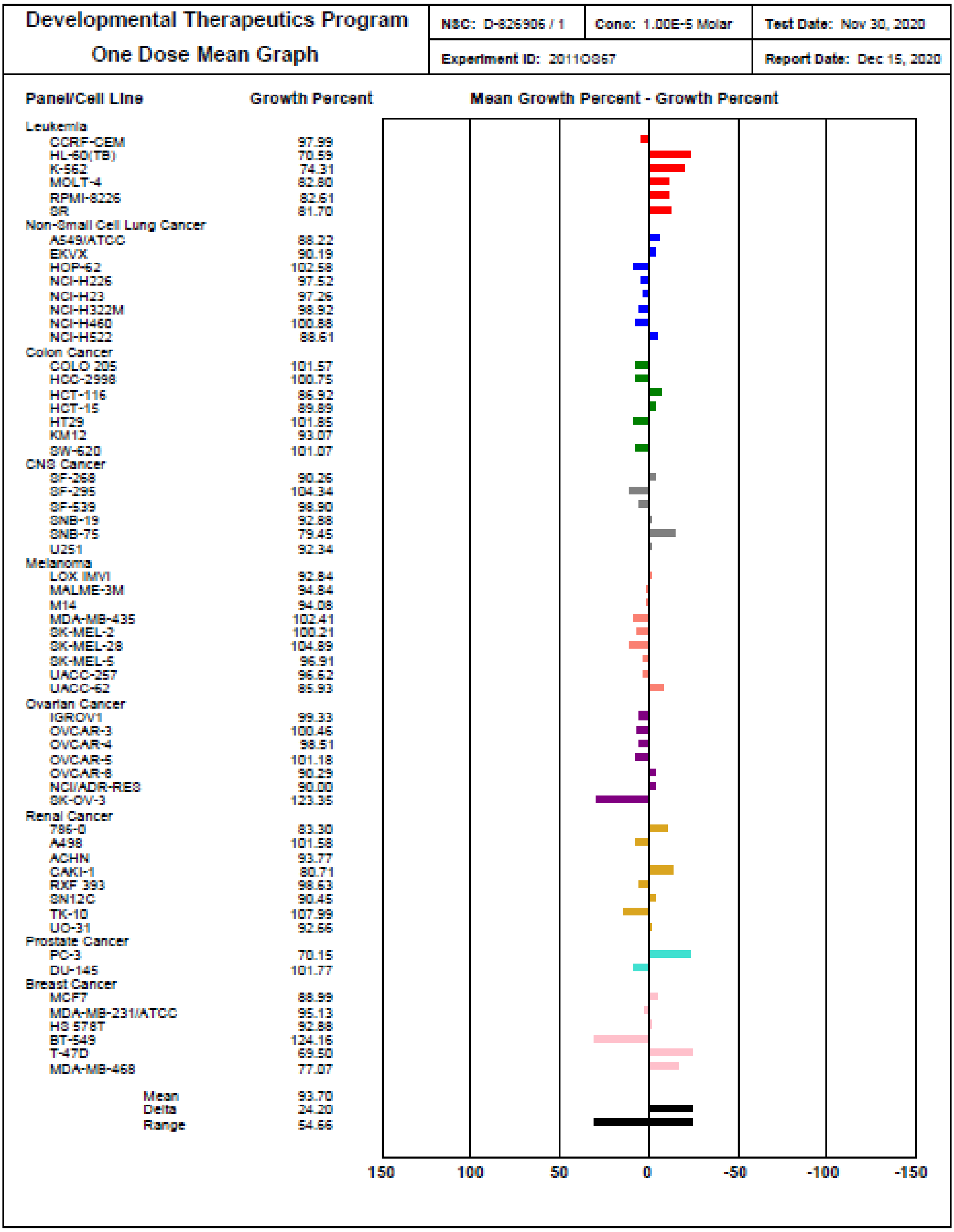

Figure S47. One dose mean graph for compound **1c** (NSC 826906) at 10 μM concentration

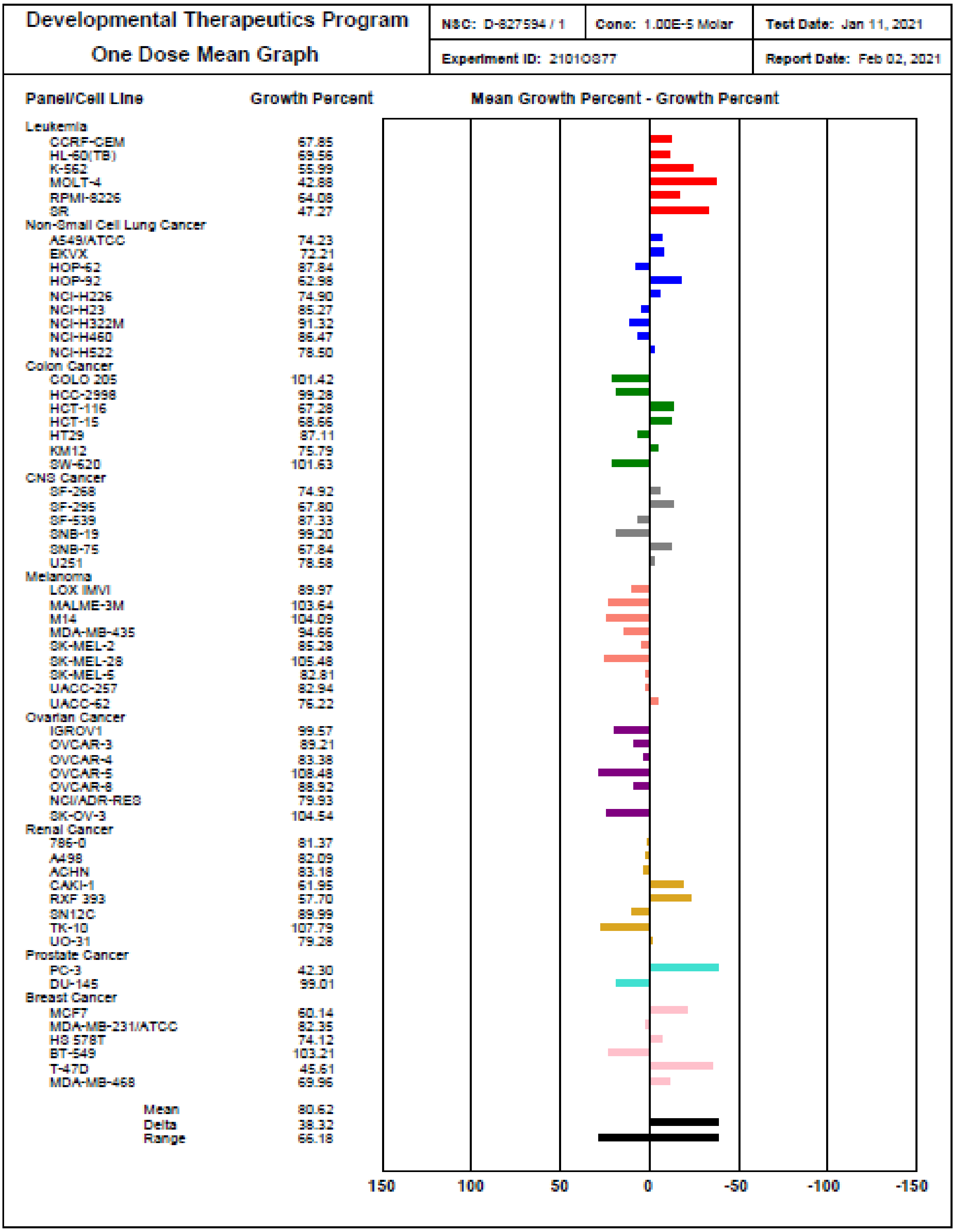

Figure S48. One dose mean graph for compound **1d** (NSC 827594) at 10 µM concentration

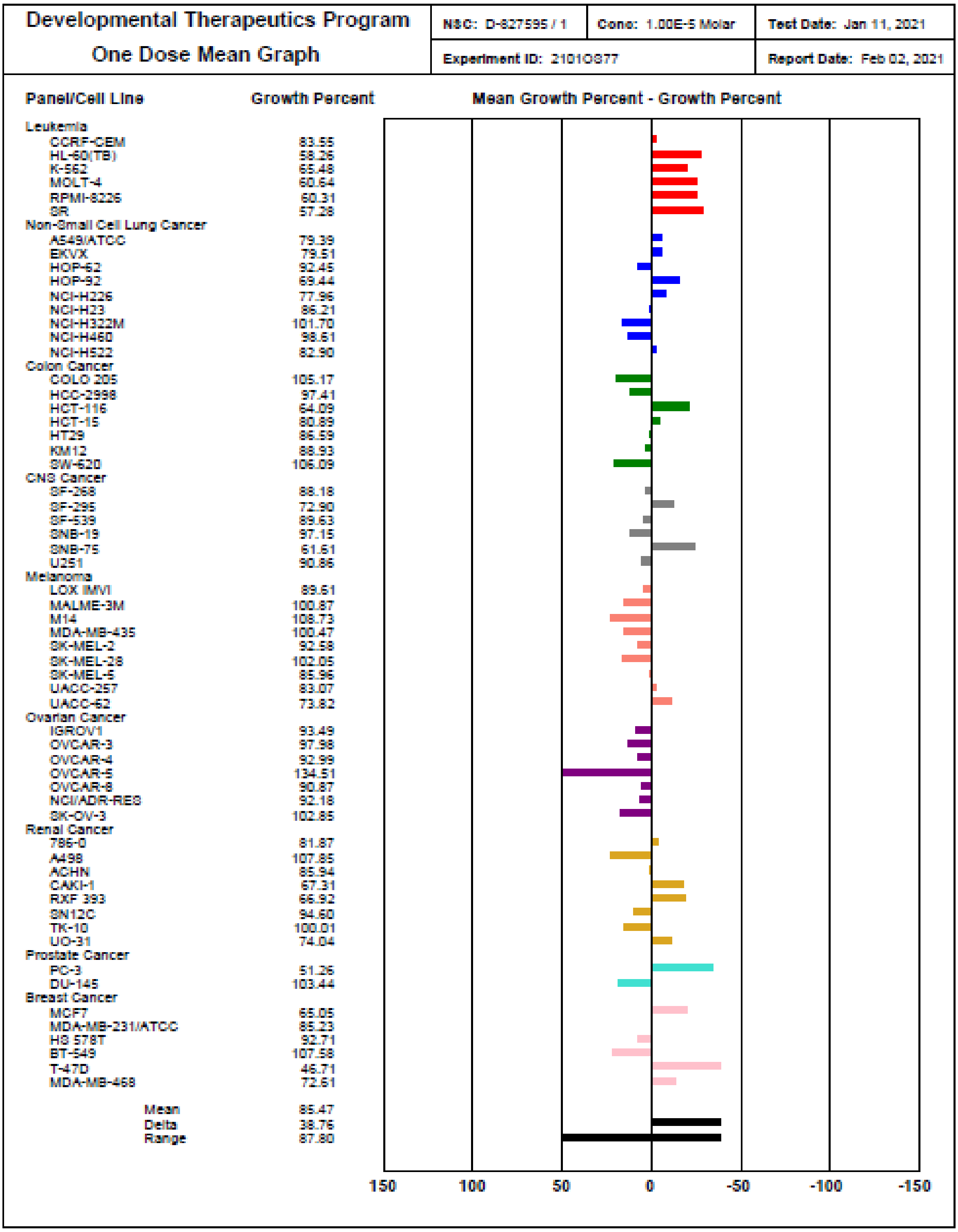

Figure S49. One dose mean graph for compound **1e** (NSC 827595) at 10 μM concentration

Developmental Therapeutics Program One Dose Mean Graph	NSC: D-826907 / 1	Conc: 1.00E-5 Molar	Test Date: Nov 30, 2020
	Experiment ID: 2011OS67		Report Date: Dec 15, 2020

Panel/Cell Line	Growth Percent
**Leukemia**	
CCRF-CEM	104.27
HL-60(TB)	89.70
K-562	94.53
MOLT-4	101.91
RPMI-8226	108.85
SR	97.36
**Non-Small Cell Lung Cancer**	
A549/ATCC	99.92
EKVX	98.38
HOP-62	98.64
HOP-92	102.77
NCI-H226	97.14
NCI-H23	101.83
NCI-H322M	105.98
NCI-H460	101.50
NCI-H522	93.34
**Colon Cancer**	
COLO 205	118.47
HCC-2998	102.36
HCT-116	105.92
HCT-15	102.82
HT29	107.37
KM12	100.05
SW-620	101.27
**CNS Cancer**	
SF-268	95.31
SF-295	102.84
SF-539	99.86
SNB-19	105.45
SNB-75	85.43
U251	95.87
**Melanoma**	
LOX IMVI	98.73
MALME-3M	103.98
M14	99.09
MDA-MB-435	105.30
SK-MEL-2	104.49
SK-MEL-28	106.89
SK-MEL-5	100.63
UACC-257	101.82
UACC-62	100.40
**Ovarian Cancer**	
IGROV1	105.64
OVCAR-3	102.44
OVCAR-4	102.40
OVCAR-5	103.82
OVCAR-8	104.00
NCI/ADR-RES	101.23
SK-OV-3	119.56
**Renal Cancer**	
786-0	94.25
A498	100.31
ACHN	103.57
CAKI-1	90.21
RXF 393	102.87
SN12C	100.24
TK-10	97.81
UO-31	95.70
**Prostate Cancer**	
PC-3	99.01
DU-145	105.63
**Breast Cancer**	
MCF7	96.57
MDA-MB-231/ATCC	105.92
HS 578T	95.98
BT-549	87.84
T-47D	92.90
MDA-MB-468	86.43
Mean	100.58
Delta	15.15
Range	34.13

Mean Growth Percent - Growth Percent

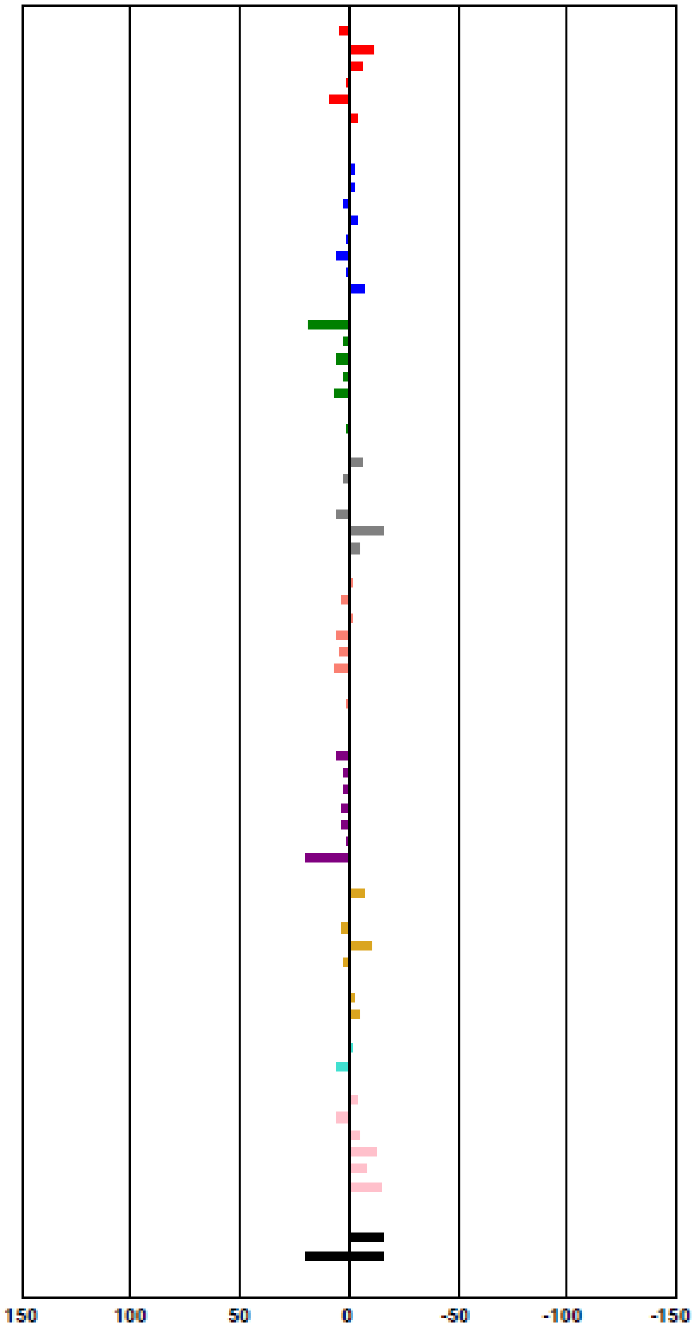

Figure S50. One dose mean graph for compound **1f** (NSC 826907) at 10 µM concentration

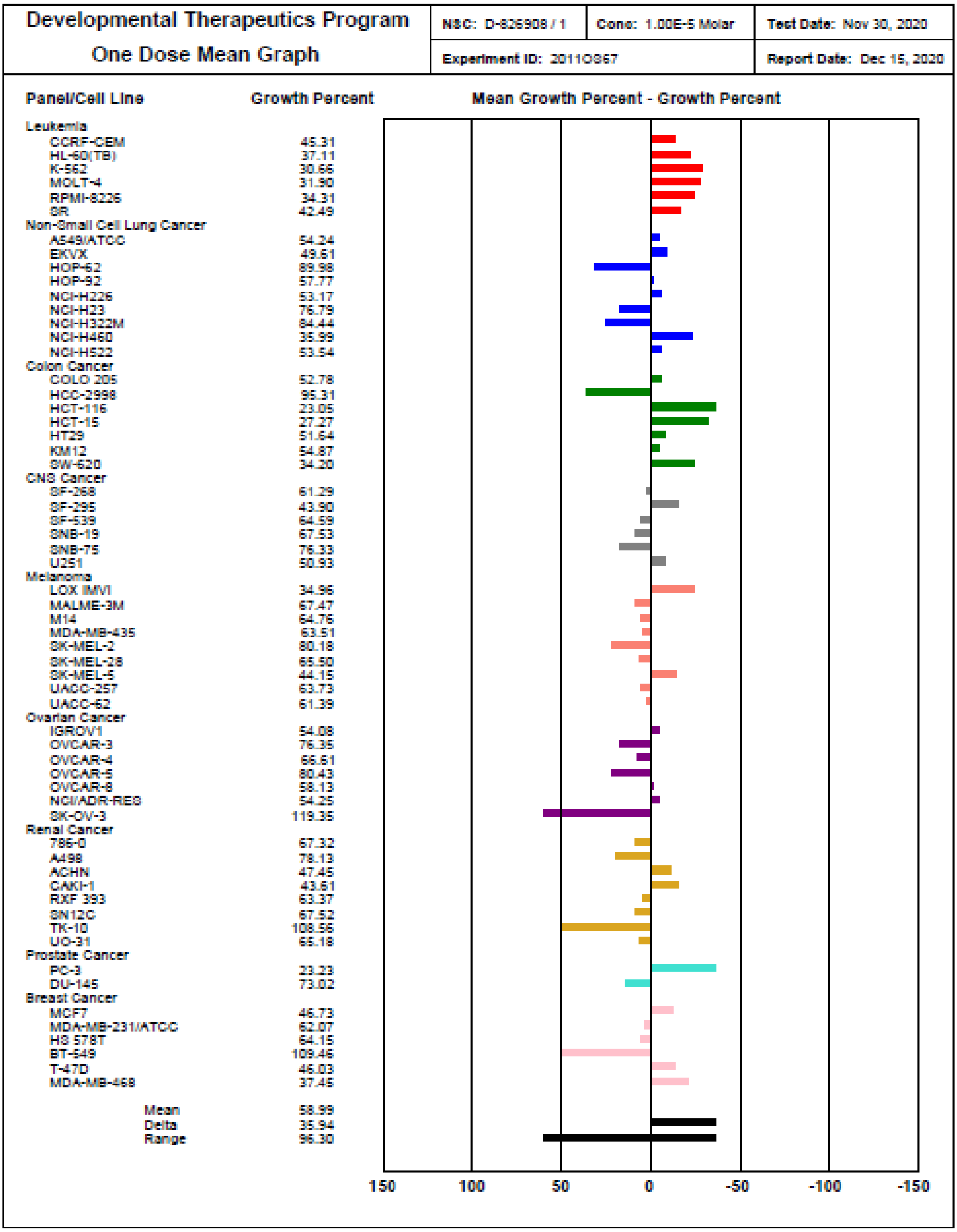

Figure S51. One dose mean graph for compound **1g** (NSC 826908) at 10 µM concentration

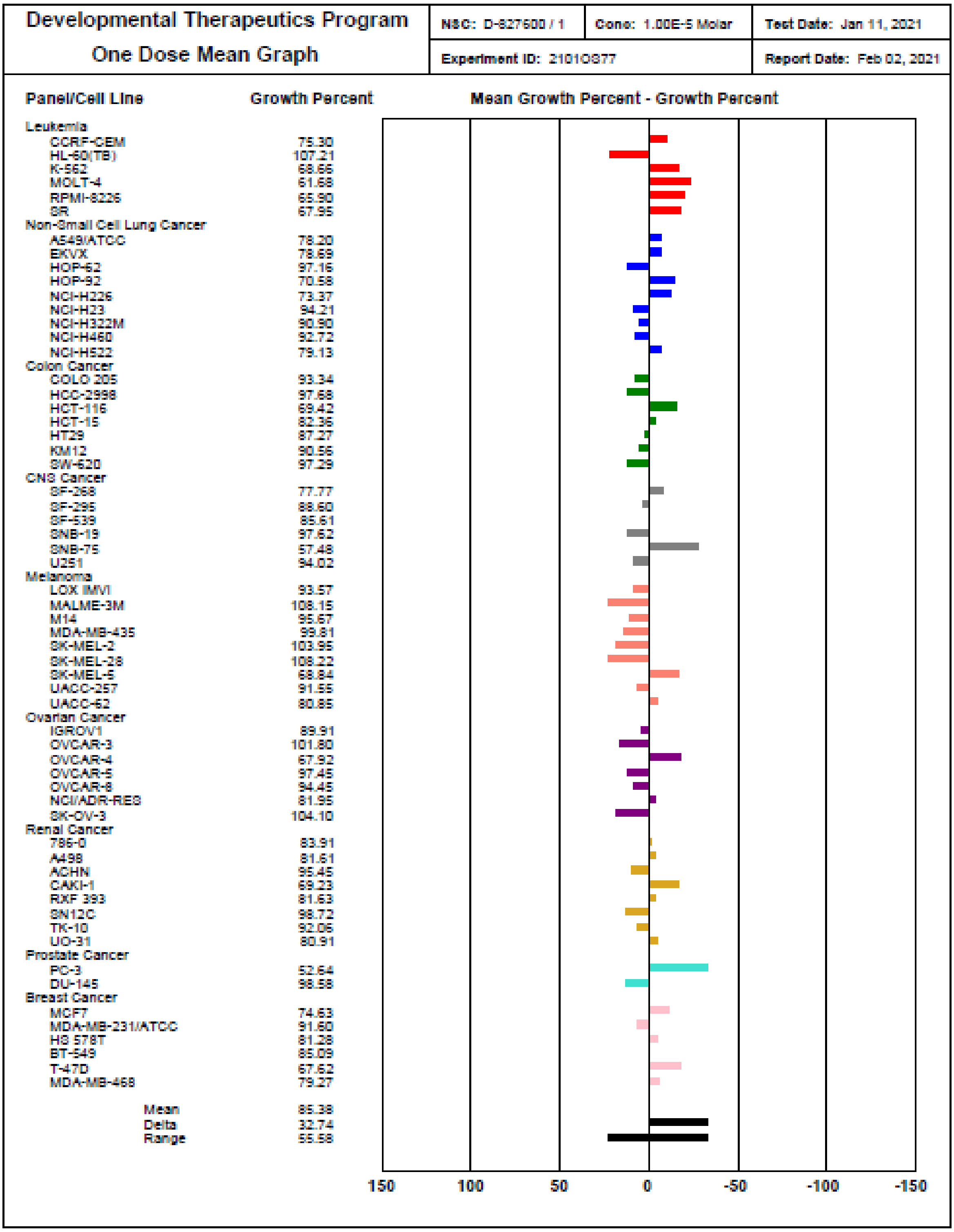

Figure S52. One dose mean graph for compound **1h** (NSC 827600) at 10 µM concentration

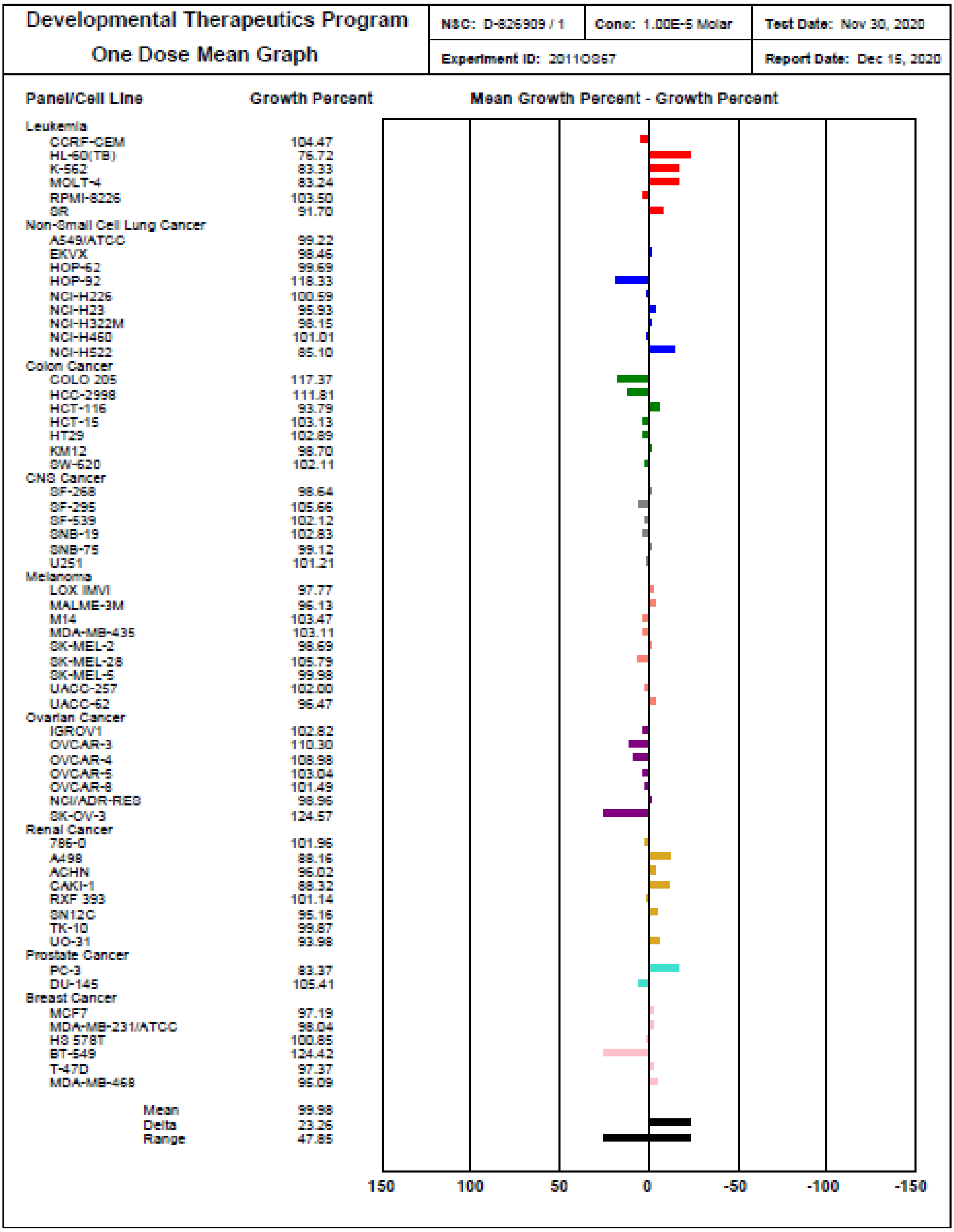

Figure S53. One dose mean graph for compound **2a** (NSC 826909) at 10 µM concentration

Developmental Therapeutics Program One Dose Mean Graph	NSC: D-827597 / 1	Conc: 1.00E-5 Molar	Test Date: Jan 11, 2021
	Experiment ID: 2101OS77		Report Date: Feb 02, 2021

Panel/Cell Line	Growth Percent
Leukemia	
CCRF-CEM	92.56
HL-60(TB)	81.56
K-562	84.52
MOLT-4	83.28
RPMI-8226	90.51
SR	70.40
Non-Small Cell Lung Cancer	
A549/ATCC	90.94
EKVX	87.02
HOP-62	95.54
HOP-92	82.18
NCI-H226	89.08
NCI-H23	96.19
NCI-H322M	102.98
NCI-H460	105.97
NCI-H522	84.71
Colon Cancer	
COLO 205	115.58
HCC-2998	101.92
HCT-116	81.96
HCT-15	90.45
HT29	100.53
KM12	94.24
SW-620	107.07
CNS Cancer	
SF-268	94.40
SF-295	81.82
SF-539	98.48
SNB-19	101.17
SNB-75	77.18
U251	93.94
Melanoma	
LOX IMVI	93.74
MALME-3M	106.76
M14	110.95
MDA-MB-435	99.53
SK-MEL-2	103.84
SK-MEL-28	114.76
SK-MEL-5	95.08
UACC-257	100.20
UACC-62	78.93
Ovarian Cancer	
IGROV1	87.10
OVCAR-3	107.86
OVCAR-4	91.80
OVCAR-5	108.36
OVCAR-8	93.69
NCI/ADR-RES	95.91
SK-OV-3	104.54
Renal Cancer	
786-0	92.63
A498	103.13
ACHN	99.88
CAKI-1	71.55
RXF 393	92.69
SN12C	107.38
TK-10	110.44
UO-31	88.75
Prostate Cancer	
PC-3	77.05
DU-145	109.57
Breast Cancer	
MCF7	84.33
MDA-MB-231/ATCC	94.11
HS 578T	85.19
BT-549	102.46
T-47D	74.51
MDA-MB-468	92.85
Mean	94.26
Delta	23.86
Range	45.18

Mean Growth Percent - Growth Percent

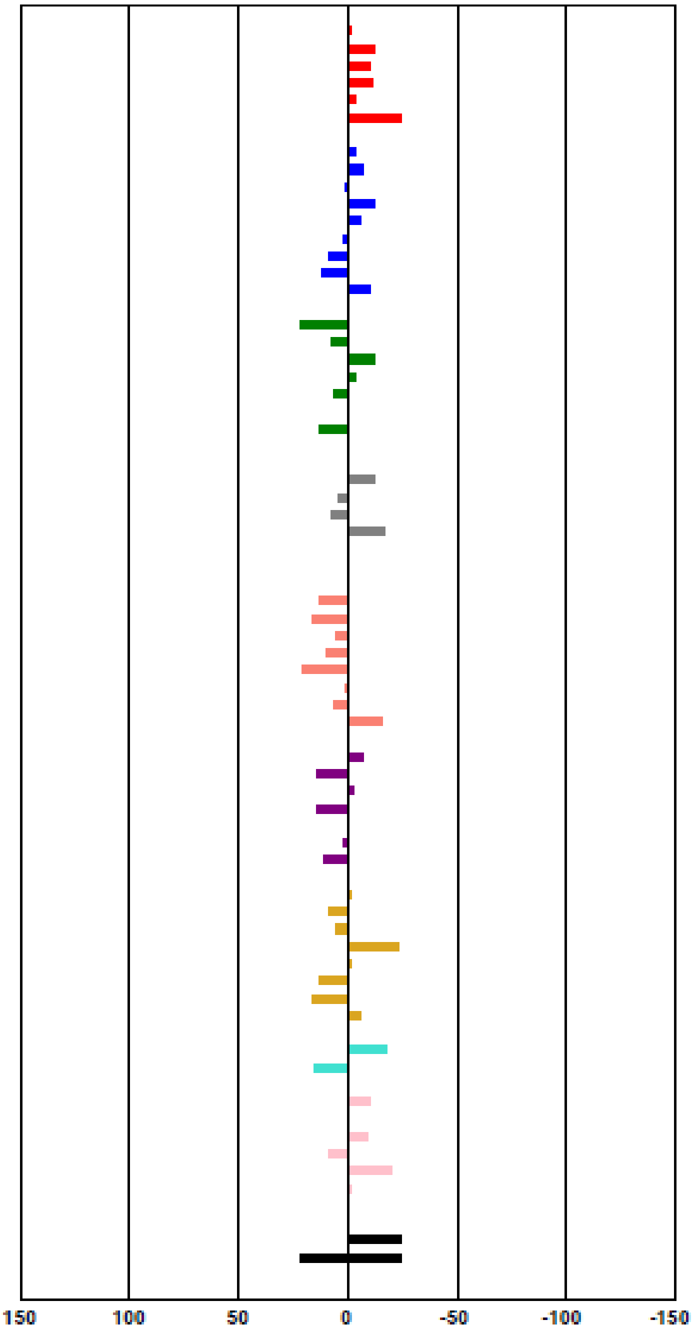

Figure S54. One dose mean graph for compound **2b** (NSC 827597) at 10 µM concentration

Developmental Therapeutics Program One Dose Mean Graph	NSC: D-827598 / 1	Conc: 1.00E-5 Molar	Test Date: Jan 11, 2021
	Experiment ID: 2101OS77		Report Date: Feb 02, 2021

Panel/Cell Line	Growth Percent
**Leukemia**	
CCRF-CEM	98.27
HL-60(TB)	94.65
K-562	100.17
MOLT-4	98.92
RPMI-8226	88.00
SR	84.99
**Non-Small Cell Lung Cancer**	
A549/ATCC	100.77
EKVX	94.17
HOP-62	98.53
HOP-92	89.76
NCI-H226	93.88
NCI-H23	98.62
NCI-H322M	100.94
NCI-H460	108.95
NCI-H522	91.40
**Colon Cancer**	
COLO 205	117.48
HCC-2998	103.01
HCT-116	98.04
HCT-15	106.39
HT29	105.70
KM12	99.43
SW-620	112.67
**CNS Cancer**	
SF-268	104.59
SF-295	92.08
SF-539	104.38
SNB-19	102.28
SNB-75	72.74
U251	101.28
**Melanoma**	
LOX IMVI	95.31
MALME-3M	97.58
M14	106.84
MDA-MB-435	103.01
SK-MEL-2	109.20
SK-MEL-28	113.26
SK-MEL-5	97.66
UACC-257	104.62
UACC-62	94.49
**Ovarian Cancer**	
IGROV1	99.08
OVCAR-3	115.33
OVCAR-4	90.42
OVCAR-5	111.33
OVCAR-8	99.74
NCI/ADR-RES	99.88
SK-OV-3	110.53
**Renal Cancer**	
786-0	93.30
A498	111.29
ACHN	98.87
CAKI-1	84.73
RXF 393	105.67
SN12C	109.67
TK-10	116.42
UO-31	91.53
**Prostate Cancer**	
PC-3	81.97
DU-145	113.84
**Breast Cancer**	
MCF7	86.42
MDA-MB-231/ATCC	97.93
HS 578T	98.01
BT-549	106.01
T-47D	87.57
MDA-MB-468	100.81
Mean	99.91
Delta	27.17
Range	44.74

Mean Growth Percent - Growth Percent

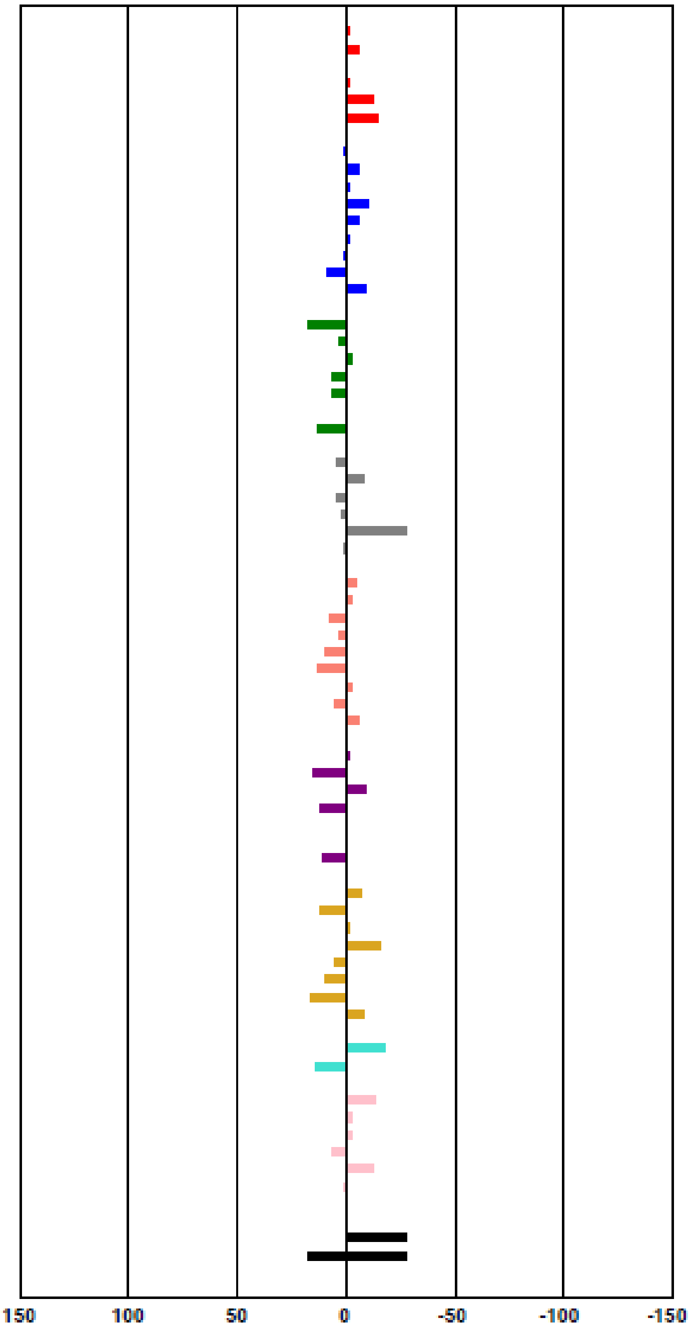

Figure S55. One dose mean graph for compound **2c** (NSC 827598) at 10 μM concentration

Developmental Therapeutics Program One Dose Mean Graph	NSC: D-826910 / 1	Conc: 1.00E-5 Molar	Test Date: Nov 30, 2020
	Experiment ID: 2011OS67		Report Date: Dec 15, 2020

Panel/Cell Line	Growth Percent
**Leukemia**	
CCRF-CEM	100.05
HL-60(TB)	94.73
K-562	92.95
MOLT-4	96.79
RPMI-8226	108.76
SR	98.17
**Non-Small Cell Lung Cancer**	
A549/ATCC	104.86
EKVX	97.13
HOP-62	106.62
HOP-92	112.40
NCI-H226	101.73
NCI-H23	100.08
NCI-H322M	101.80
NCI-H460	100.76
NCI-H522	93.89
**Colon Cancer**	
COLO 205	120.57
HCC-2998	99.64
HCT-116	99.75
HCT-15	105.94
HT29	104.93
KM12	99.00
SW-620	99.36
**CNS Cancer**	
SF-268	98.96
SF-295	110.84
SF-539	101.57
SNB-19	101.86
SNB-75	84.22
U251	98.77
**Melanoma**	
LOX IMVI	99.84
MALME-3M	97.85
M14	101.02
MDA-MB-435	101.41
SK-MEL-2	97.09
SK-MEL-28	106.11
SK-MEL-5	99.43
UACC-257	103.04
UACC-62	98.37
**Ovarian Cancer**	
IGROV1	105.13
OVCAR-3	108.01
OVCAR-4	106.80
OVCAR-5	109.85
OVCAR-8	104.57
NCI/ADR-RES	101.09
SK-OV-3	120.02
**Renal Cancer**	
786-0	99.11
A498	87.45
ACHN	99.84
CAKI-1	93.43
RXF 393	103.52
SN12C	95.81
TK-10	107.75
UO-31	94.04
**Prostate Cancer**	
PC-3	95.44
DU-145	109.42
**Breast Cancer**	
MCF7	98.59
MDA-MB-231/ATCC	99.54
HS 578T	96.65
BT-549	110.70
T-47D	101.81
MDA-MB-468	96.10
Mean	101.40
Delta	17.18
Range	36.35

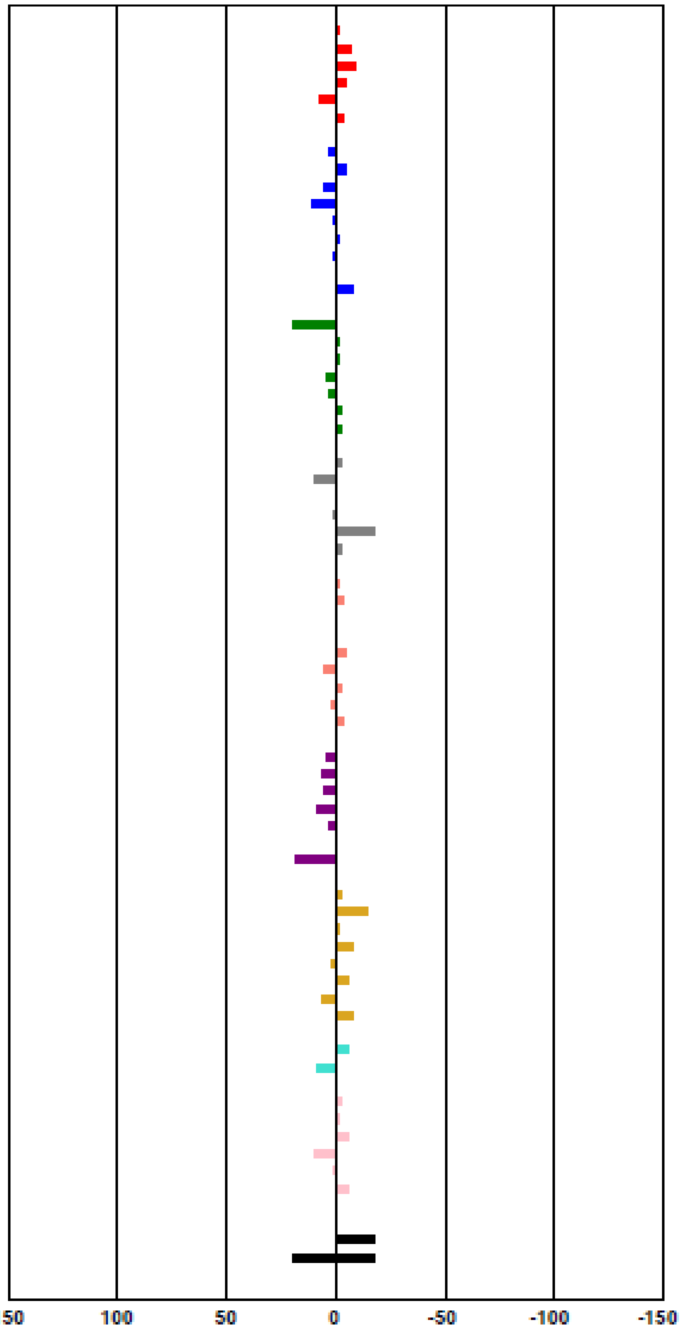

Figure S56. One dose mean graph for compound **2d** (NSC 826910) at 10 µM concentration

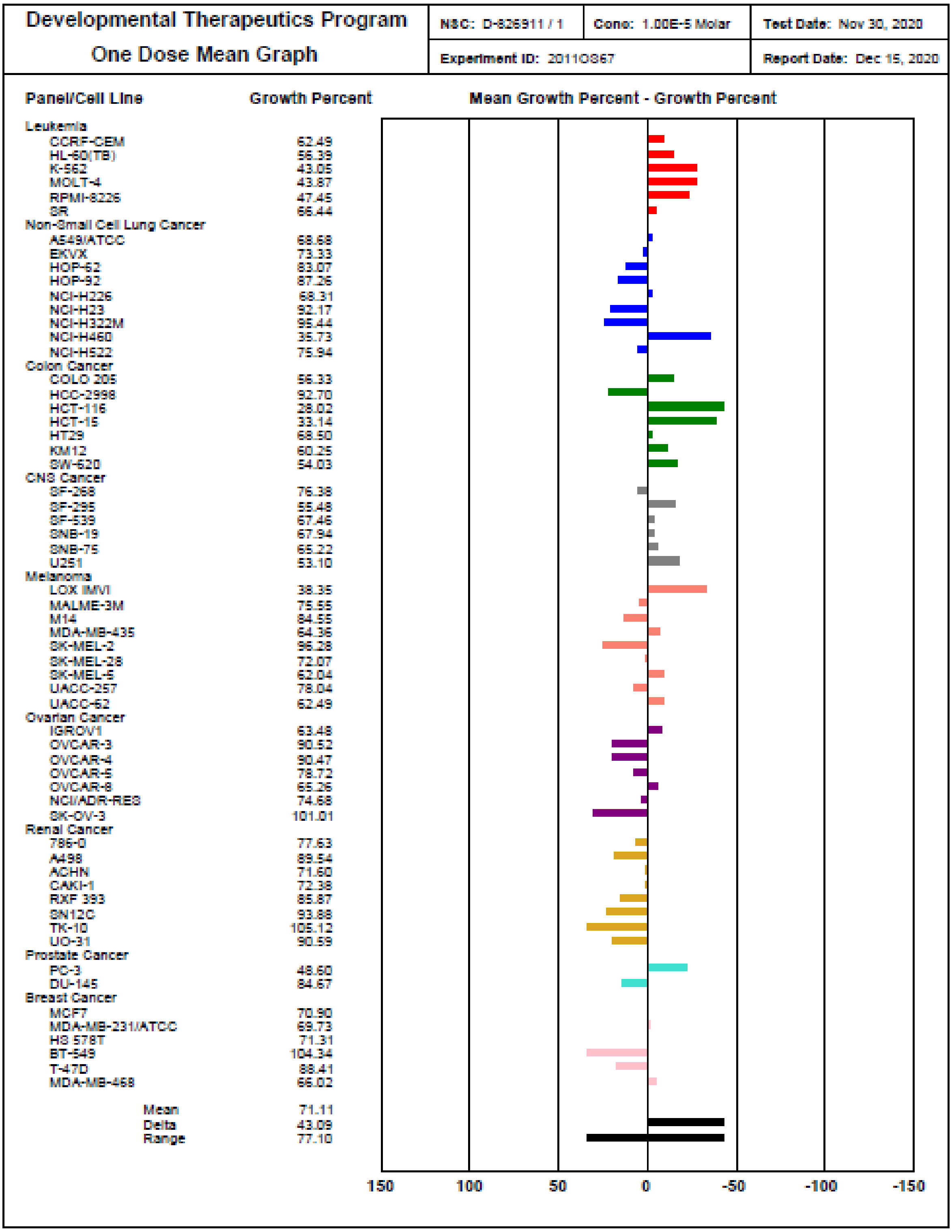

Figure S57. One dose mean graph for compound **2e** (NSC 826911) at 10 µM concentration

Developmental Therapeutics Program<br>One Dose Mean Graph	NSC: D-827601 / 1	Conc: 1.00E-5 Molar	Test Date: Jan 11, 2021
	Experiment ID: 2101OS77		Report Date: Feb 02, 2021

Panel/Cell Line	Growth Percent
**Leukemia**	
CCRF-CEM	92.14
HL-60(TB)	88.88
K-562	93.91
MOLT-4	85.86
RPMI-8226	87.60
SR	91.31
**Non-Small Cell Lung Cancer**	
A549/ATCC	96.59
EKVX	96.13
HOP-62	90.43
HOP-92	103.50
NCI-H226	98.54
NCI-H23	107.45
NCI-H322M	93.86
NCI-H460	109.93
NCI-H522	96.64
**Colon Cancer**	
COLO 205	109.52
HCC-2998	112.37
HCT-116	88.47
HCT-15	91.91
HT29	110.20
KM12	98.22
SW-620	102.73
**CNS Cancer**	
SF-268	98.52
SF-295	98.42
SF-539	101.89
SNB-19	96.63
SNB-75	62.78
U251	98.43
**Melanoma**	
LOX IMVI	97.74
MALME-3M	109.91
M14	112.71
MDA-MB-435	102.84
SK-MEL-2	113.76
SK-MEL-28	110.37
SK-MEL-5	95.06
UACC-257	100.97
UACC-62	78.65
**Ovarian Cancer**	
IGROV1	92.24
OVCAR-3	112.25
OVCAR-4	104.90
OVCAR-5	106.33
OVCAR-8	102.05
NCI/ADR-RES	101.16
SK-OV-3	106.39
**Renal Cancer**	
786-0	98.68
A498	103.34
ACHN	99.74
CAKI-1	81.84
RXF 393	101.01
SN12C	102.15
TK-10	112.10
UO-31	86.39
**Prostate Cancer**	
PC-3	76.76
DU-145	109.86
**Breast Cancer**	
MCF7	93.72
MDA-MB-231/ATCC	96.17
HS 578T	97.14
BT-549	114.70
T-47D	93.22
MDA-MB-468	98.11
Mean	98.59
Delta	35.81
Range	51.92

Mean Growth Percent - Growth Percent

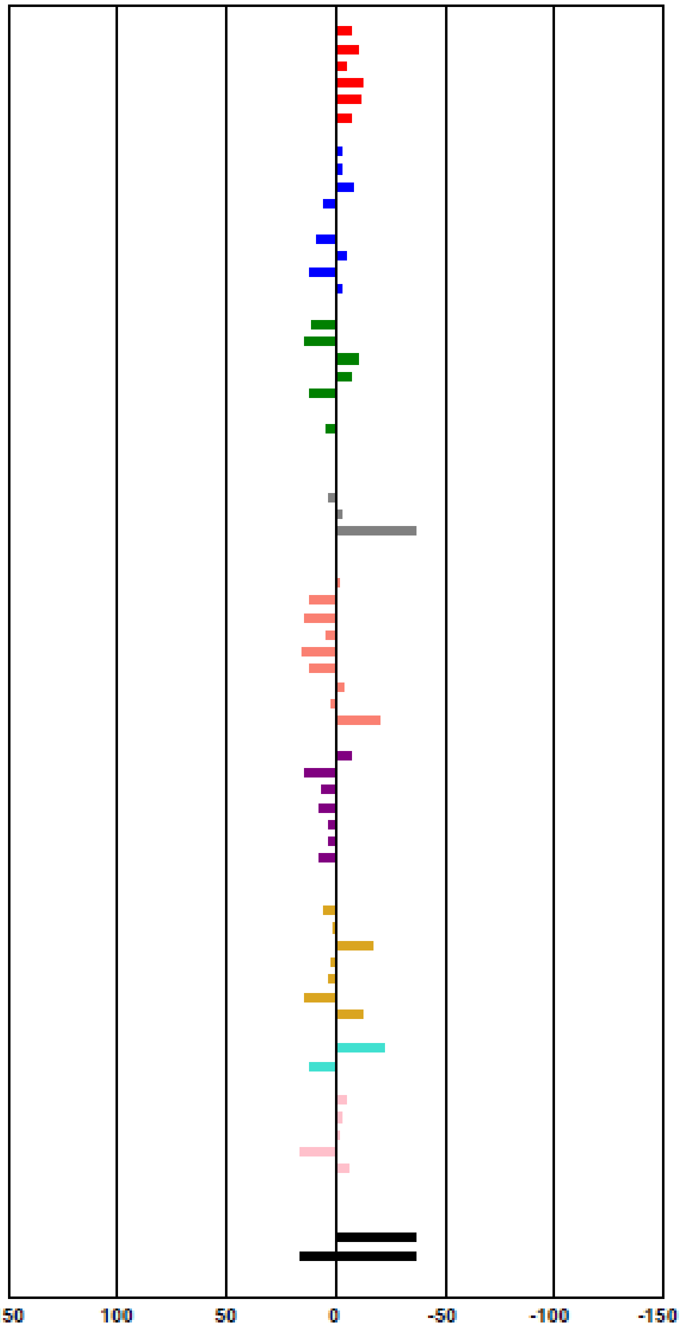

Figure S58. One dose mean graph for compound **2f** (NSC 827601) at 10 µM concentration

Developmental Therapeutics Program One Dose Mean Graph	NSC: D-827596 / 1	Conc: 1.00E-5 Molar	Test Date: Jan 11, 2021
	Experiment ID: 2101OS77		Report Date: Feb 02, 2021

Panel/Cell Line	Growth Percent
**Leukemia**	
CCRF-CEM	103.50
HL-60(TB)	101.50
K-562	98.46
MOLT-4	103.88
RPMI-8226	101.14
SR	85.93
**Non-Small Cell Lung Cancer**	
A549/ATCC	101.33
EKVX	95.84
HOP-62	98.96
HOP-92	94.90
NCI-H226	99.55
NCI-H23	98.24
NCI-H322M	104.64
NCI-H460	110.73
NCI-H522	91.11
**Colon Cancer**	
COLO 205	123.35
HCC-2998	102.82
HCT-116	109.69
HCT-15	95.02
HT29	101.10
KM12	97.03
SW-620	108.30
**CNS Cancer**	
SF-268	100.23
SF-295	93.16
SF-539	104.96
SNB-19	104.02
SNB-75	98.71
U251	99.46
**Melanoma**	
LOX IMVI	93.18
MALME-3M	106.14
M14	107.56
MDA-MB-435	96.36
SK-MEL-2	100.56
SK-MEL-28	109.21
SK-MEL-5	100.98
UACC-257	102.78
UACC-62	91.71
**Ovarian Cancer**	
IGROV1	108.49
OVCAR-3	112.64
OVCAR-4	98.60
OVCAR-5	102.01
OVCAR-8	99.73
NCI/ADR-RES	98.98
SK-OV-3	106.91
**Renal Cancer**	
786-0	98.76
A498	101.22
ACHN	101.78
CAKI-1	96.47
RXF 393	99.35
SN12C	106.69
TK-10	100.98
UO-31	96.40
**Prostate Cancer**	
PC-3	91.76
DU-145	109.17
**Breast Cancer**	
MCF7	88.63
MDA-MB-231/ATCC	101.45
HS 578T	89.35
BT-549	108.97
T-47D	90.40
MDA-MB-468	96.42
Mean	100.69
Delta	14.76
Range	37.42

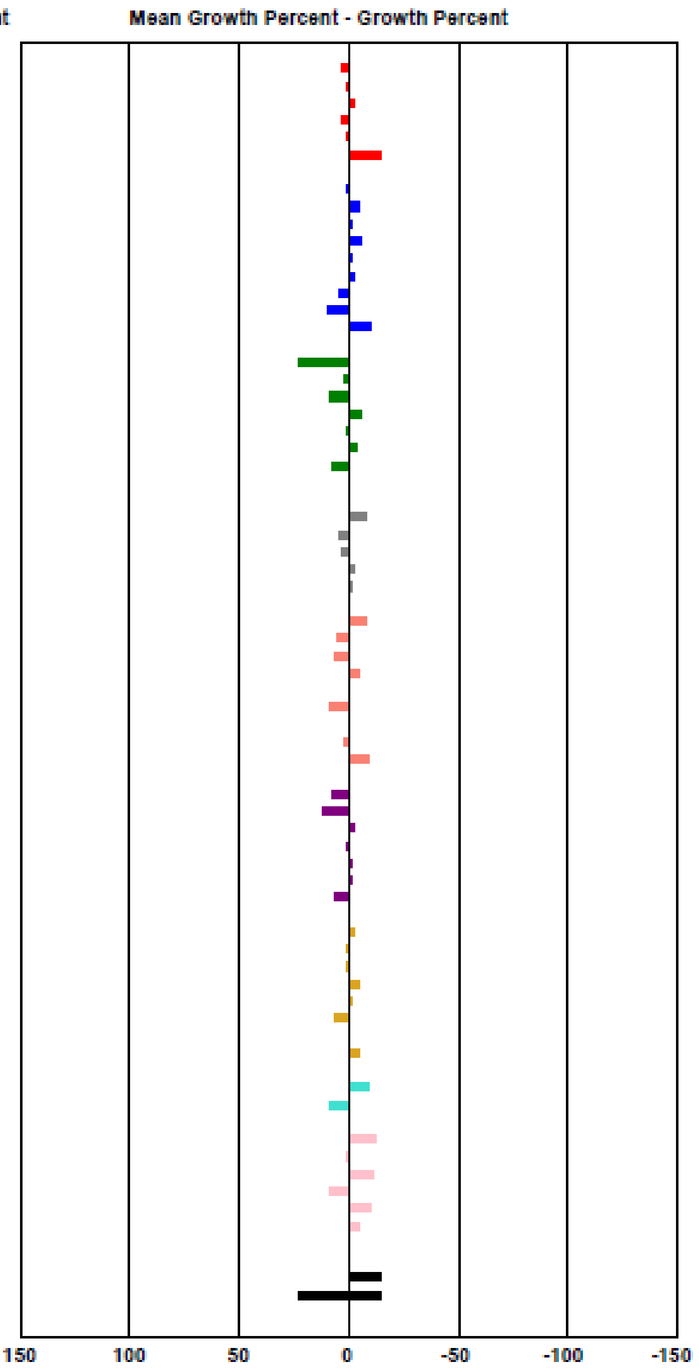

Figure S59. One dose mean graph for compound **2g** (NSC 827596) at 10 µM concentration

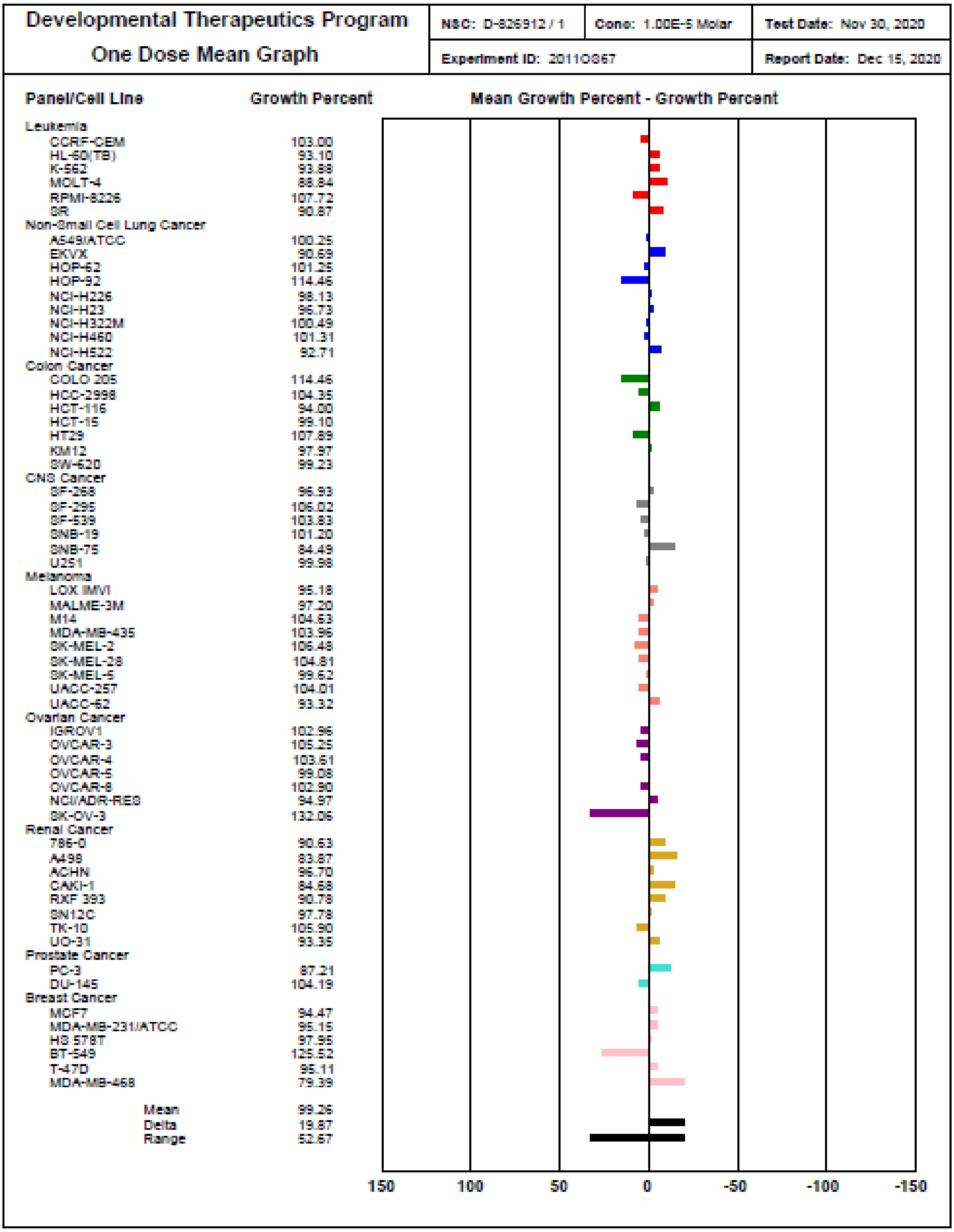

Figure S60. One dose mean graph for compound **2h** (NSC 826912) at 10 µM concentration

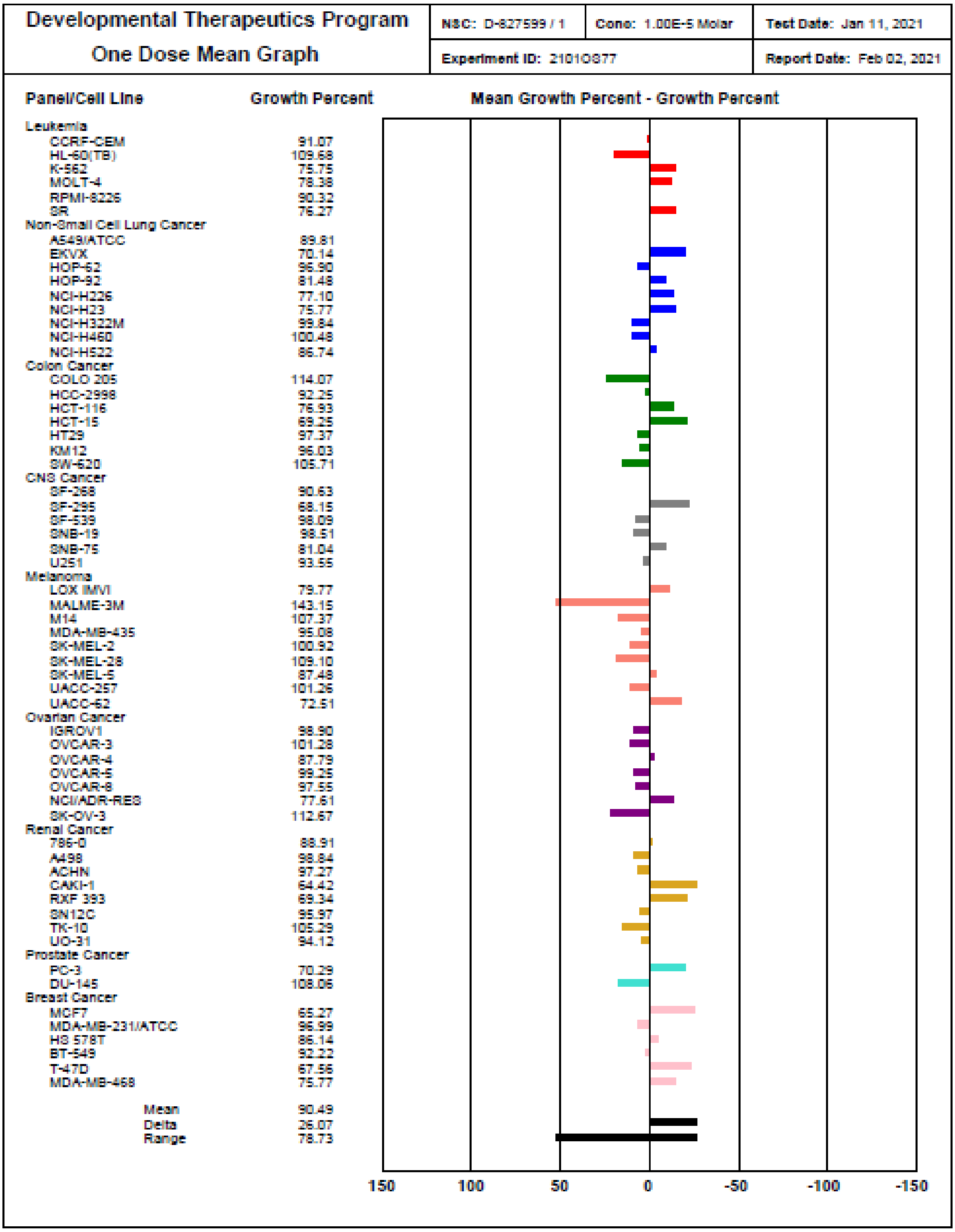

Figure S61. One dose mean graph for compound **2i** (NSC 827599) at 10 μM concentration

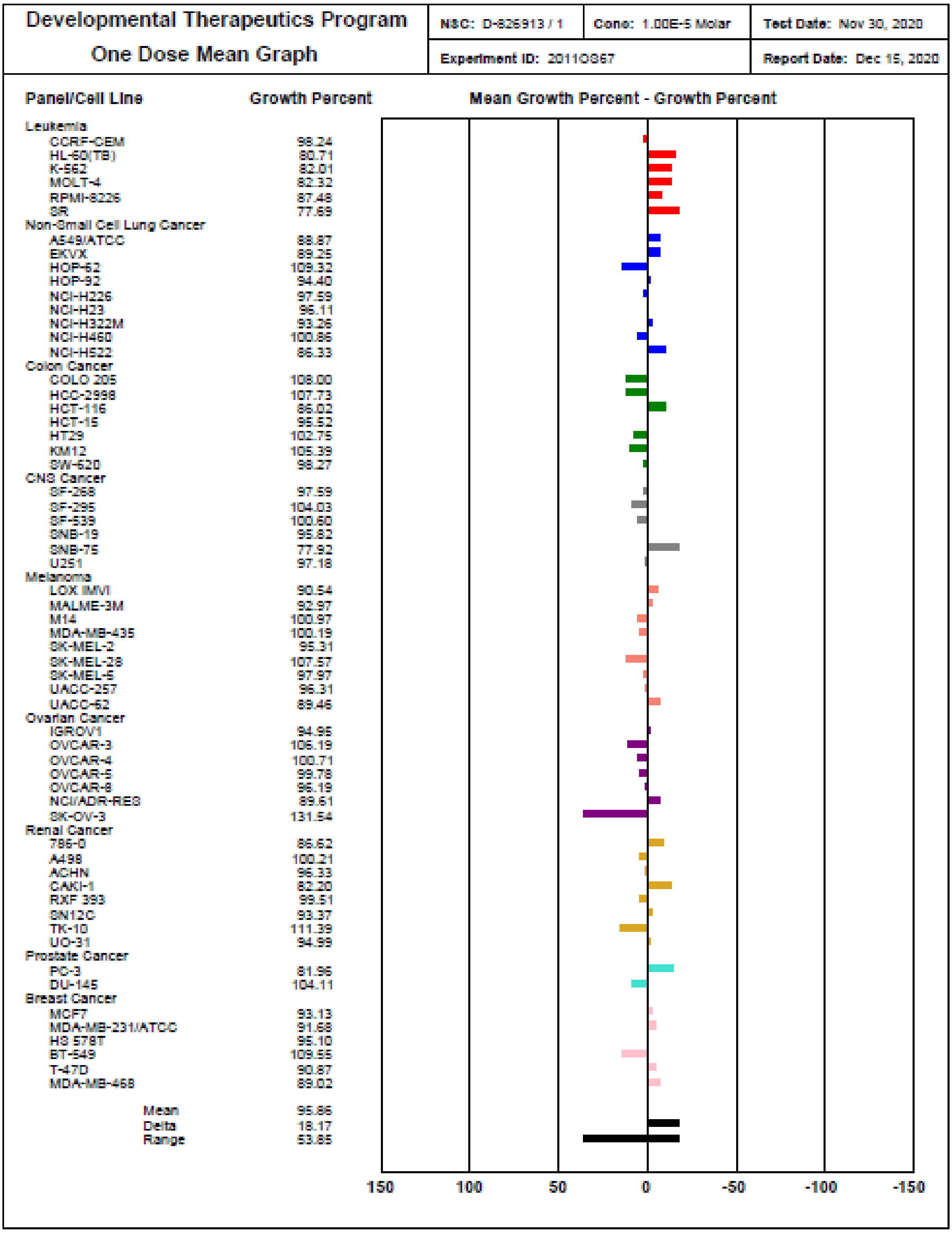

Figure S62. One dose mean graph for compound **2j** (NSC 826913) at 10 µM concentration

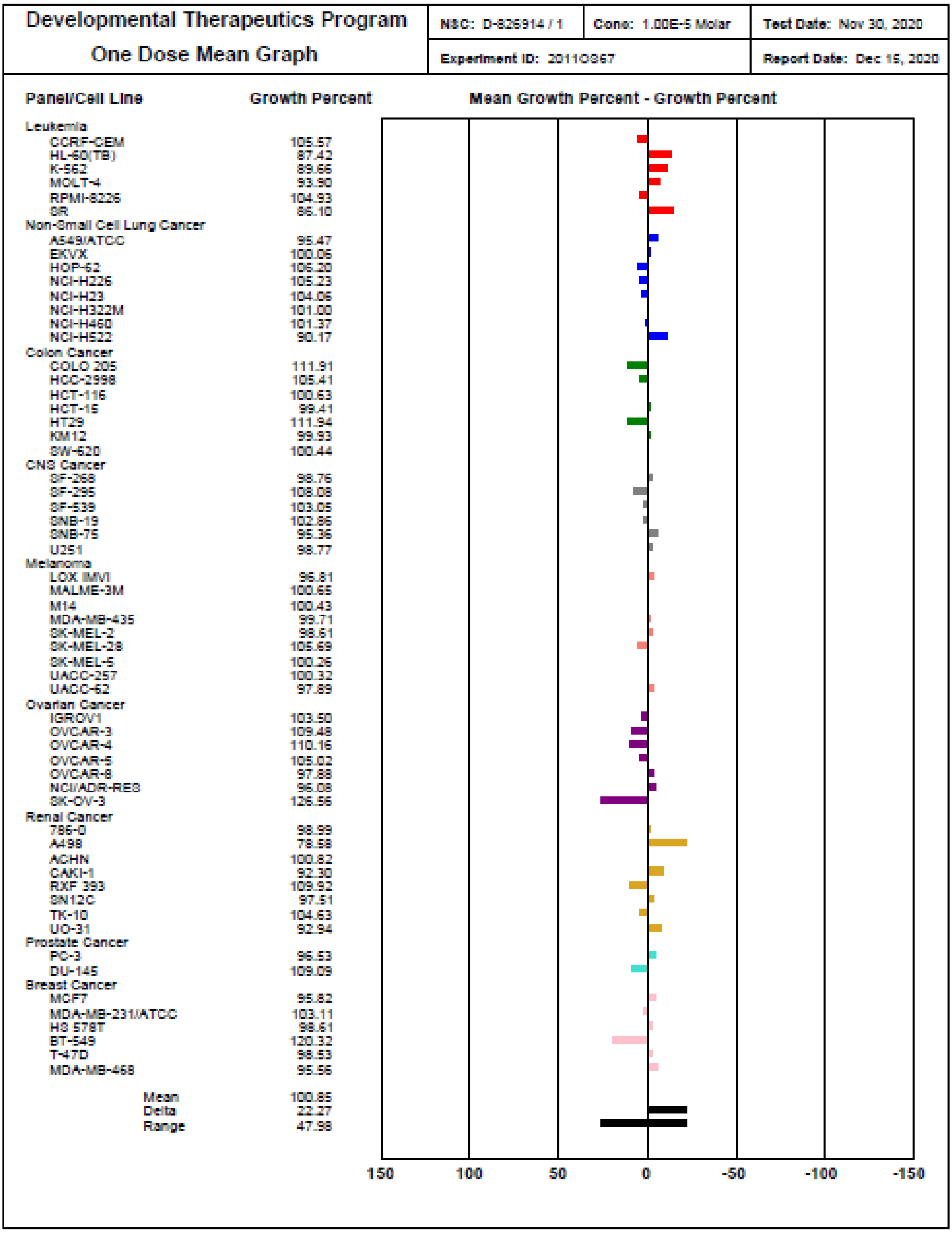

Figure S63. One dose mean graph for compound **2k** (NSC 826914) at 10 µM concentration

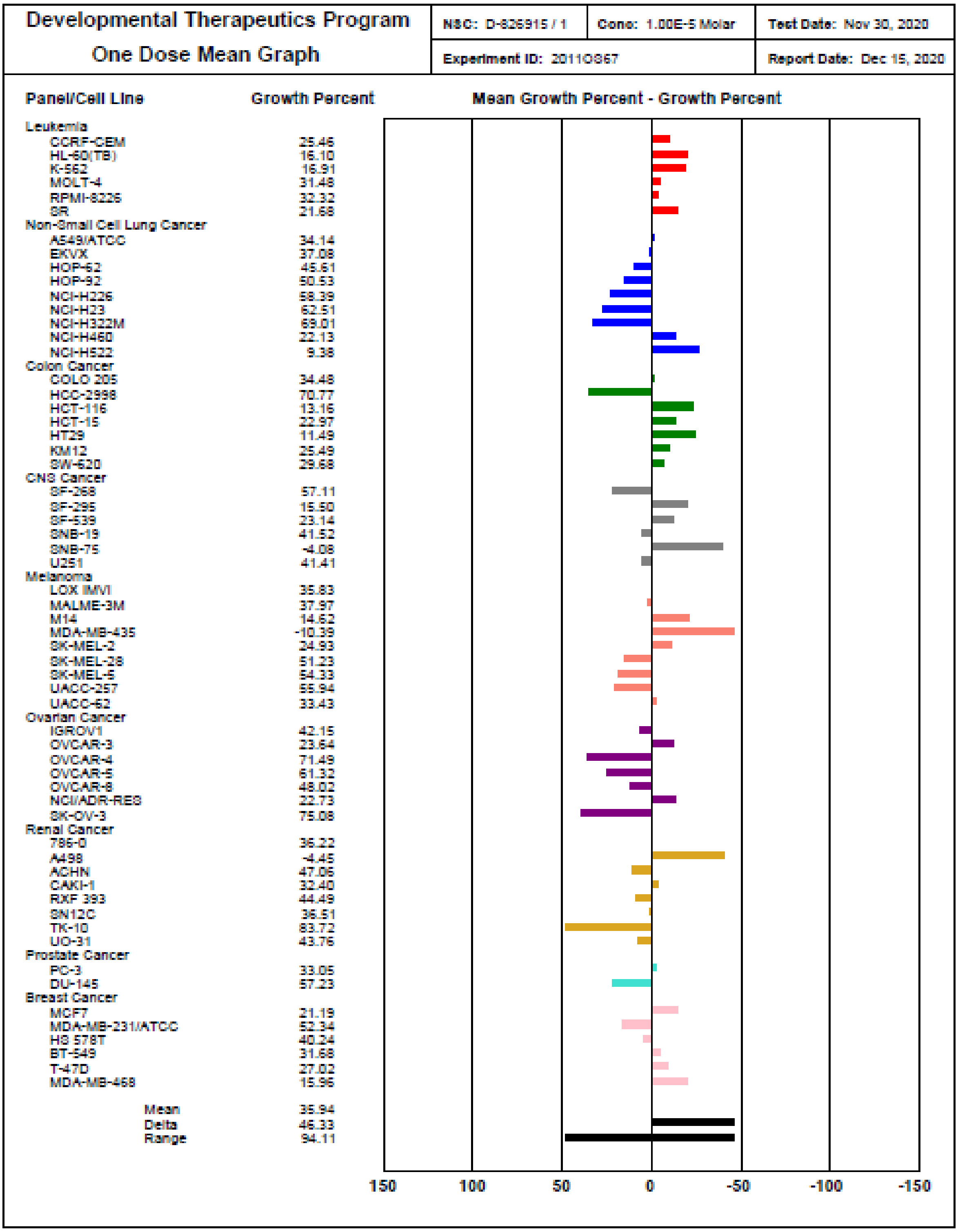

Figure S64. One dose mean graph for compound **2l** (NSC 826915) at 10 µM concentration

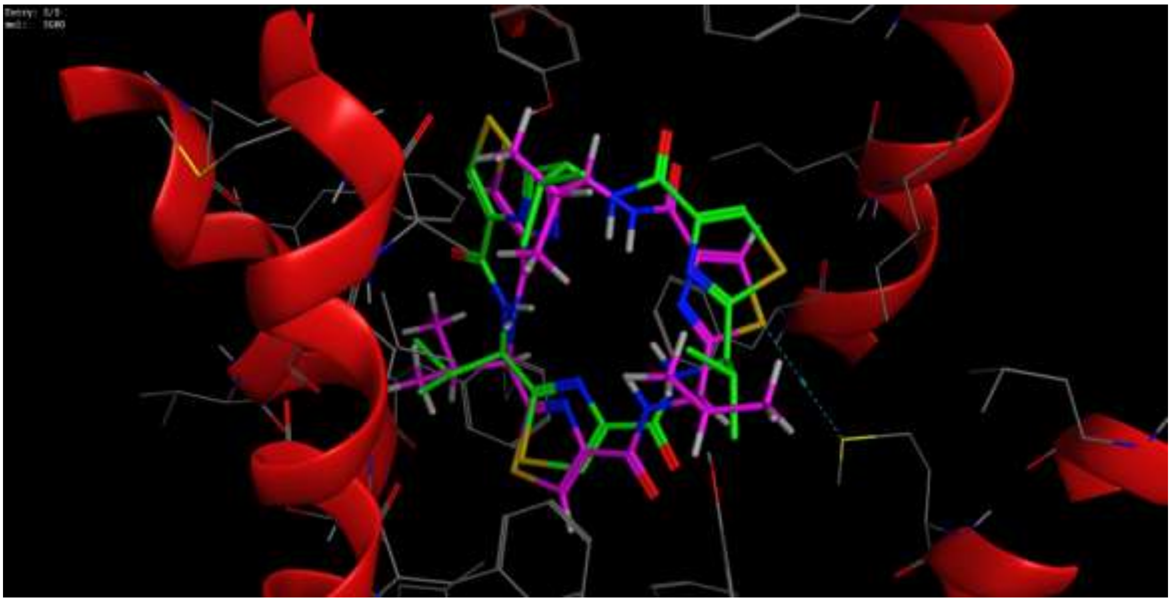

Figure S64. Overlay of re-docked ligand (violet color) and co-crystalized ligand (green color) in the P-gp active site (PDB: 3G60).

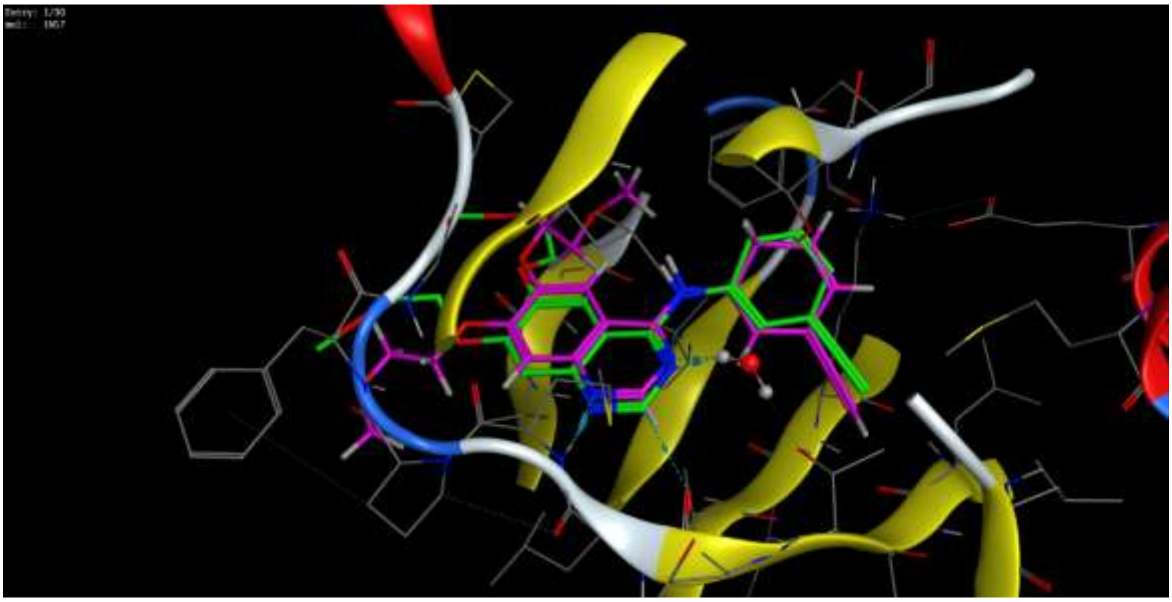

Figure S65. Overlay of re-docked ligand (violet color) and co-crystalized ligand (green color) in the EGFR active site (PDB: 1M17).

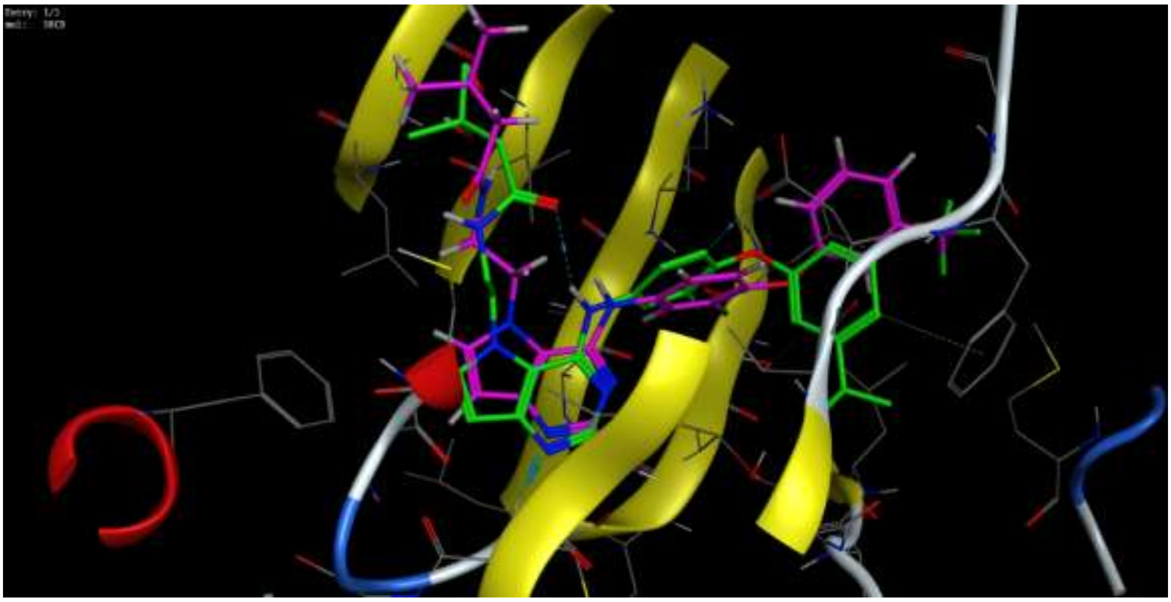

Figure S66. Overlay of re-docked ligand (violet color) and co-crystalized ligand (green color) in the HER-2 active site (PDB: 3RCD).

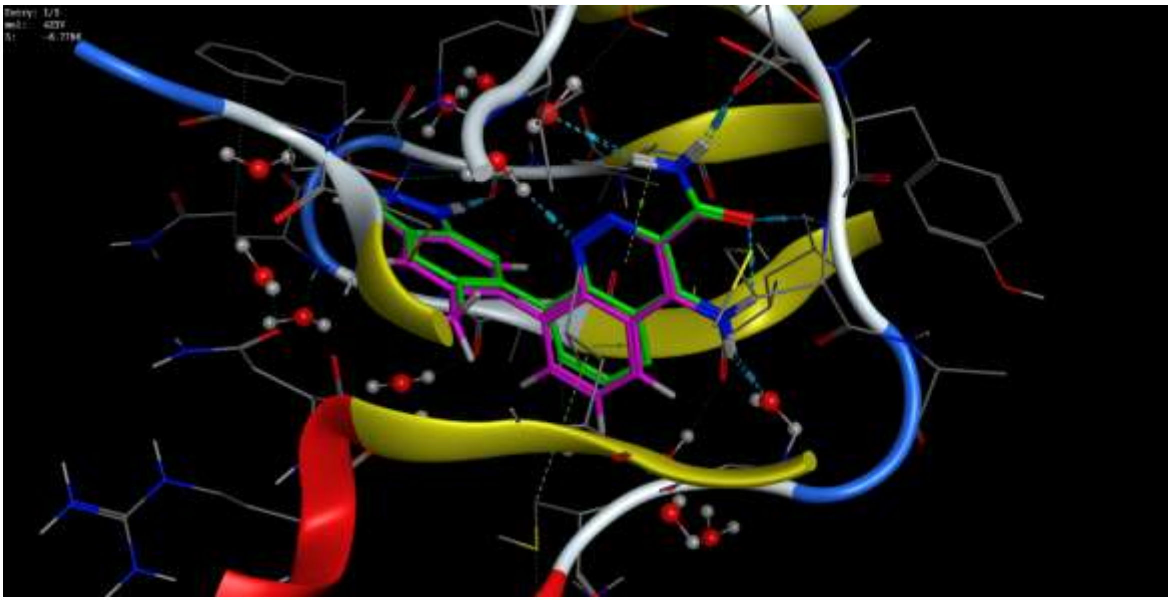

Figure S67. Overlay of re-docked ligand (violet color) and co-crystalized ligand (green color) in the BTK active site (PDB: 4Z3V).

References

1. Hantzsch A. Ueber die synthese pyridinartiger verbindungen aus acetessigäther und aldehydammoniak. Justus Liebigs Annalen der Chemie. 1882;215(1):1-82.
2. Alvim HG, da Silva Junior EN, Neto BA. What do we know about multicomponent reactions? Mechanisms and trends for the Biginelli, Hantzsch, Mannich, Passerini and Ugi MCRs. Rsc Advances. 2014;4(97):54282-54299.
3. Santos VG, Godoi MN, Regiani T, et al. The Multicomponent Hantzsch Reaction: Comprehensive Mass Spectrometry Monitoring Using Charge-Tagged Reagents. Chemistry–A European Journal. 2014;20(40):12808-12816.
4. Katritzky AR, Ostercamp DL, Yousaf TI. The mechanism of the Hantzsch pyridine synthesis: A study by 15N and 13C NMR spectroscopy. Tetrahedron. 1986;42(20):5729-5738.
5. Saini A, Kumar S, Sandhu JS. Hantzsch reaction: Recent advances in Hantzsch 1, 4-dihydropyridines. 2008;67:95-111.
6. IWANAMI M, Shibanuma T, FUJIMOTO M, et al. Synthesis of new water-soluble dihydropyridine vasodilators. Chemical and Pharmaceutical Bulletin. 1979;27(6):1426-1440.
7. Miri R, Javidnia K, Kebriaie-Zadeh A, et al. Synthesis and evaluation of pharmacological activities of 3, 5-dialkyl 1, 4-dihydro-2, 6-dimethyl-4-nitroimidazole-3, 5-pyridine dicarboxylates. Archiv der Pharmazie: An International Journal Pharmaceutical and Medicinal Chemistry. 2003;336(9):422-428.

Information Classification: General
